# Chemotaxis in external fields: Simulations for active magnetic biological matter

**DOI:** 10.1371/journal.pcbi.1007548

**Published:** 2019-12-19

**Authors:** Agnese Codutti, Klaas Bente, Damien Faivre, Stefan Klumpp

**Affiliations:** 1 Department Biomaterials, Max Planck Institute of Colloids and Interfaces, Potsdam, Germany; 2 Department Theory and Bio-Systems, Max Planck Institute of Colloids and Interfaces, Potsdam, Germany; 3 University of Potsdam, Institute of Physics and Astronomy, Potsdam, Germany; 4 Aix Marseille University, CNRS, CEA, BIAM, 13108 Saint Paul lez Durance, France; 5 Institute for the Dynamics of Complex Systems, University of Göttingen, Göttingen, Germany; Rice University, UNITED STATES

## Abstract

The movement of microswimmers is often described by active Brownian particle models. Here we introduce a variant of these models with several internal states of the swimmer to describe stochastic strategies for directional swimming such as run and tumble or run and reverse that are used by microorganisms for chemotaxis. The model includes a mechanism to generate a directional bias for chemotaxis and interactions with external fields (e.g., gravity, magnetic field, fluid flow) that impose forces or torques on the swimmer. We show how this modified model can be applied to various scenarios: First, the run and tumble motion of *E. coli* is used to establish a paradigm for chemotaxis and investigate how it is affected by external forces. Then, we study magneto-aerotaxis in magnetotactic bacteria, which is biased not only by an oxygen gradient towards a preferred concentration, but also by magnetic fields, which exert a torque on an intracellular chain of magnets. We study the competition of magnetic alignment with active reorientation and show that the magnetic orientation can improve chemotaxis and thereby provide an advantage to the bacteria, even at rather large inclination angles of the magnetic field relative to the oxygen gradient, a case reminiscent of what is expected for the bacteria at or close to the equator. The highest gain in chemotactic velocity is obtained for run and tumble with a magnetic field parallel to the gradient, but in general a mechanism for reverse motion is necessary to swim against the magnetic field and a run and reverse strategy is more advantageous in the presence of a magnetic torque. This finding is consistent with observations that the dominant mode of directional changes in magnetotactic bacteria is reversal rather than tumbles. Moreover, it provides guidance for the design of future magnetic biohybrid swimmers.

## Author summary

In this paper, we propose a modified Active Brownian particle model to describe bacterial swimming behavior under the influence of external forces and torques, in particular of a magnetic torque. This type of interaction is particularly important for magnetic biohybrids (i.e. motile bacteria coupled to a synthetic magnetic component) and for magnetotactic bacteria (i.e. bacteria with a natural intracellular magnetic chain), which perform chemotaxis to swim along chemical gradients, but are also directed by an external magnetic field. The model allows us to investigate the benefits and disadvantages of such coupling between two different directionality mechanisms. In particular we show that the magnetic torque can speed chemotaxis up in some conditions, while it can hinder it in other cases. In addition to an understanding of the swimming strategies of naturally magnetotactic organisms, the results may guide the design of future biomedical devices.

## Introduction

The motion of many microorganisms as well as synthetic and biohybrid microswimmers is based on a directed self-propulsion over short time and length scale. On larger scales, however, these swimmers typically perform (persistent) random walks due to either the Brownian rotation of their direction of propulsion, which is unavoidable due to their small size, or due to active mechanisms of re-orientation such as the tumbling of bacteria [1–3]. Biasing this random motion is key to directional motion on large scales. The paradigm for such directional guidance is the chemotaxis of bacteria such as *Escherichia coli*, which alternate between straight runs and abrupt changes of direction called tumbles. Chemosensing and intracellular signaling to the flagella bias the resulting random walk by prolonging runs in the direction up a gradient of a chemoattractant [3, 4].

Other mechanisms of steering microswimmers make use of external fields that orient the swimming. Specifically, homogeneous magnetic fields are a promising steering mechanism for microswimmers that are equipped with a magnetic moment, a situation that occurs naturally in magnetotactic bacteria [5–7], but is also used in a variety of synthetic and biohybrid swimmers [8–10]. A number of interesting questions relate to the combination of two mechanisms of directional guidance: Which strategies do microorganisms use to resolve conflicts between different directional inputs such as chemotaxis and external forces? Which strategies could be implemented in synthetic systems?

Magnetotactic bacteria are a natural example of bacteria undergoing both chemotaxis and magnetic interactions: the passive alignment to the Earth magnetic field of the intracellular magnetic chain of these bacteria is usually intertwined with aerotaxis, i.e. chemotaxis for oxygen [5–7, 11, 12]. Still, the interaction with such a weak field does not provide perfect alignment. What is the advantage given by magnetic fields to the magnetotactic bacteria? Previous studies approached these questions at the population level, both from an experimental and computational point of view, and suggested that the magnetic orientation may provide an advantage for the tactic dynamics of the bacteria [13–15]. These studies however, do not answer the question whether this is always the case, e.g. if the chemical gradient and the magnetic field steer the bacteria in very different directions, and have not addressed the dependence on the chemotactic strategy. The latter question is also important for biohybrids, where a chemotactic strategy that is not adapted to work with a magnetic torque is combined with magnetic steering. Such chemotactic biohybrid swimmer have been realized very recently, by functionalizing *E. coli* bacteria with magnetic beads [16]. Magnetically steerable biohybrids or functionalized magnetotactic bacteria are envisioned for biomedical applications such as drug delivery and cancer or biofilms targeting [7, 17, 18]. So far, these applications are at the proof-of-principle state [7, 17, 18], but eventually, understanding how magnetic fields or other external forces interact with chemotaxis may help in the rational design of such systems.

**Fig 1:**
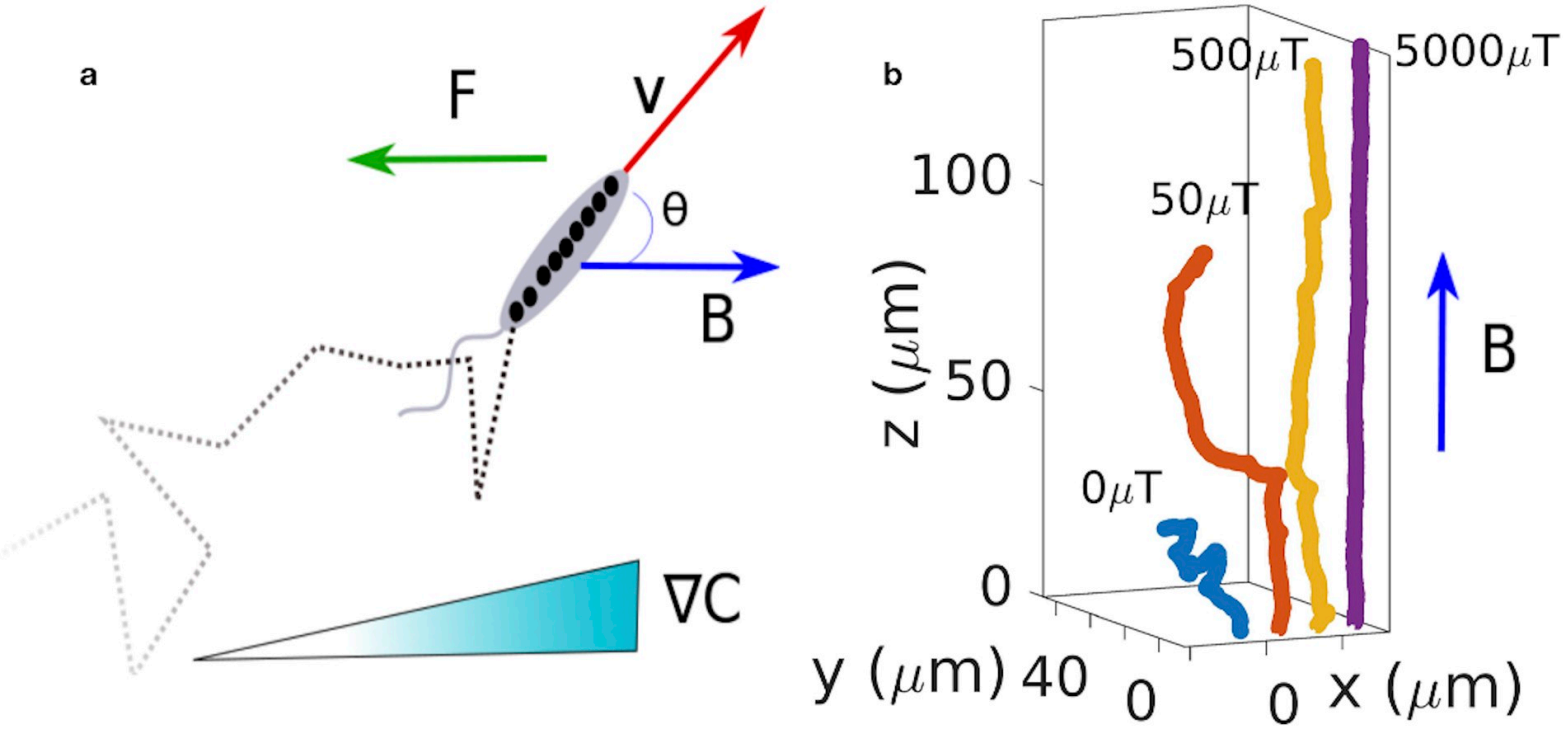
Model for swimming and chemotaxis in external fields. a) The model describes a bacterium that actively swims with velocity v, and that is subject to an external force *F* and a magnetic field *B* (measured in microTesla, *μ*T). Its random walk may also be biased to perform chemotaxis in a concentration gradient ∇*C* thanks to active changes of direction such as tumbles or reversals. b) Examples trajectories for run and tumble motion in the presence of a magnetic field. The magnetic interaction helps to reduce the motion of the bacteria to an effectively one-dimensional motion and directs it in the direction of the magnetic field. Active directional changes (tumbles) result in swimming in other directions, an effect that is increasingly suppressed with increasing intensity of the magnetic field.

In this work, we introduce a theoretical approach to address these questions at the micrometer scale. Two types of theoretical approaches have been used to describe the persistent random walks of microswimmers: bacterial chemotaxis has been described by random walk models, which provide a rather accurate description of the bacterial trajectories with abrupt turns due to tumbling [3, 19, 20]. Tumbling is usually not included in the active Brownian particle models commonly used to describe self-propelled particles. Active Brownian particle models, however allow for the straightforward incorporation of external forces and have therefore been used extensively to study interactions between active particles and the resulting collective effects [2, 21]. Here, we combine features of both approaches into a multi-state active Brownian particle model for chemotactic motion. Our approach uses a Langevin equation of motion, in which external forces or torques are easily included (See Fig 1a), but also accounts for the abrupt changes of motion (run and tumble, run and reverse, etc.) characteristic for the motion of many microorganism. Similar approaches have occasionally been used [22, 23], including one study incorporating external forces [24] and one incorporating surface attachment as an additional state of the swimmer [25].

Here, we use this model as a general approach to chemotaxis under the influence of external forces and torques and determine how such forces and torques influence the chemotactic velocity. Specifically, we apply it to the magneto-aerotaxis of magnetotactic bacteria and study the competition of orientation changes with the alignment with the field. Moreover, we compare the ‘run and reverse’ and ‘run and tumble’ strategies for chemotaxis. We show that run and reverse allows for magneto-aerotaxis at arbitrary orientations of the magnetic field relative to the gradient, while a higher speed-up of chemotaxis by the field can be reached for run and tumble, but only for limited configuration of field to gradient orientations. In particular, the presence of a magnetic field can speed up the formation of a magneto-aerotactic band around a preferred oxygen concentration in the case of run and reverse, while band formation is prevented by a magnetic field for run and tumble. Our results indicate that magnetic orientation of chemotactic cells is beneficial for the cells under a wide range of conditions, but not universally so, and that the choice of chemotactic strategy is crucial to benefit from a magnetic orientation.

## Materials and methods

### Theoretical model and simulation

To describe bacterial chemotaxis subject to external fields, we use an active Brownian particle model with multiple internal states (e.g., “run” and “change of direction”). The bacteria are described as point particles with position **r** and a direction vector **e** that determines the direction of self-propulsion. In each state, their dynamics is given by Langevin equations:

$$\begin{array}{ccc} \gamma_{\text{t}}\frac{\text{d}r}{\text{d}t} & = & \gamma_{\text{t}}\sigma ve+F_{\text{ext}}+\sqrt{2k_{\text{B}}T\gamma_{\text{t}}}\xi_{\text{t}}\\ \gamma_{\text{r}}\frac{\text{d}e}{\text{d}t} & = & [T_{\text{ext}}+\sqrt{2k_{\text{B}}T\gamma_{\text{r}}}\xi_{\text{r}}]\times e. \end{array}$$ (1)

In these equations, *k*_B_ is the Boltzmann constant, *γ*_t_ and *γ*_r_ are the translational and rotational friction coefficients, respectively, *v* is the speed of self propulsion, *σ* is the sign of the self-propulsion velocity (±1 for parallel or antiparallel to **e**, 0 for no self-propulsion), **F**_ext_ and **T**_ext_ describe external forces and torques and *ξ*_t_ and *ξ*_r_ describe uncorrelated white noise in the translational and rotational degrees of freedom. The translational noise is purely thermal with temperature *T*, but the rotational noise may have additional contributions. Specifically, we consider the case of run and tumble motion. In this case, *σ* = 1 during runs and *σ* = 0 during tumbles, corresponding to self-propulsion during runs and no self-propulsion during tumbles, respectively (see S1 Fig). Moreover, the rapid reorientation during tumbles is described by a noise strength that exceeds the thermal noise. We adopt a description of the orientation changes during tumbles by rotational diffusion with an effective temperature *T*_tumble_ > *T*. This temperature approximates the distribution of orientation changes [26] (but not the correlations of angular change and tumble duration [27]). Matching that distribution to the observed distribution for *E. coli* (with an average reorientation by 68°) [28] results in an effective temperature of *T*_tumble_ = 4.2 × 10^4^ K (see S2 Fig). Transitions between the run and tumble states are stochastic with exponentially distributed dwell times and occur with rates $k_{\text{run}}=\tau_{\text{run}}^{-1}$ and $k_{\text{tumble}}=\tau_{\text{tumble}}^{-1}$ [28]. The average tumble time *τ*_tumble_ is rather short (≃ 0.1 s) and constant, the average run time *τ*_run_ is longer (≃ 1 s) [28] and is modulated by a chemical gradient for chemotaxis. Without loss of generality, these chosen values are based on the widely-studied *E. coli* behavior [28]. Results can be easily scaled to other parameter choices. For a chemoattractant, we use the following simple implementation of that modulation (other functional dependencies can also be used [29, 30]):

$$\begin{array}{c} \tau_{\text{run}}=\{\begin{array}{cc} t_{\text{down}}=\tau_{0} & \text{for}\;\nabla C_{∥}\leq 0\\ \tau_{0}(1+\frac{\nabla C_{∥}}{\nabla C_{0}}) & \text{for}\;0<\nabla C_{∥}\leq \nabla C_{0}\\ t_{\text{up}}=2\tau_{0} & \text{for}\;\nabla C_{∥}>\nabla C_{0} \end{array} \end{array}$$ (2)

where *τ*_0_ indicates the mean run time in absence of gradients, *t*_up_ and *t*_down_ represent the positive-biased times and the negative one, ∇*C*_∥_ indicates the projection of the chemical gradient onto the direction of motion and ∇*C*_0_ is a threshold gradient for which the maximal run time is reached. *τ*_run_ can be interpreted as a response function to the gradient and concentration. See S1 Text for more details.

Run and reverse motion is described in a completely analogous way with three states, run (*σ* = 1), reversal pause (mimicking the slowing observed before reversals [13, 31, 32], with *σ* = 0), and reverse run (*σ* = −1) (see S1 Fig). Transitions occur in a cyclic fashion from run to pause, to reverse to pause to run with average durations *τ*_run_, *τ*_pause_ and *τ*_reverse_ respectively. In contrast to run and tumble, the orientational noise is thermal in all three states. Due to thermal rotational diffusion during the pause, the orientation of the cells changes on average by 170° in a reversal. Chemotaxis modulates *τ*_run_ and *τ*_reverse_ in the same way as in the case of run and tumble.

We emphasize that the models we use here do not include detailed descriptions of the chemotactic response. Specifically, signaling and adaptation are not described explicitly. Rather the model is based on a coarse-grained description of the random walk that arises from that signaling. Such an approach, while clearly being simplified and not accounting for all features of chemotactic behavior, has two advantages: On the one hand, such a coarse-grained description can also be used when the underlying chemotactic mechanisms are unknown. On the other hand, the coarse-grained description is also computationally less expensive when simulating large numbers of cells or trajectories over long time scales as we do here, in particular when we study the formation of magneto-aerotactic bands.

For most simulations with either scenario, we use the parameters estimated from the experimental data for *E. coli* from the literature [3, 28] (see S1 Table). Parameters are adjusted to match our simulation results to the experiments for magnetoaerotactic band formation of *M. gryphiswaldense* as described below/in Results (see S2 Table).

The simulations are performed with a custom written code in Fortran 90 (the S1 Source Code is included in the supplementary materials). The stochastic equation are integrated through a Euler method [33, 34], while the vectors *ξ*_t_ and *ξ*_r_ (representing the uncorrelated white noise) are three-dimensional vectors of Gaussian random numbers [33, 35], numerically obtained by a Box Muller method [34].

### Simulation of magneto-aerotactic band formation

To compare our simulations to the capillary experiments performed for *M. gryphiswaldense* (see below), the model is extended to include interactions with the capillary walls and a dynamic oxygen gradient: Upon contact with a capillary wall, an active reversal of the direction of motion is simulated, as observed experimentally for flat surfaces [36]. The dimensions of the capillary are matched to the experimental ones as far as possible, with the same length of 40 mm, and a section of 0.1 × 0.1 mm^2^ (real capillary: 40 mm × 2 mm × 0.2 mm), with a average bacterial density as in the experiment (25000 bacteria are simulated, 6.25 × 10^7^ cell mL^−1^ corresponding to an optical density OD = 0.18).

The oxygen gradient builds up dynamically due to diffusion into the capillary from the capillary’s open end and consumption of oxygen by the bacteria in its interior. Therefore, alongside with the integration of the bacterial equation of motion, the equation for oxygen is integrated in three dimensions, as well [12–14]:

$$\frac{\partial C(x,y,z,t)}{\partial t}=D_{O_{2}}\nabla C\left(x,y,z,t\right)-k\frac{C(x,y,z,t)}{C\left(x,y,z,t\right)+C_{a}}\rho \left(x,y,z,t\right).$$ (3)

Here *C* is the oxygen concentration, $D_{O_{2}}$ the oxygen diffusion constant, *k* the consumption rate, *ρ* the local number of bacteria and *C*_*a*_ a cutoff to avoid negative concentration. The boundary conditions on the plane *x* = 0 is set to *C* = 216 *μM*. The starting condition is completely anoxic (the oxygen is absent), as in the experiments. The density of bacteria and the oxygen concentration and gradient are constant within bins of the discretized space (dimension 20 *μ*m × 20 *μ*m × 20 *μ*m).

Since our simulation indicate that robust formation of a band requires a sufficiently steep response to the chemical gradient, i.e. a small value of ∇*C*_0_ in Eq 2 (see S3a–S3d Fig) and the value of ∇*C*_0_ has only a small effect on the chemotactic velocity (S3e and S3f Fig), we used the limiting case of *τ*_run_ for ∇*C*_0_ ≈ 0 in these simulations. Finally, the run times change along the course of a bacterial trajectory, because the run times (or the reversal rates) are dependent on the oxygen gradient as well as the oxygen concentration, the latter because in (magneto-)aerotaxis, oxygen acts as an attractant at low concentrations but as a repellent at high concentrations (see ref. [12]). In our simulations, the duration of a run is decided by the local oxygen concentration and oxygen gradient at the beginning of a run, thus changes within a run are neglected. This is unproblematic, because the oxygen profile changes slowly on the length scale of a run. There is however, one exception: In our simulations of magneto-aerotactic band formation, when a bacterium crosses the preferred oxygen concentration, the run times change drastically because of the reversed bias as oxygen switches from an attractant to a repellent. Therefore, in this situation the change of the run time during the run has to be included. This is implemented by starting a new run in the same direction when crossing the preferred concentration (implementing an instantaneous change in the reversal rate). Without this, unrealistically broad bands form (see S4 Fig), as the bacteria ‘overshoot’ in the unfavorable direction, while the change in the reversal rate results in shorter runs, reducing band width.

### Capillary experiments

The experiments are performed following Bennet et al. [12, 13]. Briefly, MSR-1 bacteria cultures are grown in the cultivation medium reported by Heyen and Schüler [37]. A motile population is obtained using a procedure reported by Bennet et al. [13]. After growing in the agar semisolid medium, they are grown to mid-exponential phase in homogeneous oxygen conditions in the tubes at 1 mT homogenous magnetic field. 1 ml of motile cells is harvested during the cell’s mid-exponential growth phase. After harvesting, the cells show axial magnetotactic behavior, meaning that under homogeneous oxygen conditions they swim in both directions in a strong external magnetic field. An optical density (OD) of 0.1 at 565 nm is adjusted, which corresponds to 3.4 × 10^7^ cells ml^−1^. Two other OD are considered: 0.05 and 0.2. The sample is degassed using nitrogen for 15 minutes and the sample is introduced into a rectangular micro-capillary (VitroTubes, #3520–050,) by capillary forces. One end of the capillary is sealed with petroleum jelly and the capillary is glued onto a microscope slide and mounted onto the microscope stage. The microscope features 3 orthogonal Helmholtz coils, which are used to cancel Earths magnetic field with a precision of 0.2 *μ*T and to create a homogeneous 50 *μ*T magnetic field antiparallel to the oxygen gradient inside the capillary at the sample position [13]. A 60x objective (Nikon, Plan Apo VC, 1.2 NA, WI) is used to image cell motility and localize the band position. A 10x objective is used to image the band and measure its size. The images are processed with ImageJ (Version 1.50h, Wayne Rasband National Institutes of Health, USA): from 100 frames (fps = 100 s^−1^) the standard deviation is calculated. The standard deviation image is a quantification of the movement of the bacteria, where white areas indicate high movement and black areas low movement. The gray-intensity profile of the standard deviation image is then plotted as function of the position in the frame, with the vertical values averaged, with ImageJ ‘Plot Profile’ function. in this way, the band profile is obtained and then it is fitted with Matlab (Version R2017a, Mathworks) with a Laplace distribution. The 10x magnification videos are pre-processed with ImageJ for background subtraction and quality improvement, and then they are tracked with a custom-made tracking algorithm [13]. The 4th-order velocities are extracted from the trajectories with a custom-made Matlab program.

## Results

### Run and tumble chemotaxis in an external field

To study the interplay of chemotaxis and external fields guiding the swimming of a microorganism, we introduced a model that combines the features of an active Brownian particle, specifically a Langevin equation, into which external forces and torques are easily introduced, with mechanisms for active directional changes such as tumbling and reversals (see Methods and Fig 1a). The latter are modulated by chemical gradients in order to implement a coarse-grained description of chemotaxis that does not explicitly describe the underlying signaling. Example trajectories for run and tumble in the presence of an external magnetic field without chemical gradients are shown in Fig 1b. The magnetic interaction helps to reduce the motion of the bacteria to an effectively one-dimensional motion and directs it in the direction of the magnetic field.

**Fig 2:**
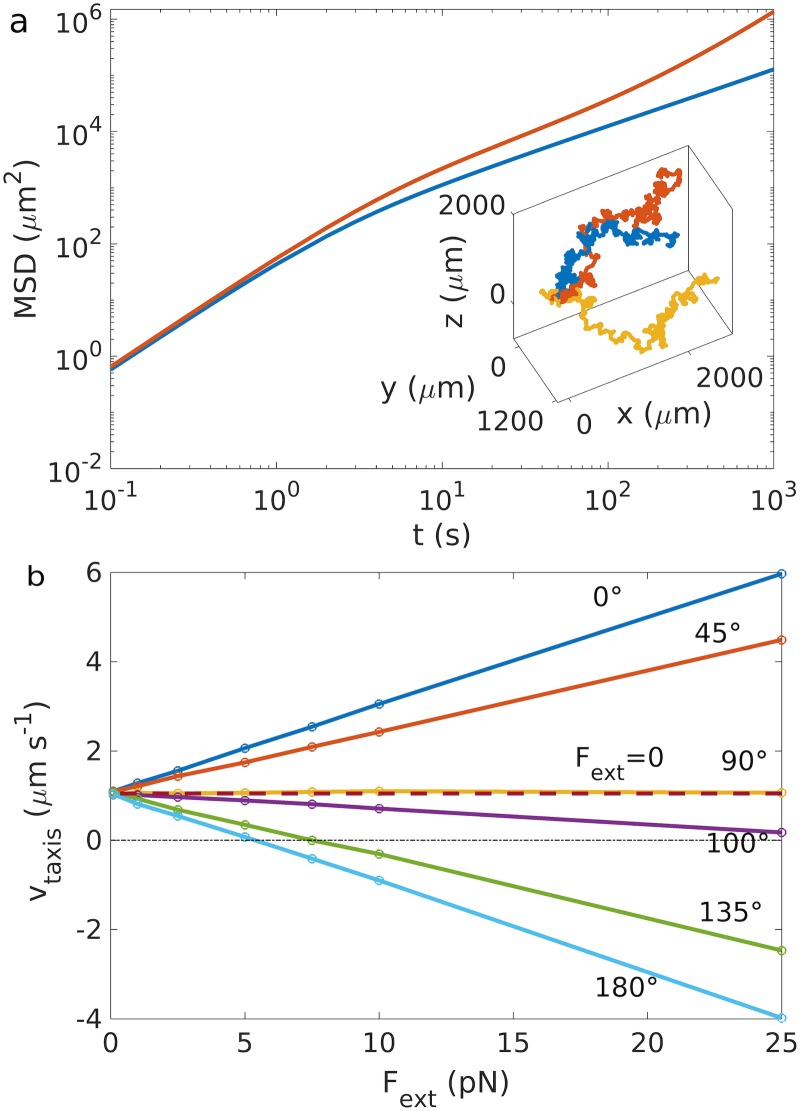
Chemotaxis without/with forces. a) Comparison between the mean square displacement with (red) and without chemotaxis (blue). Inset: three different realizations of trajectories with chemotaxis towards an attractant with gradient directed along $+\hat{x}$. b) Taxis velocities in the presence of a constant force at different angles *θ*_F,∇C_ relative to the concentration gradient and with different absolute values *F*_ext_. The dashed line corresponds to the case without force. For the used parameters, see S1 Table.

Using this model, we first simulate run and tumble chemotaxis. Fig 2a shows representative trajectories and the one-dimensional mean square displacement. In the absence of a chemoattractant gradient, a bacterium performs a persistent random walk with directed motion with velocity *v* on small time scales and random diffusive motion on long time scales. The latter is characterized by the effective three-dimensional diffusion coefficient $D_{\text{eff}}≃\frac{v^{2}\tau_{\text{run}}^{2}}{3\alpha (\tau_{\text{run}}+\tau_{\text{tumble}})}$, with *α* = (1 − 〈cos *θ*_tumble_〉) [38]. A gradient of a chemoattractant biases this random walk up the gradient with velocity *v*_taxis_ ≃ 1.08 *μ*ms^−1^ (Fig 2a). As a first simple example for the influence of an external field, we consider a constant external force, which might represent a force applied by the flow of the fluid in which the swimmer moves, or by optical or magnetic tweezers onto the swimmer [39]. Under a constant force, the run and tumble trajectories become biased (stretched out) in the direction of the force. The directionalities imposed by the force and by chemotaxis are linearly superimposed, thus forces with a component parallel to the gradient enhance/promote chemotaxis, while forces with an antiparallel component reduce it (Fig 2b). Chemotactic swimming up the gradient is impossible for opposing forces exceeding a threshold value F* = −*γ*_t_ *v*_taxis_(*F* = 0)/cos *θ*_F,∇C_, where *θ*_F,∇C_ is the angle between the gradient and the force (see S2 Text for the calculation of the taxis velocities). We note that the dependence on the force may be more complex than linear if chemotactic signaling is in a recently described nonlinear regime, where the change of the concentration during a run causes a positive feedback in the signaling system [40].

### Run and tumble under the influence of a magnetic torque

**Fig 3:**
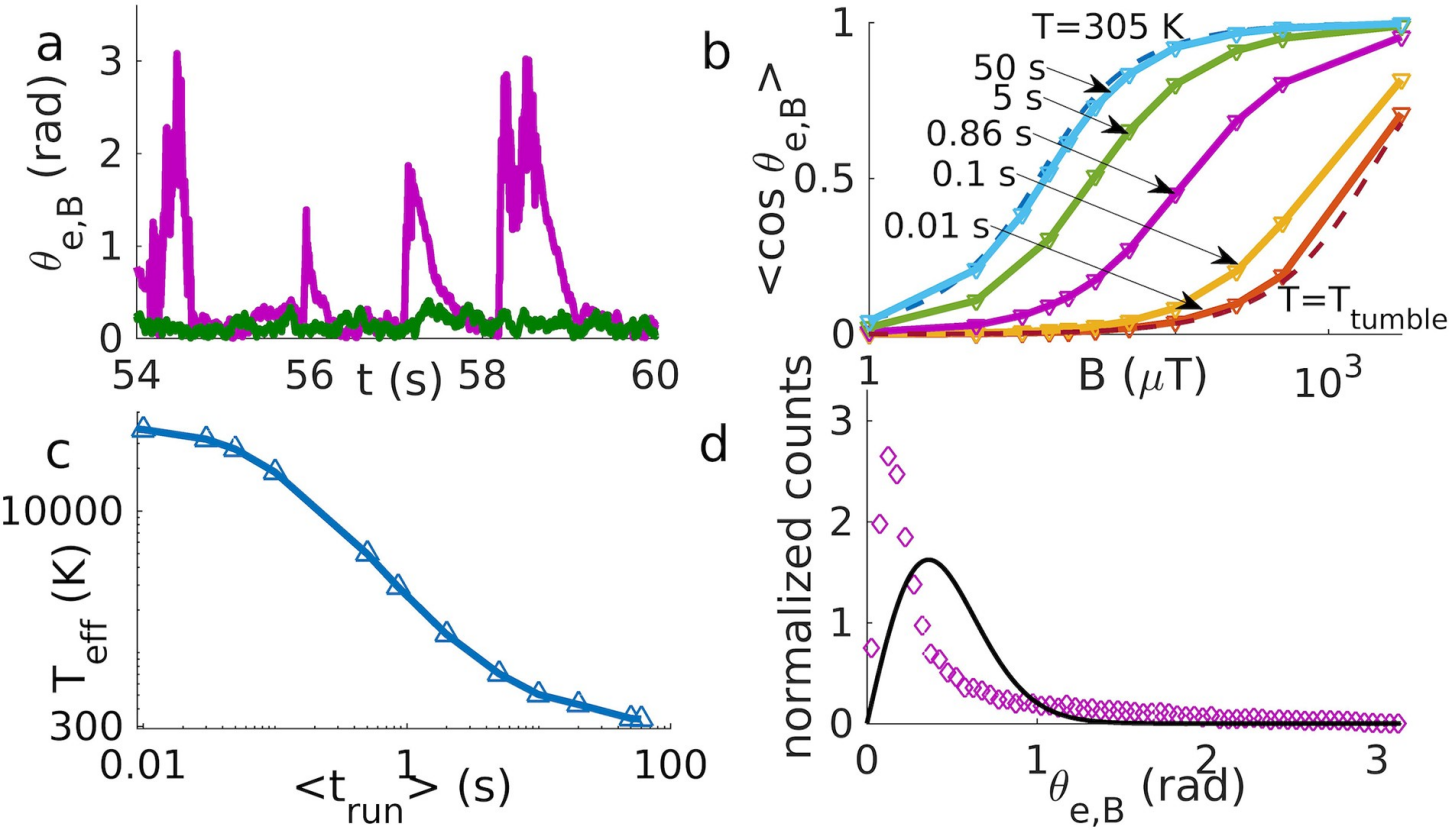
Motion with magnetic torque. a) Alignment angle vs. time for run and tumble (in purple) and run and reverse (in green) for a mean run time of 0.86 s at 500 *μ*T. b) Langevin plots for run and tumble compared to theoretical Langevin functions at *T* and *T*_tumble_ (dashed lines). Each line corresponds to a different mean run time. c) The effective temperature obtained by the Langevin fit in b) is plotted against the mean run time. d) The distribution of the alignment angle for run and tumble with a mean run time of 0.86 s at 500 *μ*T (data points) deviates from the thermal distribution with the corresponding effective temperature, 2896K (solid line).

We next use the model to describe the interaction of a bacterium with a magnetic moment (which could be a magnetotactic bacterium or a magnetically functionalized *E. coli*) with a homogeneous magnetic field **B** in the absence of chemical gradients. In this case, we have a torque **T**_ext_ = **M** × **B**, where **M** = *M* **e** is the magnetic moment of the bacterium, but no force, **F**_ext_ = 0. The magnetic torque tends to align the direction vector **e** of the bacterium with the magnetic field and thereby biases the motion in the direction of the field. However, tumbling perturbs this alignment, so run and tumble motion is characterized by strong perturbations of alignment due to tumbles, followed by relaxation to the aligned state during the runs (Fig 3a) (with a characteristic relaxation time $\tilde{\tau }={\left(B\gamma_{\text{r}}^{-1}M\right)}^{-1}$ [11, 41], see S3 Text and S5 Fig for more details). Thus, there is a competition between two time scales, the relaxation time characteristic of alignment $\tilde{\tau }$, and the mean run time *τ*_run_, after which a tumble perturbs the alignment. For $\tau_{\text{run}}<\tilde{\tau }$, the magnetic field does not have enough time to re-align the bacterium before the next tumble, resulting in larger fluctuations similar to an increased temperature. Indeed, the alignment of the swimming direction of magnetotactic bacteria has been described by an effective temperature [42, 43]. To test this concept in our model, we fit the cosine of the angle between the field and the orientation vector *θ*_*e*,*B*_ with the Langevin function [11, 42] (see also S4 Text and S6 Fig)

$$\left\langle \text{cos}\left(\theta_{e,B}\right)\right\rangle =\text{coth}\left(MB/k_{B}T_{\text{eff}}\right)-{\left(MB/k_{B}T_{\text{eff}}\right)}^{-1}$$ (4)

using the effective temperature *T*_eff_ as a fit parameter (Fig 3b). The effective temperature depends on the run time (Fig 3c), reflecting the competition of the two time scales, and interpolates between the noise strength of tumbling and the actual temperature. While the Langevin function with an effective temperature gives a good fit for the order parameter 〈cos(*θ*)〉, the distribution of the angle deviates clearly from a thermal distribution [11] (see also S4 Text)

$$P\left(\theta \right)=\frac{MB}{k_{\text{B}}T_{\text{eff}}}{\left(2\text{sinh}(\frac{MB}{k_{\text{B}}T_{\text{eff}}})\right)}^{-1}\text{sin}\theta \text{exp}\left(\frac{MB}{k_{\text{B}}T_{\text{eff}}}\text{cos}\theta \right)$$ (5)

with that effective temperature (Fig 3d). The simulated distribution presents a peak due to the thermal distribution during runs after relaxation and a broad tail that depends on tumbling and relaxation and is not explainable with a thermal distribution. This observation suggests that measuring the distribution of the alignment angle might provide a way to distinguish the non-thermal noise due to discrete tumble events from non-thermal noise that might be present continuously due to the active swimming motion. The observation of a peak at low angles is consistent with a recent report, where this peak is interpreted as a velocity condensation effect [44]; however, our peak is not centered around zero as in that report, but has its maximum at a finite value. We show in S7 Fig that this difference arises from the presence of noise and from considering trajectories in a three-dimensional space rather than two-dimensional projections, both in contrast to ref. [44]. Finally we note that the non-thermal fluctuations that are induced at discrete time points by tumbling are not observed in the run and reverse scenario, where the fluctuations of orientation are thermal in all states of the particle.

### Chemotaxis with magnetic torque

The magnetoaerotaxis of magnetotatic bacteria provides an example of chemotaxis influenced by a magnetic torque. The magnetotatic bacteria perform aerotaxis, a chemotactic motion towards micro-aerobic conditions, i.e. towards a preferred (low) oxygen concentration, while being passively oriented by the magnetic field of the Earth. In the natural environment, the inclination of the magnetic field of the Earth with respect to the vertical direction typically results in an angle between the directions defined by the magnetic field and the oxygen gradient. Here we test this scenario with our model for a gradient constant in time and space.

**Fig 4:**
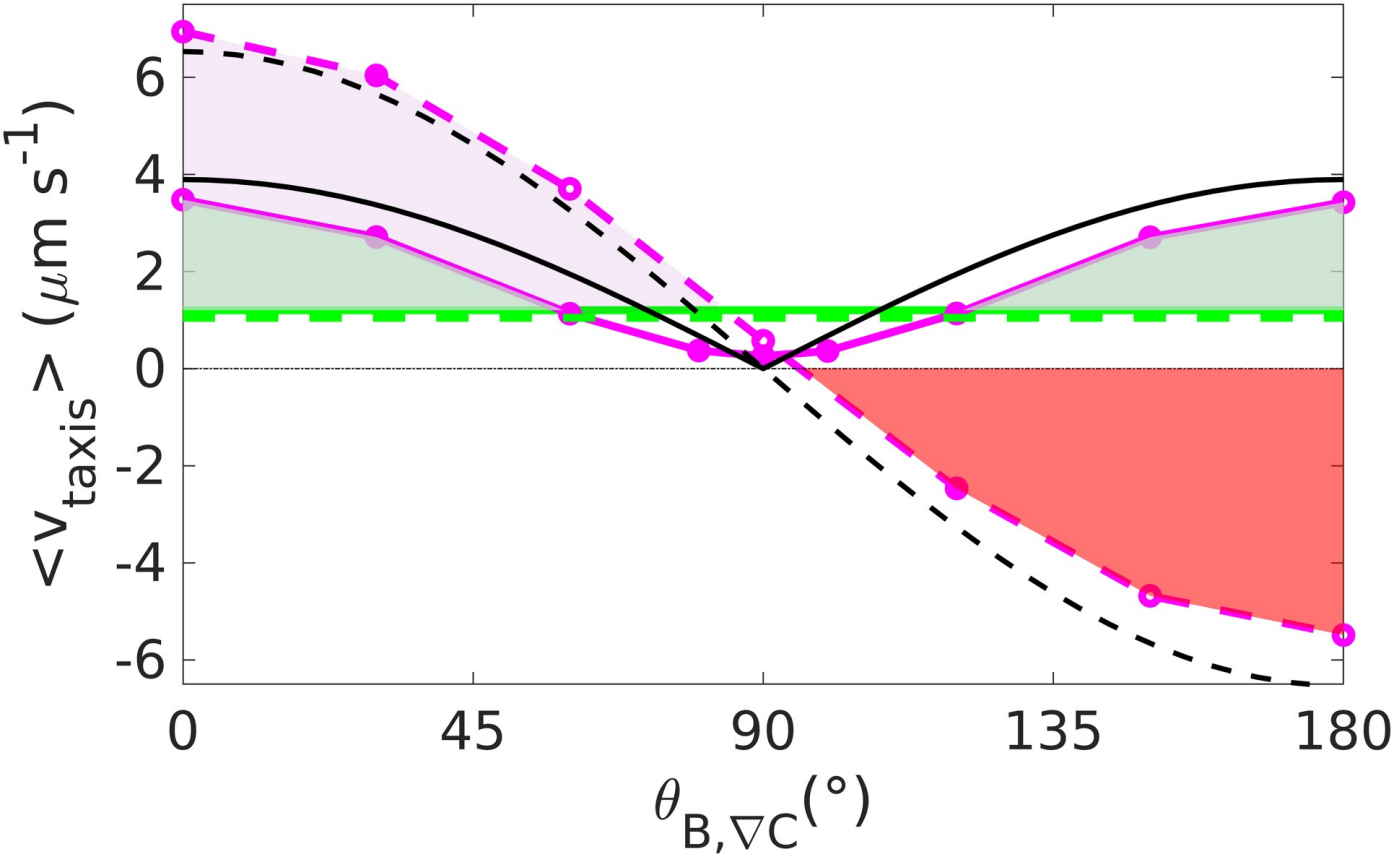
Chemotaxis with magnetic torque. Taxis velocity up the gradient for run and reverse (solid lines) and run and tumble (dotted lines) at *B* = 0 *μ*T (green line) and *B* = 50 *μ*T (purple line). In black, the theoretical predictions. In the presence of a field at a small angle *θ*_*B*,∇*C*_ relative to the concentration gradient, run and tumble performs better than run and reverse (the pink area shows the gap between the tumble curve and the reverse curve). Reverse plus magnetic field outperforms reverse without the field except for angles around 90° (the green area shows the gap between the curves with and without field for reverse). Tumble with field, on the other hand, hinders chemotaxis at large angles (the red area shows the gap between zero velocity and the tumble curve).

In particular, we compare different taxis strategies in the presence of magnetic fields (reverses appear typical in magnetotactic bacteria [13], but there have also been reports of tumbling [45]), with the aim of explaining why natural magnetotactic bacteria adopt some strategies and not others. To this extent, we investigate the effect of a magnetic field on chemotaxis towards an attractant for run and tumble as well as run and reverse motion. Our simulations show that a magnetic field parallel to the gradient is beneficial to both scenarios, since it increases the chemotactic velocity, but that an antiparallel field can be overcome only by run and reverse (Fig 4), suggesting that the natural behavior of magnetotactic bacteria is dominated by reversals rather than by tumbling, consistently with experimental observations [13, 45]. The price for the ability to swim against the direction of the magnetic field is an overall lower velocity (compared to tumble) due to the greater contributions of backward motion. Nevertheless, the magnetic field enhances the chemotactic motion up to rather large angles (approximately 60° in Fig 4). For angles close to 90°, the chemotactic velocity is lower than without the field for both reverse and tumble, therefore rising the question whether magnetotaxis is beneficial at such high field inclinations.

An analytical expression that approximately predicts the taxis velocity as function of the magnetic field intensity and orientation can be obtained in the following way: We first consider the case of run and tumble. Neglecting noise, the bacteria are forced to swim in the direction of the magnetic field. Thus, their motion up the gradient is given by the projection of their velocity in the direction of the field, which is given by the cosine of the angle between the magnetic field and the gradient, in the direction of the gradient, cos *θ*_e,B_. The projection effect is seen directly by the collapse of the velocity data for different magnetic field shown in S8 Fig. The velocity along the field, however, is not the swimming velocity *v*, but reduced by orientation fluctuations, which include both thermal noise and the those arising form tumbles and subsequent relaxation. The average alignment and thus the average velocity in the direction of the field can be described by an effective temperature, as shown above (Fig 3c). Combining these two consideration, we arrive at the following expression for the chemotactic velocity,

$$v_{\text{taxis}}^{\text{tumble}}≃v\text{cos}\left(\theta_{\text{B},\nabla \text{C}}\right)(\text{coth}(\frac{MB}{k_{\text{B}}T_{\text{eff}}})-{\left(\frac{MB}{k_{\text{B}}T_{\text{eff}}}\right)}^{-1}).$$ (6)

For run and reverse, these considerations have to be modified, because the bacteria may swim both up and down the gradient. Thus, the cosine of the projection is replaced by its absolute value and the direction is included explicitly by a factor $R\equiv \frac{t_{\text{up}}-t_{\text{down}}}{t_{\text{up}}+t_{\text{down}}+2t_{\text{pause}}}$ that accounts for the fraction of time of up-gradient swimming minus the corresponding fraction for down-gradient swimming (*R* ≃ 0.31 for our parameters). The full expression for the taxis velocity up a constant gradient for run and reverse is thus

$$v_{\text{taxis}}^{\text{reverse}}≃v|\text{cos}(\theta_{\text{B},\nabla \text{C}})|(\text{coth}(\frac{MB}{k_{\text{B}}T})-{\left(\frac{MB}{k_{\text{B}}T}\right)}^{-1})\frac{t_{\text{up}}-t_{\text{down}}}{t_{\text{up}}+t_{\text{down}}+2t_{\text{pause}}}.$$ (7)

The predictions obtained from Eqs (6) and (7) are included in Fig 4 and seen to provide a good approximation to the simulation result. The agreement gets even better for stronger fields, where the role of noise is minor (S8 Fig).

Our comparison between different strategies for changes of the swimming direction, which identifies reversals as crucial for scenarios with a magnetic field antiparallel to a chemical gradient, might be dependent on choices in our modeling that therefore deserve additional investigation. In our model, we have assumed that runs or reverse runs down the gradient have the same duration as runs in the absence of a gradient, which need not be the case [46, 47]. Therefore we tested whether shortening runs down the gradient changes our results. The corresponding simulations are shown in S9 Fig and show that the advantage of tumbling over reversals in the case of a parallel field is also seen with shortened runs down the gradient, but is less pronounced than without the shortening.

Moreover, in the run and tumble motion of peritrichously flagellated bacteria such as *E. coli* and *B. subtilis*, the tumbles effectively include a mechanism for the reversal of the direction of motion, as the flagellar bundle opens during tumbles and can subsequently form on either side of the cell body, as demonstrated for the swimming of *B. subtilis* in a liquid crystal [48]. We therefore tested how choosing the direction of motion randomly as either parallel or antiparallel to the body orientation after a tumble affects the motion. In that case, motion up the gradient under an antiparallel magnetic field is indeed possible, but slower than in the parallel case and slower than in the absence of the field (S10a Fig). Moreover, such a high probability of changing the direction of motion relative to the orientation of the cell body is not realistic given the distributions of reorientation angles during tumbles cf. S2 Fig, as it would result in a distribution that is symmetric around 90°. To test the influence of the fraction of reversals, we also simulated a combination of reversals and tumbles that interpolates smoothly between the two types of behaviors. We varied the fraction of reversals and found that swimming up the gradient against a magnetic field requires more than 50% reversals (S10b Fig), strengthening the point that a mechanism of reversal is needed for effective motion in the scenario of a gradient opposed by a magnetic field. We also note that to our knowledge, none of the magnetotactic bacteria that were described so far are peritrichously flagellated. If tumbling occurs, as reported in some cases [13, 45], the mechanism for tumbling is likely different from the one in *E. coli*.

Finally, the already mentioned nonlinear regime of the chemotactic signaling system [40] results in a scenario where runs up the gradient are very long and cells spend almost all time running. One might expect that in this case, the difference between the tumbling and reversal strategies disappears. This is indeed true if the magnetic field is parallel to the gradient. However, if the field and the gradient are antiparallel, reversals are still seen to be more efficient than tumbles (S11 Fig). In that case, the tumbling bacteria cannot make full use of the very long runs up the gradient, because immediately after a tumble, the magnetic torque reorients them to align them with the magnetic field and thereby forces them to swim down the gradient with very short runs; in this way, they are never able to access the long runs up the gradient.

**Fig 5:**
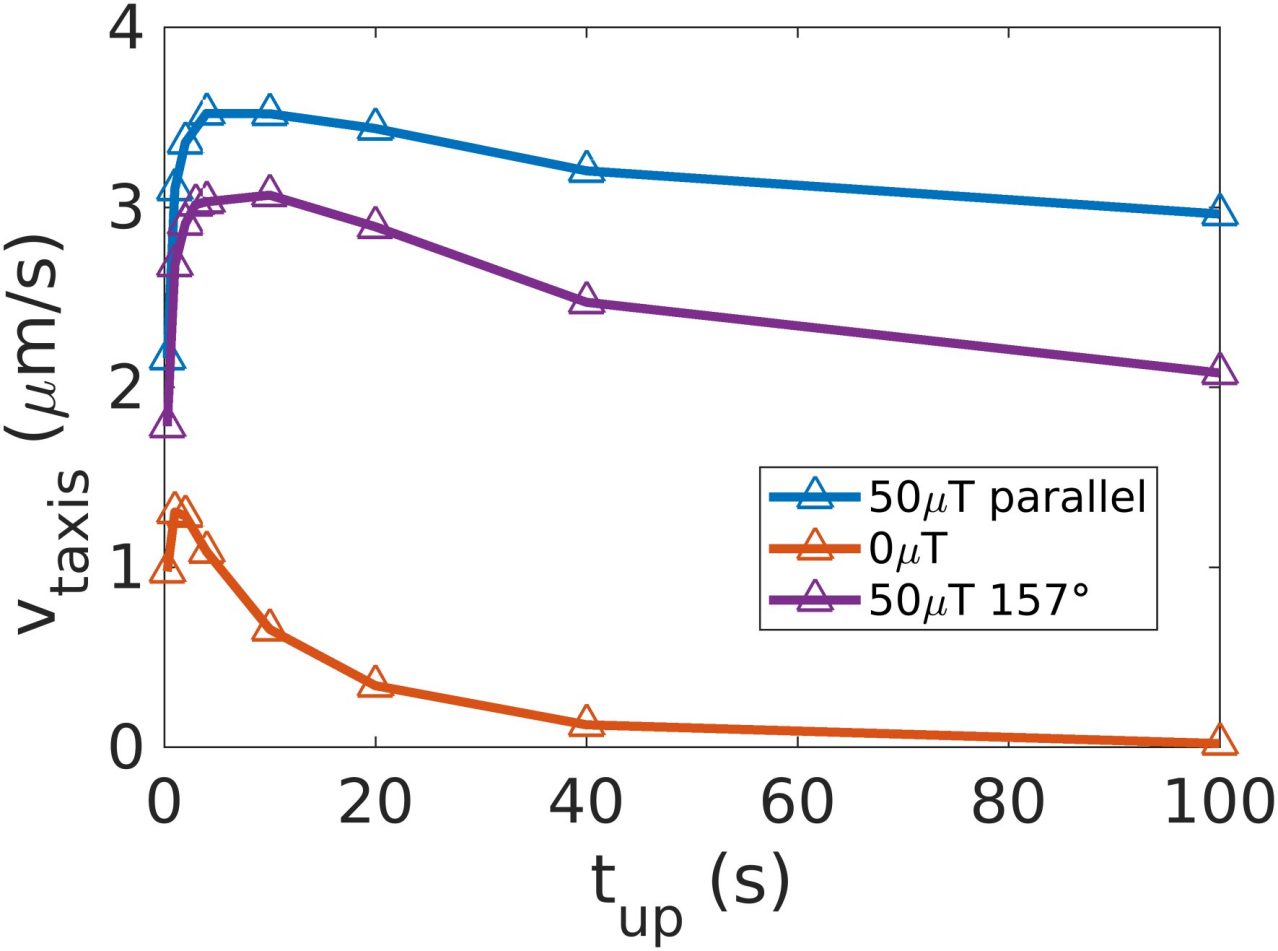
Influence of the mean run time. For run-and-reverse chemotaxis towards an attractant, we study the influence of the mean run time, during which the bacterium swims up the gradient, on the taxis velocity. We notice that for *B* = 0, a peak is present around 2 s. For *B* = 50 *μT* (parallel to the gradient or at an inclination of 157° (inclination of the Earth magnetic field in Berlin to a downward gradient in a stratified aqueous environment), a plateau is reached instead.

For the case of run and reverse chemotaxis towards an attractant, we quantify how the mean run time influences the taxis velocity with and without the magnetic field. Magnetotactic bacteria can perform very long runs compared to the runs of *E. coli* [32]. Without a magnetic field, very long runs would be detrimental for gradient sensing, since thermal noise results in a re-orientation of the cell within a time *t* given by < cos^2^ θ >= 6*D*_r_*t*. Without a magnetic field (*B* = 0), we indeed see that the maximal taxis velocity is reached for a run time of about 2s (red curve of Fig 5), for which the average thermal reorientation is ≃ 50°. When a weak magnetic field of 50 *μT* is turned on (blue curve of Fig 5), the taxis velocity is higher than without the field, as shown in the previous sections. Moreover, the velocity reaches a plateau for run times exceeding 2 s, showing how long runs benefit from the presence of magnetic fields. The advantage persists for the inclination of the magnetic field of the Earth relative to a vertical gradient in a stratified aqueous environment (with an angle of 157° as in Berlin [49], see the purple line in Fig 5).

### Magnetoaerotactic band formation with a constant gradient

**Fig 6:**
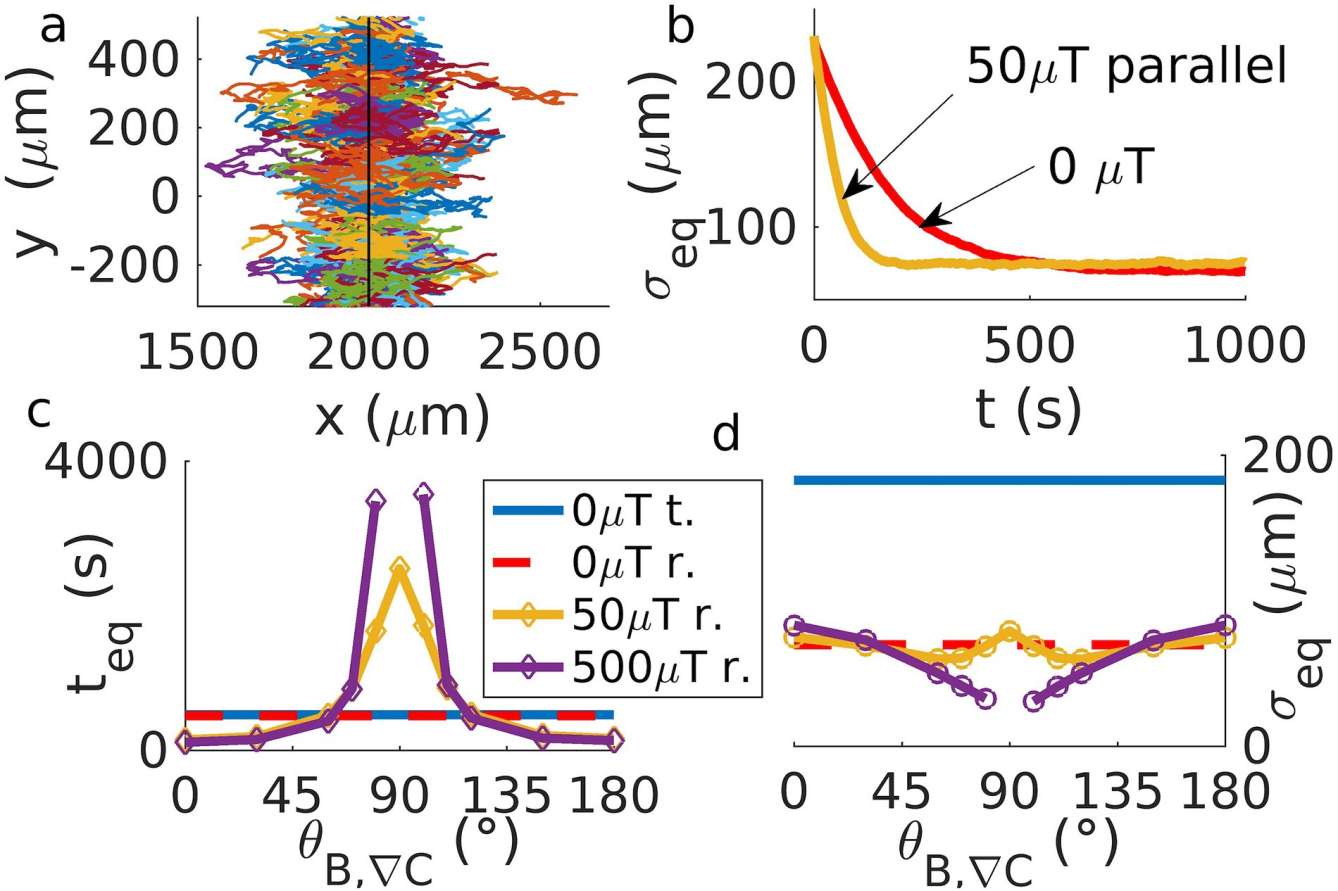
Band formation in constant concentration gradients. a) 100 example trajectories are shown for chemotaxis towards a preferred concentration at the position indicated by the black line. The bacteria concentrate there and form a band. b) Width of the band as a function of time, estimated by the standard deviation of the position of the bacteria in the direction of the gradient, *σ*_eq_, for scenarios with and without a magnetic field parallel to the gradient. c) Equilibration time *t*_eq_ (*i.e*. time at which the band stop growing in amplitude and reaches steady state) and d) width of the band at equilibrium, plotted as a function of the angle *θ*_*B*,∇*C*_ between the gradient and the magnetic field, for tumble (t.) and reverse (r.) with different intensities of the magnetic field. While run-and-reverse behavior results in band formation for almost any magnetic condition, run-and-tumble bacteria only forma a band without magnetic fields.

In the presence of an oxygen gradient and of a magnetic field, magnetotactic bacteria accumulate in regions of their preferred oxygen concentration C*, which leads to the formation of a band of high bacterial density in a quasi-one dimensional geometry such as a capillary [12–14]. This behavior is recovered in our model, when we treat oxygen as a chemoattractant at concentrations *C* < C* and as a chemorepellent for *C* > C* in the run and reverse scenario (see S1 Text) (Fig 6a). In the run and tumble scenario, formation of a band is only seen in the absence of a magnetic field, as band formation in the presence of a magnetic field requires motion against that field, which is prohibitively unlikely in run and tumble, as discussed above. However, as aerotaxis without a magnetic field would be sufficient for band formation (and indeed also occurs in non-magnetic bacteria [50]), the question arises what the advantage of magnetically assisted aerotaxis might be. A possible answer is that with magnetic assistance, the band forms more rapidly. Thus, we simulate the formation of a magneto-aerotactic band in a fixed linear oxygen gradient. We estimate the width of the band by the standard deviation *σ*_eq_ of the swimmer position in the direction of the gradient (Fig 6b) (for an alternative derivation, see S12 Fig). This quantity relaxes quickly as the band is formed. The relaxation time of band formation and, to a lesser extent, the steady-state width of the band are seen to depend on the intensity of the magnetic field and the angle of application (Fig 6b–6d). The formation of the band is indeed sped up at low inclination of the field relative to the gradient. For inclinations approaching 90°, however, band formation is strongly slowed down, in particular for high field strength for which it is inhibited, consistent with our observation of a reduced chemotactic velocity above. Notably, for field strengths comparable to the Earth’s magnetic field, a band still forms in less than an hour, indicating that magneto-aerotaxis (based on run and reverse) remains functional even at 90°.

As already discussed above, run and tumble motion of peritrichous bacteria includes a mechanism for reversals by re-forming the flagellar bundle on the opposite side of the cell body. One would therefore expect that this scenario allows for band formation. This is indeed the case as shown in S13 Fig, but the band is wider than for run and reverse. We tested again combinations of tumbling and reversals and found that a large fraction of reversals (>50%, in excess of what is consistent with the reorientation distribution of *E. coli*) is needed for robust band formation. Thus, while not all run and tumble mechanism prohibit band formation, it is the reversals included in an *E.coli*-type tumbling mechanism that are crucial and allow the required motion against the magnetic field.

Together, our observations of band formation support the following picture: a mechanism for swimming in the direction imposed by the magnetic field and well as against that direction is required for magneto-aerotaxis. Such a mechanism is provided by reversals. For reversing bacteria, a magnetic field can speed up the formation of an aerotactic band for low inclinations, but does not prohibit it at inclinations close to 90°, consistent with the presence of magnetotactic bacteria close to the Earth’s equator [51].

### Magnetoaerotactic band formation with a dynamic gradient

**Fig 7:**
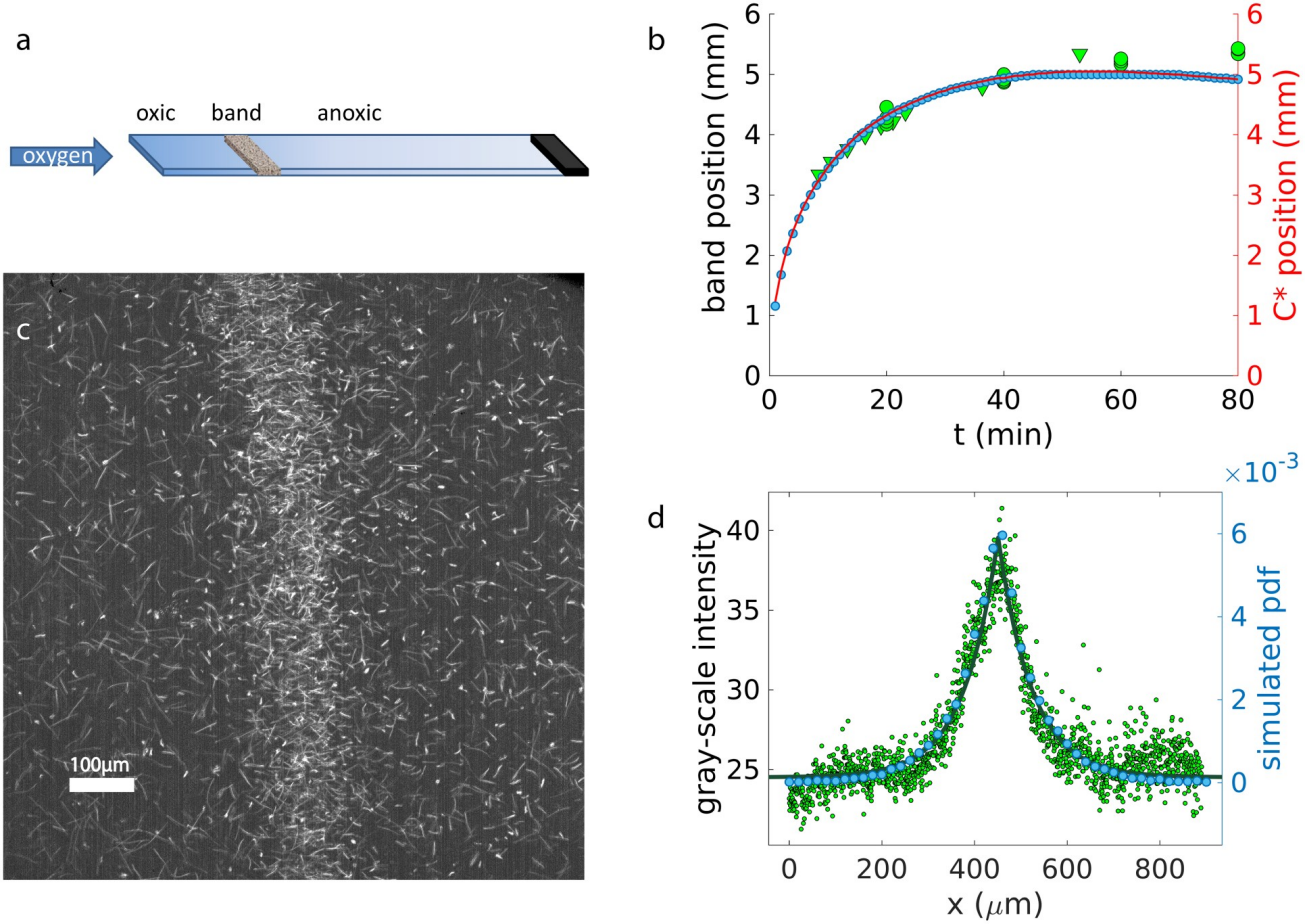
Capillary experiments and simulations. a) Scheme of a capillary experiment: a capillary is filled with bacteria; one end of the capillary is sealed, the other is open to the flow of oxygen. The bacteria accumulate at the preferred oxygen concentration, forming a band. b) Experimental band position over time (green data points) for wild type (WT) axial bacteria (optical density OD = 0.1, 5 repetitions, two different days—triangles and circles), with an antiparallel magnetic field of 50 *μ*T, compared to the simulated band position (blue data points) under the same conditions. The red line represents the position of the preferred concentration position as obtained from the simulation. c) Standard deviation of 100 frames (1 s) of a video of the band recorded at 100 fps and 10x magnification (WT OD = 0.1 antiparallel 50 *μ*T). d) The gray-scale values are averaged along the y coordinate of Fig. c), giving the experimental density profile of the band (green data points). The green line represents the Laplace fit (decay length 73 *μm*). The simulated probability density function (pdf) $n_{\text{bacteria}}^{\text{bin}}/n_{\text{bacteria}}^{tot}$ at equilibrium is represented by the blue data points (decay length 75 *μm*).

To compare our simulations results with experimental data on the magnetically assisted aerotaxis, we perform capillary experiments for the magnetotactic bacterium *Magnetospirillum gryphiswaldense* MSR-1 and follow the formation of the magneto-aerotactic band. The bacteria, which perform axial magnetotaxis, i.e. swim along the field line without a directional preference, are placed in capillary with one open an one closed end. The interior of the capillary is initially anoxic and oxygen diffuses in from the open end, resulting in an oxygen gradient. A homogeneous magnetic field of 50 *μ*T is applied antiparallel to the oxygen gradient. As a magnetoaerotactic band forms, its position is recorded over time (green data points of Fig 7b). Moreover, we record videos of the stationary band from which the band profile can be extracted as described in Methods (green data points in Fig 7b). From this image, the gray-scale intensity is extracted, which is proportional to the bacterial distribution in the band (green data points of Fig 7d). The bacterial distribution can be fitted by a symmetric Laplace distribution (green line), which gives a characteristic size of the band of 73 *μ*m. Moreover, by tracking individual bacteria, their mean velocity is determined to be 50 *μ*ms^−1^. These parameters are used in the following simulations since we now focus on reproducing a specific system.

We compare these experimental data to our simulations to test our aerotactic model. Compared to the simulations above, where a constant gradient is considered, the simulations are extended to include the dynamics of the oxygen gradient (due to oxygen diffusion and consumption by the bacteria) and the interactions of the bacteria with the walls, see Methods. Interactions between bacteria (specifically excluded volume) are neglected, since the system remains rather dilute even within the band. The run times in the favored and unfavored directions, and the oxygen consumption rate *k* are used as free fitting parameters. We vary these parameters to match the stationary band size and the position of the band over time. In general, reducing the difference between the two run times and reducing the consumption constant make the dynamics slower; the band size decreases with larger differences between the run times; and the oxygen consumption rate has a strong effect on the stationary position of the band (see S14 Fig S14 for more details on the influence of the parameters on the final outcome). The best match in Fig 7 is obtained with run times of 2 s and 0.9 s and *k* = 0.005 mol min^−1^ cell^−1^. The run times are comparable with the ones of *E. coli* [28], while they seem to underestimate the mean values for polar MSR1 [32], even though further experiments are needed to measure the biased run times up and down the gradient for magnetotactic bacteria.

As it can be seen from Fig 7b and 7d, the simulated data (blue data points) show the same behavior as the experiments (green data points). The band forms close to the open end and moves further into the capillary reaching a stationary position such that the consumption of oxygen (mostly) in the band balances the diffusive flow of oxygen to the band. The density profile of the band is compatible between simulations and experiments, with a band width of 75 *μ*m (Fig 7d). Likewise, the band position is well reproduced by the simulation, at least up to 40 min (data points in Fig 7b); small deviations between the two at larger times likely reflect the fact that the number of bacteria in the band keeps increasing slowly in the simulations due to bacteria arriving from the very far anoxic end of the capillary, while in the experiments many of these bacteria are likely inactive or dead and never reach the band. In the simulation, the band position tracks the location of the preferred oxygen concentration (indicated by the red line), indicating the aerotaxis of the simulated bacteria. Thus, our magneto-aerotactic model gives an effective representation of the system. More simulation at different magnetic fields intensities are show in S15 Fig, which confirm the results obtained in the constant gradient simulations where the band formation is speed up by the magnetic interaction.

## Discussion

In this paper, we propose an effective model for bacterial motility that couples chemotaxis, swimming strategies and external forces and torques. We show that the model reproduces the experimental data for magnetotactic bacteria, and therefore can be used to explore the effect of external forces and torques on the chemotactic behavior of microswimmers, in particular highlighting the effects on the chemotactic behavior of bacteria and biohybrids. We show that forces improve chemotaxis for angles smaller than 90° and totally hinder it for values higher than a threshold value (Fig 2b), for both run and reverse and run and tumble motions. This could be of particular interest when considering bacteria or biohybrids actuated and directed by external forces, such as magnetic swimmers in a magnetic gradient [17] and under the influence of magnetic tweezers [39], or when considering chemotactic swimmers inside a fluid flow [36, 52], for which previous studies ignored the complexity given by chemotactic behaviors.

While the effect of the forces does not depend on the strategy of motion of the bacteria, we show that bacteria performing ‘run and reverse’ or bacteria performing ‘run and tumble’ behave differently in the presence of magnetic torques. In particular, even though bacteria performing tumbles have an advantage compared to bacteria performing reversals under a parallel magnetic field, the presence of an antiparallel field completely inhibits chemotaxis in the case of tumbling, and thus tumbling bacteria cannot form a chemotactic/ aerotactic band in the presence of a magnetic field. If the mechanism of tumbling, as the opening and reformation of a flagellar bundle in peritrichously flagellated bacteria, includes a mechanism of reversals (by forming the bundle on the opposite pole of the cell [48], tumbling allows for band formation, but less efficiently and only if such reversals are more probable than what is consistent with observed reorientation distributions. Moreover, it is not clear whether this applies to magnetotactic bacteria, as no peritrichous magnetotactic bacteria are known.

On the one hand, this observation could explain why magnetotactic bacteria do not rely on tumbles as the main mechanism to actively change direction and instead use reversals, or 90° changes, or mixed behaviors [31, 32, 45, 53]); on the other hand, this observation provides a crucial design constraint in planning future biomedical applications with biohybrids, magnetically functionalized, but naturally non-magnetotactic organisms. Until now, the swimming and taxis strategies have not been taken into account in detail and the design of such applications only considered the general chemotactic characteristic of the species [16]. Our results indicate that a mechanism for the reversal of motion is needed for chemotaxis in the presence of magnetic guidance, so suitable non-magnetic bacteria must be used; in laternative, flicks with mean angles of 90° could also be suitable (see MC-1 behavior [53]). If reversals due to tumbling with formation of the flagellar bundle on the opposite side of the cell body turn out to be sufficient, the biohybrid design must ensure that this remains possible when the cells are attached to large beads [16] or partly encapsulated [54].

The crucial difference between the two chemotactic strategies is one of directionality more than a difference in the underlying signaling. For both run and reverse and run and tumble, a magnetic field reduces the aerotactic search from a three-dimensional search to one-dimensional one, confined (up to fluctuations in alignment) along the direction of the field. For tumbling bacteria, directed swimming occurs only in the direction of the field, while reversing bacteria can swim in both directions and are thus able to perform one-dimensional chemotactic motion even against the field direction. On top of this directionality effect, there could be more detailed interactions between the chemotactic response and external fields that are mediated by the signaling apparatus. For example, it was shown for *E. coli* that the swimming response to a chemical gradient results in a behavioral feedback because the signal received by the cell is affected by the cell’s motion [40, 47]. Since the latter motion is modified by the external field, one can expect that under strong gradients the field will impact the chemotactic response also in more subtle ways mediated by the signaling system. A related question is to what extent the impact of the external field is dependent on how close the system is to optimal parameters for the given signaling system, as the chemotactic performance is rather sensitive to the phenotypic parameters [47, 55]. Unfortunately, for magnetotatic bacteria, the chemotactic signaling mechanisms are not very well understood, as only few studies have addressed the underlying molecular signaling system [32]. At a coarse-grained level, however, the rates of directional changes (tumbles or reversals) have to depend on both the local oxygen concentration and the local oxygen gradient [12]. The dependence on the oxygen gradient determines the direction of motion (although a recent theoretical study has proposed a mechanism where a dependence on the local oxygen concentration may be sufficient [56]), while a dependence on the oxygen concentration is needed to switch between oxygen as an attractant and a repellent.

Our work can also throw some light on why magnetotactic bacteria produce the intracellular magnetic chain: we demonstrate from the microscopic point of view that parallel or antiparallel magnetic fields speed up chemotaxis considerably, even for weak fields such as the Earth’s magnetic field, confirming and extending previous work at the population level [13–15]. We also show that this advantage is strongly dependent on the angle between magnetic fields and chemical gradients: for high angles, chemotaxis is actually slowed down, but not completely hindered, in accordance with the finding that magnetotactic bacteria exist at the Equator [51]. Moreover, we show that magnetic fields allow the bacteria to perform very long runs without losing the orientation, indeed tracking of single bacteria shows that runs can extend up to 20 s [32]. Why these long runs are useful for the bacteria is still an open question. They could be useful to bacteria in the oxic zone of water above the sediment, which try to return rapidly to the preferred microoxic conditions in the sediment. Future studies will have to explore what happens to the bacteria once they swim inside the sediments. Does the magnetic field still provide an advantage in such porous media? Some research has been done towards this end [15, 36], but more studies are needed. This would also be of great importance for future biomedical applications where the environments are usually crowded, complex and porous [18, 36].

In summary, we propose and study a model for chemotactic motility that can incorporate external forces and torques due to, e.g., tweezers, fluid flow or magnetic fields. These scenarios are experimentally accessible and our model may inform the design of such experiments. Specifically, we apply the model to describe magnetotactic bacteria, but other magnetically guided microswimmers such as biohybrids can be treated in a similar fashion. The model demonstrates benefits and disadvantages of the magnetic guidance of aerotaxis or other chemotaxis. In particular, the functionality of a run and tumble mechanism under magnetic guidance is limited, which may be important for the design of biohybrid microswimmers that are often based on run-and-tumble bacteria such as *E. coli* [16]. By contrast, a run and reverse mechanism is beneficial in the presence of a magnetic field at moderate inclinations, speeding up chemotaxis/aerotaxis and remains non-prohibitive at high inclinations of the magnetic field relative to the gradient. Beyond the aspects studied here, our model provides a starting point to address the collective behavior of magnetotactic bacteria and other magnetic microswimmers as well as the interplay of the dynamics of taxis with complex confining geometries.

## Supporting information

S1 Text Implementation of chemotaxis. Implementation of chemotaxis for attractants, reppellents and preferred concentration, available as an attachment.
(PDF)

S2 Text Chemotactic velocity for an attractant. Method to calculate the chemotactic velocity for an attractant, available as an attachment.
(PDF)

S3 Text Alignment time in the presence of magnetic fields. Derivation of the alignment angle in the presence of magnetic fields, available as an attachment.
(PDF)

S4 Text The alignment angle distribution and the mean cosine of the alignment angle. Derivation of the of the alignment angle distribution and the mean cosine of the alignment angle, available as an attachment.
(PDF)

S1 Source Code Available as an attachment. (PDF)

S1 FigChanges of direction.The left column shows a cartoon of the mechanism for the change of direction. The change of direction of bacteria can be performed in different ways. Here in this work we considered tumble (upper row) or reverse (lower row). For example, *E. coli* performs tumbles with a mean angle of 68° [28]. In our model, tumbling is implemented as rotational diffusion with a high noise strength, which is chosen such as to match this mean angle. This matching is described in S2 Fig. Other bacteria perform run and reverse motion. During a reversal, the body of the bacteria does not re-orient but the bacteria just inverts the direction of velocity. The mean angle is close to 180°, but no exactly, because of thermal noise. In our model, reverse is implemented as a pause mimicking the slowing of motion during a reversal, so there is a time window without active propulsion, during which rotational diffusion due to thermal noise can reorient the cells direction of motion. The middle column shows three example trajectories for run and tumble and for run and reverse, in absence of external forces, torques or gradients. Both run and reverse and run and tumble nicely explore the three dimensional space thanks to thermal noise and thanks to the active changes of direction. A magnetic field provides a torque aligning the direction of motion with the direction of the field and thus, when the field is turned on, the observed behavior changes. Some examples trajectories in the presence of the magnetic field are shown in the right column (blue is without field, red for *B* = 50 *μ*T -corresponding to the magnetic field of the Earth-, and yellow for *B* = 500 *μ*T. The field is directed along $+\hat{z}$). For run and tumble, the trajectories are stretched in the direction of the magnetic field and motion is unidirectional parallel to the field, with excursions away from that direction. These are due to the tumbles that kick the trajectories out of alignment, followed by re-alignment during the runs, provided those are long enough. For run and reverse, the trajectories show bidirectional motion parallel and antiparallel to the magnetic field. The bacteria remain aligned with the field and have the same probability of swimming up or down. Changes of the direction only occur via rotational diffusion which is suppressed by the field, effectively confining the trajectories to a one-dimensional persistent random walk along the axis of the field.(TIFF)

S2 Fig Tumbling effective temperature. Tumbling has been implemented as an effective rotational diffusion with enhanced noise. The strength of that noise can formally be described by an effective ‘tumbling temperature’, *T*_tumble_. This parameter is chosen such that the resulting mean tumbling angle matches the measured tumbling angle of 〈*θ*_tumble_〉 = 68° observed for *E. coli* [28]. To do so we generated 50000 tumbling angles from a simulation of our model for different noise strengths and calculated the mean and standard deviation for each noise strength to find a parameter value that matches the experimental angle. The normalized distribution of the tumbling angle obtained with the best fit parameter *T*_tumble_ = 4.2 × 10^4^ K is shown here as black bars, and it is seen to be very close to the experimental distribution of Berg and Brown [28] (blue points), with the same mean and standard deviation (〈*θ*_tumble_〉 = (68 ± 40)° compared to 〈*θ*_tumble_〉 = (68 ± 36)° [28]) and a very similar skewness to the right.
(TIFF)

S3 Fig Dependence of the chemotactic response on the parameter ∇*C*_0_. **Effect in capillaries**. Band at 1 min (blue), 10 min (yellow) and 20 min (light blue) and the corresponding oxygen concentration (filled line, dashed line and dotted line) for a) ∇*C*_0_ = 100 *μ*Mmm^−1^, b)∇*C*_0_ = 1 *μ*Mmm^−1^, c) ∇*C*_0_ = 0.01 *μ*Mmm^−1^, d) Step-function. The black arrow shows the time direction. The inset shows the zoom of the band in d). Here the oxygen concentration is integrated only along the long axis of the capillary and it is considered constant in the cross section of the capillary. The band forms only for values of ∇*C*_0_ close to 0, so for simplicity the response function *τ*_run_ is chosen to be a step function, in accordance with previous works [12, 13]. **Effect of ∇*C*_0_ on the chemotactuc velocity for a constant gradient**. Mean position of 1000 bacteria performing attractive chemotaxis with a constant gradient. The slope of the line gives the chemotactic velocity. The results are obtained in green for 0 magnetic field, and in blue for a parallel magnetic field of 50 *μ*T. Filled lines correspond to ∇*C*_0_ = 0 (equivalent to considering a step-function for *τ*_run_), dashed lines correspond to ∇*C*_0_ = ∇*C*/10, and dotted lines to ∇*C*_0_ = ∇*C*. For both tumble e) and reverse f) and in all magnetic conditions, it can be seen that chosing ∇*C*_0_ = 0 or ∇*C*_0_ = ∇*C*/10 gives the same result, while chosing ∇*C*_0_ = ∇*C* slows down chemotaxis of 15% for tumble and 0 *μ*T, 9% for tumble and 50 *μ*T, 20% for reverse and 0 *μ*T and 8% for reverse and 50 *μ*T. In conclusion, changing the cutoff ∇*C*_0_ (thus changing the linear behavior range in *t*_run_) affects in minimum part the chemotactic velocity. The effect of ∇*C*_0_ for a dynamic gradient in a capillary is explored in the supplementary S3 Fig. Again, changing the gradient cut slows down or speeds up the chemotaxis; the effect is more pronounced for this system.
(TIFF)

S4 Fig Band size in the modified model. Band size as function of time for the models with and without resetting of the run (yellow and blue, respectively) after crossing the preferred concentration. Without resetting the bacterium is allowed to finish its run after crossing the preferred concentration, while with resetting a new run is started (likely with a smaller run time) when the bacterium crosses the preferred concentration and runs in the unfavorable direction (thus shorter). As it can be seen, resetting results in a smaller band size, which is also necessary to match the experimental data. For these simulations, *t*_up_ = 6 s, *t*_down_ = 1 s and *k* = 0.01 fmol min^−1^ cell^−1^.
(TIFF)

S5 FigAlignment time in the presence of magnetic fields.The relaxation of the magnetic alignment after a tumble is described by $cos\left(\theta_{\text{e},\text{B}}\right)=\frac{\text{exp}\left(2t/\tilde{\tau }\right)-c}{\text{exp}\left(2t/\tilde{\tau }\right)+c}$, see S3 Text. As an example, we fit (case a), in red) the cosine of the alignment angle *θ*_e,B_ after a tumble for 500 *μ*t without noise (case a), in blue), the case for which the theory was derived. With the parameters of our simulation, the expected value for $\beta =1/\tilde{\tau }$ is 4.4 *s*^−1^. This value is recovered by fitting the simulation data, giving *β* = 4.1*s*^−1^. The fit has been performed with the function $f\left(x\right)=\frac{e^{2b(x-d)}-\left(1-a\right){\left(1+a\right)}^{-1}}{e^{2b(x-d)}+\left(1-a\right){\left(1+a\right)}^{-1}}$, where *b* evaluates $\beta ={\tilde{\tau }}^{-1}$, *a* evaluates *e*_z0_ and *d* is needed to re-scale the times to 0. The data have been fitted with *f*(*x*) as described in the S3 Text (in red). Then we consider the cosine of the angle *θ*_e,B_ after a tumble event at *B* = 500 *μ*T in the presence of thermal noise (case b), blue curve). The data have been fitted with *g*(*x*) = *f*(*x*) + *g*_0_ (in red). The constant *g*_0_ = 〈*cosθ*_e,B_〉 reflects the nonzero mean value of the cosine in thermal equilibrium. The fit in this case leads to *β* ≃ 4.0 s^−1^, still in good agreement with the theory derived in absence of noise. For a magnetic field of *B* = 50 *μ*T, the strength of the magnetic field of the Earth, the relaxation constant is *β* = 0.44 s^−1^, corresponding to a decay time of $\tilde{\tau }≃2.3\;\text{s}$. However, in that case, the fluctuations around that decay are considerably more pronounced when the thermal noise is present.(TIFF)

S6 Fig Data collapse for the mean cosine. The Langevin curves for the cosine of the alignment angle can be plotted as function of *MB*/*k*_B_*T*_eff_, with the effective temperature determined by fitting the Langevin function to the individual curves. In this case, all data for different run times collapse on one Langevin curve, when the cosine of the alignment angle is plotted as function of the effective Langevin parameter *MB*/*k*_B_*T*_eff_ with a run-time dependent effective temperature. Only small deviations from the theoretical curve are observed, but not as strong as the deviation seen for the histogram of the alignment angle. We emphasize that the effective temperature which we obtain by fitting the Langevin plots is different from the tumbling temperature, it depends on the mean tumble time and on the rotational friction coefficient and generally satisfies *T* < *T*_eff_ < *T*_tumble_.
(TIFF)

S7 Fig Velocity condensation. Alignment angle for run and tumble at *B* = 500 *μ*T, for 3D motion (purple curve) with and without thermal noise during runs (diamonds/stars) compared to a 2D projection (blue curve) with and without thermal noise (diamonds stars). While 3D and the 2D projections differ from each other when thermal noise during runs is present, when thermal noise is taken away the two cases coincide, and they show the velocity condensation phenomenon described by Rupprecht *et al*. [44]. In dark blue, the theoretical curve at small angles *P*_*θ*_ *θ*^−1+*KT*/*mB*^ [44]. The effect they describe results true only without thermal noise.
(TIFF)

S8 Fig Chemotaxis up a gradient in presence of a magnetic field. Chemotactic velocity as a function of the field inclination relative to the gradient for tumble (a) and reverse (b) at different magnetic field velocities. For a very strong magnetic field and tumble, we see that in the parallel case, the self velocity of 14.2 *μ*m is reached. For run and reverse, the velocities are always smaller than for the corresponding tumble case at 0°. The same plots are presented in (c) (tumble) and (d) (reverse), but here they are collapsed since now the velocity is normalized to 1. In black, the cosine of the magnetic angle for the tumble and of the absolute value of the cosine for reverse. Higher magnetic field follow the theoretical cosine better.
(TIFF)

S9 Fig Shortened runs down the gradient. We tested whether shortening runs down the gradient changes our results. When the bacterium is running down the gradient, in the model used in the main text the runs have a fixed duration (left Fig), in this case of 0.5 s, while the maximal run time up the gradient is of 1.5 s. In the right Fig instead, the runs down the gradient are shortened following a linear decrease up to a value of 0.5 s, reached at −∇*C*_0_ (right). The advantage of tumbling over reversals in the case of a parallel field is also seen with shortened runs down the gradient, but is less pronounced than without the shortening.
(TIFF)

S10 Fig Chemotactic velocities for tumbling with reversals. We tested two different mechanisms that combine tumbles with reversals. (a) In the first case, after each tumble the direction of motion is chosen randomly as either parallel or antiparallel to the body orientation mimicking the formation of a flagellar bundle on either side of the body (data in purple). In that case, motion up the gradient under an antiparallel magnetic field is possible, but slower than in the parallel case and slower than in the absence of the field. For comparison, tumble without the randomization of the direction of motion is shown in orange. (b) In the second case, the bacterium performs tumbles or reversals with certain probabilities at the end of each run. We simulated a combination of reversals and tumbles that interpolates smoothly between the two types of behaviors. We varied the fraction of reversals and found that swimming up the gradient against a magnetic field requires more than 50% reversals.
(TIFF)

S11 Fig Non-linear regime with antiparallel magnetic fields. We tested a scenario where runs up the gradient are very long and cells spend almost all time running as expected for a nonlinear chemotactic regime. Here, the run times up the gradient are 20 s, while the run times downward are 1 s. The averaged position of the bacteria as function of time is plotted for reverse and tumble. Positive values correspond to bacteria climbing up the gradient. Here, an antiparallel magnetic field is used and 100 cycles of run and change of directions are plotted. Reversing bacteria practically run always up the gradient with very long runs, while tumbling bacteria are forced to follow the antiparallel magnetic field, and are thus forced to swim down the gradient with very short runs.
(TIFF)

S12 FigBand width estimation.The size of the band is estimated in the main paper as the standard deviation of the position of the bacteria along the gradient. An alternative way to estimate it is the following: the density profile of the band after the equilibrium time (in this case for run and reverse of 477 s) is obtained (Fig (a)), integrating over all time-steps after the equilibration and normalizing it by the number of time-steps and number of bacteria. The profile can be well fitted with a Laplace distribution $f\left(x\right)=\frac{1}{2L}\text{exp}\left(-\frac{\left|x-m\right|}{L}\right)$, where the free fitting parameters are *m*, the position of the preferred concentration and *L*, the decay length of the curve. The curve is symmetrical with respect to the preferred concentration (which is situated at 2000 *μ*m and indicated by the red line for this case without magnetic field). *L* is thus a good estimator of the size of the band, since 68% of the density is situated in [*m* − *L*, *m* + *L*]. The bands seen in the simulations have a symmetric shape and their density profiles decay on both sides with the same decay length (this has been tested by fitting both sides separately with an exponential). Comparing the two approaches, we notice that the results show the same trends, but using the Laplace distribution fit leads to systematically smaller values. This is due to the fact that the standard deviation is influenced by the long tails of bacteria that are not in the band, which increase the estimate of the band width compared to *L*. In Fig (b), we show the band width obtained by the fit with the Laplace distribution for different magnetic field intensities, as a function of the magnetic field orientation.(TIFF)

S13 Fig Band formation for tumble with reverse. Band formation in a constant gradient for reverse, tumble, tumble with random change in orientation and mix of 50% reverse and 50% tumble at different magnetic field intensities. In the plot, the position of 100 bacteria is shown after 500 cycles of run and change of direction. The bacteria are initial distributed randomly within the black box at [1600, 2400] *μ*m. The preferred concentration is situated at 2000 *μ*m (red dotted vertical line). The *x* axis is in *μ*m, *y* axis has size of 800 *μ*m. While reverse and tumble with random orientation can form the band in any condition, pure tumble and a mixture of tumble and reverse perform chemotaxis poorly and do not form a band that is well localized around the preferred concentration.
(TIFF)

S14 Fig Choice of the free parameters for the capillary assay. The model of the capillary assay presents three free parameters that could not be determined by the experiments: *t*_up_ the run time towards a favorable direction, *t*_down_ towards an unfavorable direction, and the oxygen consumption *k*. First we varied the run times to get the desired band size (Fig a). We plot here the band size as function of the two run times for an antiparallel field of 50 *μ*T; the green plane corresponds to the desired experimental value. The blue points were obtained keeping *t*_down_ constant; the green points, keeping *t*_up_ constant; and the red points, keeping the ration between the two times constant. The winning point is the bright red case, obtained for *t*_up_ = 2 s and *t*_down_ = 0.9 s with *k* = 0.01 fmol min^−1^ cell^−1^ (see [12, 13]). The point size is proportional to the ratio of the times. Then, to match the band position dynamics (Fig b), the consumption constant *k* is varied. Smaller consumption constants make the dynamics slower. To match the experimental data points in green, we chose *k* = 0.005 fmol min^−1^ cell^−1^.
(TIFF)

S15 Fig Capillary assay at other magnetic field intensities. Since the model provides an effective description of the system, the effect of the magnetic field can be explored also in this capillary assay. The dynamics of the band position depends on the magnetic field intensity (a): stronger magnetic fields stabilize the band closer to the air-water interface thanks to the interplay between aerotaxis, oxygen consumption and magnetic fields. The band position is represented by the data points and the preferred concentration (C*) position by the red lines. Magnetic fields do not influence the band position at small times (below 5 min), when the leading dynamics is the oxygen flow as it can be seen from Fig (a), where pure oxygen flow -dashed red curve- matches the curves in the presence of bacteria. On the contrary, the magnetic field speeds the formation of the band up at small times before reaching the equilibrium: the band formed with the Earth magnetic field (blue curve of Fig (b) and with 500 *μ*T (yellow curve) is already symmetrical at 5 min and more dense compared to the band formed without magnetic field (in red). Therefore, in agreement with the results for a constant gradient, antiparallel magnetic fields help the band formation.
(TIFF)

S1 Table Simulation parameters for free bacteria. Simulation parameters for free swimming bacteria. Some comments are in order with respect to these parameters. (i) The friction coefficients are calculated assuming a spherical shape of the bacterium/microswimmer. The radius of the sphere was chosen to be 1*μ*m, a values compatible with the dimension of *E. coli* bacteria [28], as well as of some magnetotactic bacteria, in particular strain MSR-1 [11]. (ii) The magnetic moment was chosen to match the one of MSR-1 [57]. Here, a magnetic crystal with size 25 nm possesses a magnetization of ≃ 3.1 × 10^−17^ *Am*^2^. Considering a typical crystal of MSR-1 [58, 59] with radius 50nm, and considering a mean of 20-30 crystals per bacterium [11], we obtain the final value of 0.6 × 10^−3^ A *μ*m^2^, in good agreement with the magnetic moment calculated in [43]. (iii) Regarding the swimming speed, the mean run time with and without chemical gradients, and the mean tumble time, the parameters were chosen as measured for *E. coli* [28]. The parameters for magnetotactic bacteria have not been measured at the same level of detail, but swimming speeds tend to be larger and to vary greatly between studied strains [12]. (iv) The gradient was chosen constant in space and time, with a value similar to the serine gradient experienced by the bacteria in the experiments of Berg and Brown [28]. Oxygen gradients that built up dynamically in experiments with magnetotactic bacteria were seen to have similar values [12]. The preferred concentration was chosen in the range of oxygen concentration preferred by microaerophilic bacteria [13, 60].
(PDF)

S2 Table Simulation parameters for bacteria in capillaries. Simulation parameters for capillary assays. If not stated here, the parameters are unchanged with respect to S1 Table. For the capillary assay, the oxygen concentration and gradient are dynamic and not constant. The run-time response function to the gradient is a step function, thus the reference gradient ∇*C*_0_ is 0.
(PDF)

## References

[1] Elgeti J., Winkler R. G., Gompper G., Physics of microswimmers—single particle motion and collective behavior: a review. Reports on Progress in Physics 2015;78(5):056601 10.1088/0034-4885/78/5/056601 25919479

[2] Bechinger C., Di Leonardo R., Löwen H., Reichhardt C., Volpe G., Active particles in complex and crowded environments. Reviews of Modern Physics 2016;88(4):045006 10.1103/RevModPhys.88.045006

[3] Berg H. C., Random Walks in Biology. Princeton Paperbacks 1993; Chapter 6.

[4] Sourjik V., Wingreen N. S., Responding to chemical gradients: bacterial chemotaxis. Current opinion in cell biology 2012;24(2):262–8. 10.1016/j.ceb.2011.11.008 22169400 PMC3320702

[5] Klumpp S., Faivre D., Magnetotactic bacteria. The European Physical Journal Special Topics 2016;225(11-12):2173–2188. 10.1140/epjst/e2016-60055-y

[6] Frankel R. B., Williams T. J., Bazylinski D. A., Magneto-Aerotaxis in: Magnetoreception and Magnetosomes in Bacteria (Springer, Berlin and Heidelberg, 2007), pp.1–24.

[7] Klumpp S., Lefevre T. C., Bennet M., Faivre D., Swimming with magnets: from biological organisms to synthetic devices. Physics Reports 2019;789:1–54. 10.1016/j.physrep.2018.10.007

[8] Carlsen R. W., Sitti M., Bio-Hybrid Cell-Based Actuators for Microsystems. Small 2014;10(19):3831–3851. 10.1002/smll.201400384 24895215

[9] Dreyfus R., Baudry J., Roper M. L., Fermigier M., Stone H. A., Bibette J., Microscopic artificial swimmers Nature 2005;437:862–865. 10.1038/nature04090 16208366

[10] Ghosh A., Paria D., Singh H. J., Venugopalan P. L., Ghosh A., Dynamical configurations and bistability of helical nanostructures under external torque. Physical Review E 2012;86(3):031401 10.1103/PhysRevE.86.031401 23030914

[11] Frankel R. B., Magnetic guidance of organisms Annual review of biophysics and bioengineering 1984;13:85–103. 10.1146/annurev.bb.13.060184.000505 6378076

[12] Lefèvre C. T., Bennet M., Landau L., Vach P., Pignol D., Bazylinski D. A., Frankel R. B., Klumpp S., Faivre D., Diversity of Magneto-Aerotactic Behaviors and Oxygen Sensing Mechanisms in Cultured Magnetotactic Bacteria Biophysical Journal 2014;107(2):527–538. 10.1016/j.bpj.2014.05.043 25028894 PMC4104051

[13] Bennet M., McCarthy A., Fix D., Edwards M. R., Repp F., Vach P., Dunlop J. W. C., Sitti M., Buller G. S., Klumpp S., Faivre D., Influence of Magnetic Fields on Magneto-Aerotaxis. PLOS One 2014;9:1–10. 10.1371/journal.pone.0101150 PMC4077765 24983865

[14] Smith M. J., Sheehan P. E., Perry L. L., O’Connor K., Csonka L. N., Applegate B. M., Whitman L. J., Quantifying the Magnetic Advantage in Magnetotaxis. Biophysical journal 2006;91(3):1098–1107. 10.1529/biophysj.106.085167 16714352 PMC1563769

[15] Mao X., Egli R., Petersen N., Hanzlik M., Liu X., Magneto-chemotaxis in sediment: First insights. PLoS One 2014;9(7):e102810 10.1371/journal.pone.0102810 25032699 PMC4102565

[16] Park B.-W., Zhuang J., Yasa O., Sitti M. Multifunctional Bacteria-Driven Microswimmers for Targeted Active Drug Delivery. ACS Nano 2017 10.1021/acsnano.7b03207 28873304

[17] Bente K., Codutti A., Bachmann F., Faivre D., Biohybrid and Bioinspired Magnetic Microswimmers. Small 2018;1704374. 10.1002/smll.201704374 29855143

[18] Felfoul O., Mohammadi M., Taherkhani S., de Lanauze D., Zhong Xu Y., Loghin D., Essa S., Jancik S., Houle D., Lafleur M., Gaboury L., Tabrizian M., Kaou N., Atkin M., Vuong T., Batist G., Beauchemin N., Radzioch D., Martel S., Magneto-aerotactic bacteria deliver drug-containing nanoliposomes to tumour hypoxic regions. Nature nanotechnology 2016;11:941–947. 10.1038/nnano.2016.137 27525475 PMC6094936

[19] Alt W., Biased Random Walk Models for Chemotaxis and Related Diffusion Approximations. Journal of mathematical biology 1980;9:147–177. 10.1007/bf00275919 7365332

[20] Schnitzer M. J., Theory of continuum random walks and application to chemotaxis. Physical Review E 1992;48(4):2553 10.1103/PhysRevE.48.2553 9960890

[21] Romanczuk P., Bär M., Ebeling W., Lindner B., Schimansky-Geier L., Active brownian particles. The European Physical Journal Special Topics 2012;202(1):1–162. 10.1140/epjst/e2012-01529-y

[22] Kalinin Y. V., Jiang L., Tu Y., Wu M., Logarithmic sensing in *Escherichia coli* bacterial chemotaxis. Biophysical journal 2009;96(6):2439–2448. 10.1016/j.bpj.2008.10.027 19289068 PMC2989150

[23] Khatami M., Wolff K., Pohl O., Ejtehadi M. R., Stark H., Active Brownian particles and run-and-tumble particles separate inside a maze. Scientific Reports 2016;6:37670 10.1038/srep37670 27876867 PMC5120314

[24] Takatori S. C., Brady J. F., Swim stress, motion, and deformation of active matter: effect of an external field. Soft Matter 2014;10:9433–9445. 10.1039/c4sm01409j 25330273

[25] Perez Ipina E., Otte S., Pontier-Bres R., Czerucka D., Peruani F., Bacteria display optimal transport near surfaces Nature Physics 2019; 15(6):610–615. 10.1038/s41567-019-0460-5

[26] Saragosti J., Silberzan P., Buguin A., Modeling *E. coli* Tumbles by Rotational Diffusion. Implications for Chemotaxis. PLoS ONE 2012;7(4):e35412 10.1371/journal.pone.0035412 22530021 PMC3329434

[27] Taute K. M., Gude S., Tans S. J., Shimizu T. S., High-throughput 3D tracking of bacteria on a standard phase contrast microscope. Nature Communications 2015;6:8776 10.1038/ncomms9776 26522289 PMC4659942

[28] Berg H. C., Brown D. A., Chemotaxis in *Escherichia coli* analysed by Three-dimensional Tracking. Nature 1972;239:500–504. 10.1038/239500a0 4563019

[29] Brown D. A., Berg H. C., Temporal stimulation of chemotaxis in *Escherichia coli*. Proceedings of the National Academy of Sciences 1974;71(4):1388–1392. 10.1073/pnas.71.4.1388 PMC388234 4598304

[30] Celani A., Vergassola M., Bacterial strategies for chemotaxis response. Proceedings of the National Academy of Sciences 2010;107(4):1391–1396. 10.1073/pnas.0909673107 PMC2824349 20080704

[31] Taylor B. L., Koshland D. E. Jr,. Reversal of Flagellar Rotation in Monotrichous and Peritrichous Bacteria: Generation of Changes in Direction. Journal of bacteriology 1974;119(2):640–642. 4605064 10.1128/jb.119.2.640-642.1974 PMC245654

[32] Popp F., Armitage J. P., Schüler D., Polarity of bacterial magnetotaxis is controlled by aerotaxis through a common sensory pathway. Nature communications 2014;5:5398 10.1038/ncomms6398 25394370

[33] Reichert M., Hydrodynamic Interactions in Colloidal and Biological Systems. Dr. Rer. Nat. Dissertation at Konstanz University 2006.

[34] Press W. H., Teukolsky S. A., Vetterling W. T., Flannery B. P., Numerical Recipes in FORTRAN (2Nd Ed): The Art of Scientific Computing. Cambridge University Press 1992.

[35] Risken H., The Fokker-Planck Equation. Springer 1989.

[36] Rismani Yazdi S., Nosrati R., Stevens C. A., Vogel D., Escobedo C., Migration of magnetotactic bacteria in porous media. Biomicrofluidics 2018;12(1):011101 10.1063/1.5024508 29531633 PMC5828923

[37] Heyen U., Schüler D., Growth and magnetosome formation by microaerophilic Magnetospirillum strains in an oxygen-controlled fermentor. Applied Microbiology and Biotechnology 2003;61:536–544. 10.1007/s00253-002-1219-x 12764570

[38] Lovely P. S., Dahlquist F. W., Statistical measures of bacterial motility and chemotaxis. Journal of theoretical biology 1975;50(2):477–496. 10.1016/0022-5193(75)90094-6 1094203

[39] Zahn C., Keller S., Toro-Nahuelpan M., Dorscht P., Gross W., Laumann M., Gekle S., Zimmermann W., Schüler D., Kress H., Measurement of the magnetic moment of single Magnetospirillum gryphiswaldense cells by magnetic tweezers. Scientific Reports 2018;7. 10.1038/s41598-017-03756-z PMC5472611 28620230

[40] Long J., Zucker S.W., Emonet T., Feedback between motion and sensation provides nonlinear boost in run-and-tumble navigation. PLoS Comput Biol 2017, 13(3): e1005429 10.1371/journal.pcbi.1005429 28264023 PMC5358899

[41] De Barros E. L., Acosta-Avalos D., A simple method to estimate the magnetic moment of magnetic micro-particles. Journal of Magnetism and Magnetic Materials 2008;320 10.1016/j.jmmm.2008.02.144

[42] Frankel R. B., Blakemore R. P., Navigational compass in magnetotactic bacteria. Journal of Magnetism and Magnetic Materials 1980;15-18;1562–1564. 10.1016/0304-8853(80)90409-6

[43] Nadkarni R., Barkley S., Fradin C., A Comparison of Methods to Measure the Magnetic Moment of Magnetotactic Bacteria through Analysis of Their Trajectories in External Magnetic Fields. PLOS One 2013;8(12). 10.1371/journal.pone.0082064 PMC3861366 24349185

[44] Rupprecht J.-F., Waisbord N., Ybert C., Cottin-Bizonne C., Bocquet L., Velocity Condensation for Magnetotactic Bacteria. Physical review letters 2016;116(16):168101–168106. 10.1103/PhysRevLett.116.168101 27152825

[45] Murat D., Hérisse M., Espinosa L., Bossa A., Alberto F., Wu L.-F., Opposite and Coordinated Rotation of Amphitrichous Flagella Governs Oriented Swimming and Reversals in a Magnetotactic Spirillum. Journal of bacteriology 2015;197(20):3275–3282. 10.1128/JB.00172-15 26240070 PMC4573727

[46] Jiang L., ouyang Q., Tu Y., Quantitative modeling of *Escherichia coli* chemotactic motion in environments varying in space and time. PLoS Comput. Bio. 2010;6(4):e1000735 10.1371/journal.pcbi.1000735 20386737 PMC2851563

[47] Dufour Y.S., Fu X., Hernandez-Nunez L., Emonet T., Limits of feedback control in bacterial chemotaxis. PLoS Comput. Bio. 2014;10(6):e1003694 10.1371/journal.pcbi.1003694 24967937 PMC4072517

[48] Zhou S., Tovkach O., Golovaty D., Sokolov A., Aranson I.S., Lavrentovich O.D., Dynamic states of swimming bacteria in a nematic liquid crystal cell with homeotropic alignment. New J. Phys. 2017;19:055006. 10.1088/1367-2630/aa695b

[49] https://www.ptb.de/cms/nc/en/ptb/fachabteilungen/abt2/fb-25/ag-251/live-data-earths-magnetic-field.html.

[50] Mazzag B. C., Zhulin I. B., Mogilner A., Model of bacterial band formation in aerotaxis. Biophysical journal 2003;85(6);3558–3574. 10.1016/S0006-3495(03)74775-4 14645050 PMC1303662

[51] Frankel R. B., Blakemore R. P., De Araujo F. F. T., Esquivel D. M. S., Danon J., Magnetotactic bacteria at the geomagnetic equator. Science 1981;212(4500):1269–70. 10.1126/science.212.4500.1269 17738834

[52] Rismani Yazdi S., Nosrati R., Stevens C. A., Vogel D., Davies P. L., Escobedo C., Magnetotaxis Enables Magnetotactic Bacteria to Navigate in Flow. Small 2017;14(5):1702982 10.1002/smll.201702982 29205792

[53] Bente K., Mohammadinejad S., Bachmann F., Codutti A., Lefèvre C. T., Klumpp S., Faivre D., High-speed Helical Microswimming and Rapid Reorientations of Bacteria. in preparation.

[54] Stanton M. M., Park B.W., Vilela D., Bente K., Faivre F., Sitti M., and Sanchez S., Magnetotactic bacteria powered biohybrids target E. coli biofilms. ACS Nano 2017;11:9968 10.1021/acsnano.7b04128 28933815

[55] Waite A.J., Frankel N.W., Dufour Y. S., Johnston J. F., Long J., Emonet T., Non-genetic diversity modulates population performance Mol. Sys. Biol. 2016, 12: 895 10.15252/msb.20167044 PMC5199129 27994041

[56] Gómez Nava L., Großmann R., and Peruani F., Markovian robots: Minimal navigation strategies for active particles. Phys. Rev. E 2018;97:042604. 10.1103/PhysRevE.97.042604 29758683

[57] Klumpp S., Kiani B., Vach P. and Faivre D., Navigation with magnetic nanoparticles: magnetotactic bacteria and magnetic micro-robots. Physica Scripta 2015;T165:014044. 10.1088/0031-8949/2015/T165/014044

[58] Devouard B., Posfai M., Hua X., Bazylinski D. A., Frankel R.B. and Buseck P.R., Magnetite from magnetotactic bacteria: Size distributions and twinning”. Am. Mineral. 1998;83:1387–1398. 10.2138/am-1998-11-1228

[59] Faivre D., Menguy N., Posfai M. and Schüler D., Environmental parameters affect the physical properties of fast-growing magnetosomes. Am. Mineral. 2008;93:463–469. 10.2138/am.2008.2678

[60] Thar R. and Fenchel T., Survey of motile microaerophilic bacterial morphotypes in the oxygen gradient above a marine sulfidic sediment. Appl. and Environ. Microbiol. 2005;71:3682–3691. 10.1128/AEM.71.7.3682-3691.2005 16000777 PMC1168987

