# Supplementary material for: Chemotaxis in external fields: Simulations for active magnetic biological matter
Source: PLoS Comput Biol. 2019 Dec 19;15(12):e1007548. doi: 10.1371/journal.pcbi.1007548 (PMC6941824; doi:10.1371/journal.pcbi.1007548)
Supplement: S1 Text — Implementation of chemotaxis for attractants, reppellents and preferred concentration, available as an attachment. (PDF) [file pcbi.1007548.s001.pdf]

1 S1 Text - Implementation of chemotaxis

The average run time τ_{run} is modulated by a chemical gradient for chemotaxis. For a chemoattractant, we use the following simple implementation of that modulation (other functional dependencies can also be used [29,30]):

$$\tau_{\mathrm{run}} = \begin{cases} \tau_0 & \text{for} \quad \nabla C_{\parallel} \leq 0 \\ \tau_0 \left(1 + \frac{\nabla C_{\parallel}}{\nabla C_0}\right) & \text{for} \quad 0 < \nabla C_{\parallel} \leq \nabla C_0 \\ 2\tau_0 & \text{for} \quad \nabla C_{\parallel} > \nabla C_0 \end{cases} \tag{1}$$

where τ_0 indicates the mean run time in absence of gradients, $\nabla C_{\parallel}$ indicates the projection of the chemical gradient onto the direction of motion and ∇C_0 is a threshold gradient for which the maximal run time is reached. For a chemorepellent, a corresponding expression is used, modified such that runs down the gradient are prolonged,

$$\tau_{\mathrm{run}} = \begin{cases} \tau_0 & \text{for} \quad \nabla C_{\parallel} \geq 0 \\ \tau_0 \left(1 - \frac{\nabla C_{\parallel}}{\nabla C_0}\right) & \text{for} \quad -\nabla C_0 < \nabla C_{\parallel} \leq 0 \\ 2\tau_0 & \text{for} \quad \nabla C_{\parallel} \leq -\nabla C_0. \end{cases} \tag{2}$$

Finally, if the bacterium is attracted to a preferred concentration C^*, then Eq. (1) is used for $C < C^*$ and Eq. (2) for $C \geq C^*$. We note that, via this condition, the run times depend not only on the concentration gradient, but also on the concentration itself.
