# Supplementary material for: Chemotaxis in external fields: Simulations for active magnetic biological matter
Source: PLoS Comput Biol. 2019 Dec 19;15(12):e1007548. doi: 10.1371/journal.pcbi.1007548 (PMC6941824; doi:10.1371/journal.pcbi.1007548)
Supplement: S2 Text — Method to calculate the chemotactic velocity for an attractant, available as an attachment. (PDF) [file pcbi.1007548.s002.pdf]

1 S2 Text- Chemotactic velocity for an attractant

To calculate the chemotaxis velocity up the gradient, two methods can be used that lead to compatible estimations. For the first method, the mean square displacement (MSD) is calculated, as a time-averaged MSD (TAMSD), then averaged over the ensemble of trajectories ($\langle \text{TAMSD} \rangle_{\text{ensemble}}$). At large times the slope of the mean square displacement in the log-log plot is again 2, due to the effective velocity that arises from the chemotactic bias. The intercept I of the fitting line at large times thus is connected to the chemotactic velocity as $I = \log(v_{\text{taxis}}^2)$. From the fit of the data we obtain $v_{\text{taxis}} = (1.082 \pm 0.003)$ μm/s, where the error is the one of the fit, underestimating the real error arising from more repetitions of the experiment. The second method instead considers many trajectories and it takes the mean position. The mean position is a linear function of time, and the slope gives the velocity. For small times, the slope is the self velocity, but at larger times, the slope gives the chemotactic velocity. In the case of tumble without magnetic field and forces, the velocity is (1.04 ± 0.02) μm/s (Fig. 2a in paper). The error is estimated considering two methods. In the first method to estimate the error, 10 repetitions of the experiment have been done, and then the standard deviation has been calculated. This standard deviation can be associated as error of one of the single pool of experiments. In the second case, we considered ten subsets of one pool of data, and we estimated the standard error of the mean over these subsets. Both evaluation lead to a reasonable estimate of the error of 0.02 μm/s.
