# Supplementary material for: Chemotaxis in external fields: Simulations for active magnetic biological matter
Source: PLoS Comput Biol. 2019 Dec 19;15(12):e1007548. doi: 10.1371/journal.pcbi.1007548 (PMC6941824; doi:10.1371/journal.pcbi.1007548)
Supplement: S3 Text — Derivation of the alignment angle in the presence of magnetic fields, available as an attachment. (PDF) [file pcbi.1007548.s003.pdf]

1 S3 Text - Alignment time in the presence of magnetic fields

The theoretical alignment time to a magnetic field was obtained from the equations of motion in the following way (the approach is equivalent to that in refs. [11,41]): We consider a run in the absence of thermal noise, but in the presence of a magnetic field, which for simplicity we take to be oriented along the z axis, $\hat{z}$, $\mathbf{B} = B\hat{z}$. The equations for the rotational frequency $\boldsymbol{\omega}$ and for the orientation vector $\mathbf{e}$ are

$$\boldsymbol{\omega} = \frac{\mathrm{d}\boldsymbol{\phi}}{\mathrm{d}t} = MB\gamma_r^{-1}(\mathbf{e} \times \hat{z})$$
$$\mathrm{d}\mathbf{e} = \mathrm{d}\boldsymbol{\phi} \times \mathbf{e}.$$

Substituting the second equation into the first one and writing out equations for the components of the orientation vector, we obtain for the component e_{z} parallel to the magnetic field

$$\frac{\mathrm{d}e_{\mathrm{z}}}{\mathrm{d}t} + \frac{1}{\tilde{\tau}}(e_{\mathrm{z}}^2 - 1) = 0, \tag{1}$$

where

$$\tilde{\tau} = \frac{\gamma_{\mathrm{r}}}{MB} \tag{2}$$

is the typical relaxation time. Solving the equation for e_{z}, we find

$$e_{\mathrm{z}} = \frac{\exp{(2t/\tilde{\tau})} - c}{\exp{(2t/\tilde{\tau})} + c}, \tag{3}$$

where $c = \frac{1-e_{\mathrm{z0}}}{1+e_{\mathrm{z0}}}$ is given by the initial condition $e_{\mathrm{z}}(t=0) = e_{\mathrm{z0}}$. Since we consider a magnetic field along the z axis, we can express e_{z} as $e_{\mathrm{z}} = \cos(\theta_{\mathrm{e,B}})$. Now we take the initial condition for e_{z} as resulting from a kick away from alignment with the field due to tumble, then on average we have $e_{\mathrm{z0}} = \langle\cos(\theta_{\mathrm{tumble}})\rangle \simeq 0.31$ (the numerical value is from the adjusted mean tumbling angle, see above). Our equation thus describes a decay of the (cosine of the) alignment angle back to alignment with the field. See S5 Fig for a fit of the cosine of the alignment angle.
