# Supplementary material for: Chemotaxis in external fields: Simulations for active magnetic biological matter
Source: PLoS Comput Biol. 2019 Dec 19;15(12):e1007548. doi: 10.1371/journal.pcbi.1007548 (PMC6941824; doi:10.1371/journal.pcbi.1007548)
Supplement: S4 Text — Derivation of the of the alignment angle distribution and the mean cosine of the alignment angle, available as an attachment. (PDF) [file pcbi.1007548.s004.pdf]

1 S4 Text - The alignment angle distribution and the mean cosine of the alignment angle

During runs the bacteria passively align with the magnetic field, but the influence of thermal noise perturbs the alignment. Since the noise is thermal, the fluctuations are characterized by the corresponding energy $E = -MB\cos\theta_{\mathrm{e,B}}$ [11] and given by Boltzmann statistics $\propto \exp -E/k_{\mathrm{B}}T$. Using spherical coordinates, we obtain the following distribution for the alignment angle $\theta_{\mathrm{e,B}}$:

$$p(\theta_{\mathrm{e,B}}) = \tfrac{MB}{k_{\mathrm{B}}T} \frac{1}{2\sinh(\frac{MB}{k_{\mathrm{B}}T})} \sin\theta_{\mathrm{e,B}} \exp(\tfrac{MB}{k_{\mathrm{B}}T}\cos\theta_{\mathrm{e,B}}), \tag{1}$$

where the term $\sin\theta_{\mathrm{e,B}}$ comes from the area element. This expression is normalized as $\int_0^\pi \mathrm{d}\theta p(\theta, T) = 1$. Using this distribution to calculate the mean cosine of the alignment angle, we obtain the Langevin function [11,42]:

$$\langle\cos(\theta_{\mathrm{e,B}})\rangle = \int_0^\pi \cos(\theta_{\mathrm{e,B}}) p(\theta_{\mathrm{e,B}}) \mathrm{d}\theta_{\mathrm{e,B}} = \coth(MB/k_{\mathrm{B}}T) - (MB/k_{\mathrm{B}}T)^{-1}. \tag{2}$$

The alignment angle follows this distribution during runs. When a change of direction takes place, it will, in general, change. This is however not the case for run and reverse, where bacterium remains aligned with the field and the inversion takes place without an active rotation of the body. Thus in this case, the distribution remains valid at all times. For run and tumble, however, the tumbling event perturb the alignment and reorient the body with a mean angle of 68°, thus the distribution of the angle changes, as described in the main text.
