# Supplementary material for: Chemotaxis in external fields: Simulations for active magnetic biological matter
Source: PLoS Comput Biol. 2019 Dec 19;15(12):e1007548. doi: 10.1371/journal.pcbi.1007548 (PMC6941824; doi:10.1371/journal.pcbi.1007548)
Supplement: S1 Source Code — (PDF) [file pcbi.1007548.s005.pdf]

Read me

This code allows to simulate a capillary experiment with a dynamic gradient for magnetotactic bacteria.

Save the single files with extension .f90. Install the gfortran compiler.

To compile the code, use:

gfortran -o clean first.f90 fifth.f90 fourth.f90 second.f90 third.f90 sixth.f90 MAIN.f90 -fbounds-check

To run the code, use:

./clean

The main parameters (as bacteria velocity, density, run times, magnetic fields etc.) can be changed in the file first_bact_mag.f90. Additional chemotactic parameters can be changed in the file fifth_bact_O2.f90.

Main outputs:
In the output file density_in_time,dat each row corresponds to different times and each column gives the bacteria density in the spatial bins.
In the output file concentration_in_time.dat each row corresponds to different times and each column gives the oxygen concentration in the spatial bins.

```
  !
@@@@@@@@@@@@@@@@@@@@@@@@@@@@@@@@@@@@@@@@@@@@@@@@@@@@@@@@@@@@@@@@@@@@
@@@@@@@@@@@@@@@@@@@@@@@@@@@@
  !-------------------------------------------------------------------
------------------------------
  !@@@@@@@@@@@@@@@@@@@@@@@@@@     initialisation
@@@@@@@@@@@@@@@@@@@@@@@@@@@@@@@@@@@@@@@@@@@@@@
  !-------------------------------------------------------------------
-------------------------------
  !
@@@@@@@@@@@@@@@@@@@@@@@@@@@@@@@@@@@@@@@@@@@@@@@@@@@@@@@@@@@@@@@@@@@@
@@@@@@@@@@@@@@@@@@@@@@@@@@@@
  !@@@@@@@@@@ AUTHOR: Dr. CODUTTI AGNESE, agnese.codutti@ds.mpg.de




MODULE first_bact
IMPLICIT NONE
  integer, parameter:: sp = kind(1.d0)
  private:: sp
  CONTAINS




   SUBROUTINE
initializer(D,num_bac,final_time,deltat,v_self,x,e,gamma_t,gamma_r,T
,Teff,t1_0,grad_ref,t2_medio,&

t_run,t_tumble,B,M,modB,cstar,kindofchemotaxis,filechoice,&

randomorientation,reversechoice,a,F,&

randomx0,deltax,deltay,deltaz,L,Lyz,ndeltax,ndeltay,ndeltaz,initial_
conc,&

Diffcoeff,kO2,ca,O2left,freq_writing)

    implicit none



    INTEGER, intent(out):: D
    INTEGER ,intent(out):: num_bac
    real(kind=sp),intent(out):: final_time
    REAL(kind=sp),intent(out) :: deltat, v_self
    REAL(kind=sp), DIMENSION(:),ALLOCATABLE,intent(out):: x,e
    REAL(kind=sp),intent(out):: gamma_r,gamma_t
    REAL(kind=sp),intent(out) :: T, Teff
    REAL(kind=sp),intent(out) :: t2_medio
    REAL(kind=sp),intent(out) :: t_run,t_tumble
    real(kind=sp), intent(out) :: M
    real(kind=sp),dimension(0:2),intent(out) :: B
```

```
    real(kind=sp),intent(out) ::modB
    real(kind=sp),intent(out) :: t1_0,grad_ref
    real(kind=sp),intent(out)::cstar
    integer,intent(out)::kindofchemotaxis
    integer,dimension(6),intent(out) :: filechoice
    integer,intent(out):: randomorientation,randomx0
    integer:: counter
    real(kind=sp):: r1,r2,e22,r3
    integer :: seed_dim
    integer,dimension(:), allocatable :: seed
    integer:: clock,iiiii
    logical:: exist
    integer,intent(out):: reversechoice
    character(len=3),intent(out) :: a
    real(kind=sp),dimension(0:2),intent(out) :: F
    real(kind=sp) ::modF
    integer::i_e,i_x
    real(kind=sp),intent(out) :: deltax,L,Lyz,deltay,deltaz
    integer,intent(out):: ndeltax,ndeltay,ndeltaz
    real(kind=sp),intent(out):: initial_conc
    real(kind=sp),intent(out):: Diffcoeff,kO2,ca,O2left
    real(kind=sp)::conversion
    integer,intent(out):: freq_writing
!
##################################################################
########################################
!
##################################################################
########################################
!
##################################################################
########################################



!----choose which file to
print-------------------------------------------
!'enter 1 for the file you want to print, 0 if not:'
print*, 'array: 1 angles_tumble.dat,2 angles_run.dat,3 log.dat,4
traj.dat,5 vel.dat,6 relax.dat'
filechoice=(/0,0,1,0,0,0/)
print*, filechoice

freq_writing=30000 !for dt=0.002s this number correspond to write
files every n seconds. example fw=50->n=0.1s
!-----------------------------------------------------------------
----------

reversechoice=1                                                 !
1=reverse,0=tumble
randomorientation=1                                             !
1=random initial orientation,0=fixed initial orientation
randomx0=1                                                      !
1=random initial position, 0=fixed initial position
```

```
!*****************************************************
D=3                                                                  !
can be 2D or 3D
num_bac=13900                                                        !
bacteria number, corresponding to a density of OD=1.001
!*****************************************************





deltax=20.                                                           !
mum, spatial bin for spatial integration
ndeltax=2000                                                         !
number of bins
L=ndeltax*deltax                                                     !
Length on the x direction (long part of the tube)

deltay=deltax
ndeltay=5
deltaz=deltax
ndeltaz=5
Lyz=deltay*ndeltay                                                   !
square section

deltat=0.002                                                         !
Integration timestep, seconds
final_time=1200.                                                     !
final time, seconds
v_self=50.!14.2                                                      !
Bacterium self velocity, mum/s

a='1.0'                                                              !
bacterial radius in mum
!a='1.5'
!a='2.0'
!a='mix'
!a='exp'

!@@@@@gamma depending on radius@@@@@@@@@@@@@@@@                      !
rotational and friction coefficients, calculated for a sphere of
given radius
SELECT CASE (a)
   CASE ('1.0')
       gamma_t=5.1*10**(-8.)                                         !
Kg/s for sphere ray 1mum
       gamma_r=6.8*10**(-8.)                                         !
mum^2 Kg/s for sphere of 1mum  ray
   CASE ('1.5')
       gamma_t=7.6*10**(-8.)                                         !
Kg/s for spheare ray 1.5mum
```

```
       gamma_r=1.*10**(-7.)                                        !
mum^2 Kg/s for spheare of 1.5mum  ray
   CASE ('2.0')
       gamma_t=1. * 10**(-7.)
       gamma_r=5.4 *10**(-7.)
   CASE ('mix')                                                    !
decoupled gammat and gammar
        gamma_t=5.1*10**(-8.)                                      !
from a=1    (normal size)
        gamma_r=1.*10**(-7.)                                       !
from a=1.5 (from mean angle during run for berg)
   CASE('exp')                                                     !
decoupled gammat and gammar
      gamma_r=2.8*10**(-10.)                                       !
from experimental translational friction of dead MSR1
      gamma_t=6.*10**(-9.)                                         !
experimental
END SELECT

!@@@@@@@@@@@@@@@@@@@@@@@@@@@@@@@@@@@@@@@@@@@@@@



!--------------------temperatures--------------
T=305.                                                             !
room temperature, K



SELECT CASE (a)                                                    !
effective tumbling temperature
   CASE ('1.0')
        Teff=4.2*10**4.                                            !
correct for 1mum
   CASE ('1.5')
        Teff=6.15*10**4.                                           !
correct for 1.5mum
   CASE ('2.0')
        Teff=3.32*10**5.                                           !
correct fo radius 2mum
   CASE ('mix')
        Teff=6.15*10**4.                                           !
correct for 1.5mum (due mainly to gammar)
   CASE ('exp')
       Teff=305.                                                   !
exp data are for bacteria doing reverse, so no tumble events
END SELECT


!----------------------------------------------
```

```
!---------------time for TUMBLE------------------

t2_medio=0.14                                                     !
mean tumbling time, from berg
t_run=0.                                                          !
run time, initialization
t_tumble=0.                                                       !
tumble time, initialization


!---------------time for RUN: determined in the end of this
function, depends on chemotaxis

!---constant force----
modF=0.                                                           !
modulus of an external constant force in mum kg/s^2


F(0)=0.                                                           !
force orientation
F(1)=0.
F(2)=-1.

F=F/sqrt(F(0)**2+F(1)**2+F(2)**2)
F=F*modF
!---------------------


!-------------magnetic field parameters------------

M=0.6*10**(-3.)                                                  !
modulus of the magnetic moment in A microm**2, directed along the
orientation-axis of the bacterium

!modB=5.*10**(-5.)                                                !
modulus of the external magnetic field in T=kg A**-1 s**-2
(equivalent to 50 microTesla, earth magnetic field)
modB=0.
!modB=50.*10**(-4.)                                               !10
times the magnetic field

B(0)=1.!-1.!cos(acos(-1.)/6.)!0.                                 !
direction of the magnetic field
B(1)=0.!0.!sin(acos(-1.)/6.)
B(2)=0.!1.
B=B/sqrt(B(0)**2+B(1)**2+B(2)**2)                                !
versor of B
B=B*modB                                                         !
vector with correct direction and correct modulus
!---------------------------------------------------
```

```
!
*******************************************************************
*****************
!CHEMOTAXIS PARAMETERS

!chemotaxis choice:
!kindofchemotaxis=100                                              !
dummy value, no chemotaxis
kindofchemotaxis=0                                                 !
with preferred concentration cstar, to form a band
!kindofchemotaxis=1                                                !
towards maximum - attractant
!kindofchemotaxis=2                                                !
towards minimum - repellent




 grad_ref=25.                                                      !
muM/mm, maximum gradient that bacteria can sense


 cstar=3.                                                          !
muMolar, preferred concentration

 initial_conc=216                                                  !
muM !initial concentration of oxygen at the air water interface
 Diffcoeff=2100.                                                   !
oxygen diffusion coefficient, mum**2/s


 kO2=0.005                                                         !
fmol/min/cell consumption parameter
 !to covert mol in Molar, you have to divide by volume of the slice
(in Liter)
 kO2=kO2*10**(6.)/(60*deltay*deltaz*deltax)  !muM/cell/s


 ca=0.75                                                           !
muM !O2 at which consumption is half maximal
 O2left=initial_conc                                               !
muM,concentration at boundaries



!-------------------------------------------



!--------------RUN time
initialization-------------------------------------

if(kindofchemotaxis>=0 .and. kindofchemotaxis<=2) then
        t1_0=1.                                                    !s,
```

```
mean run time with constant chemical concentration
else
        t1_0=0.86                                              !s,
mean run time with NO chemicals
end if
!-------------------------------------------------------------
!
********************************************************************
*****************




!*****************initial conditions on the position and
orientation*************************************************
!@@@@@@@@@@@@@@@@@@@@@@@@@@@@@@@@@
!
********************************************************************
******************



ALLOCATE ( x(0:3*num_bac-1) )                                      !
position of bacterium 1 x y z, bacterium 2 x y z and so on; in 2D
Z=0 ALWAYS
ALLOCATE ( e(0:3*num_bac-1) )                                      !
orientation vector


!----------initial position------
! to have a fixed initial position
if(randomx0==0)then
x=0.
end if

!TO HAVE RANDOMIZED INITIAL POSITIONS
if(randomx0==1)then
    x=0.
    call random_seed(size=seed_dim)
    allocate(seed(seed_dim))
    CALL SYSTEM_CLOCK(COUNT=clock)
    seed = clock + 37 * (/ (iiiii - 1, iiiii = 1, seed_dim) /)
    call random_seed(put=seed)

    !----BACTERIA MUST HAVE X BETWEEN 0 AND L TO BE IN THE BOX
    do i_x=1,num_bac
      call random_number(r1)
      x(0+(i_x-1)*3)=r1*L

      call random_number(r1)
      x(1+(i_x-1)*3)=r1*Lyz!r1*800.-400.
```

```
      call random_number(r1)
      x(2+(i_x-1)*3)=r1*Lyz!r1*800.-400.
    end do

    deallocate(seed)

end if
!--------------------------------


!--------initial orientation------------
e=0.

!fixed orientaion
if(randomorientation==0) then
do i_e=1,num_bac
 e(0+(i_e-1)*3)=0.58
 e(1+(i_e-1)*3)=0.58
 e(2+(i_e-1)*3)=sqrt(1.-e(0+(i_e-1)*3)**2-e(1+(i_e-1)*3)**2)
end do
end if

!...to give random orientation....

if(randomorientation==1) then


    call random_seed(size=seed_dim)
    allocate(seed(seed_dim))
    CALL SYSTEM_CLOCK(COUNT=clock)
    seed = clock + 37 * (/ (iiiii - 1, iiiii = 1, seed_dim) /)
    call random_seed(put=seed)


    do i_e=1,num_bac


      call random_number(r1)
      e(0+(i_e-1)*3)=r1*2.-1. !can be negative
      counter=0
      do
           counter=counter+1
           call random_number(r2)
           e(1+(i_e-1)*3)=r2*2.-1. !can be negative
           e22=1.-e(0+(i_e-1)*3)**2-e(1+(i_e-1)*3)**2
           if(e22>=0.) go to 90
      end do
      90 continue
     call random_number(r3)
      r3=r3*2.-1. !attribute sign to 3d conmponent in random way
      if(r3>=0.) then
      e(2+(i_e-1)*3)=sqrt(e22)
      else if(r3<0.) then
```

```
    e(2+(i_e-1)*3)=-sqrt(e22) !can be negative
    end if

    end do !i_e

    deallocate(seed)

end if

!----------------------------------------




  END SUBROUTINE initializer


END MODULE first_bact
```

```
  !
@@@@@@@@@@@@@@@@@@@@@@@@@@@@@@@@@@@@@@@@@@@@@@@@@@@@@@@@@@@@@@@@@@@@
@@@@@@@@@@@@@@@@@@@@@@@@@@@@
  !-----------------------------------------------------------------
------------------------------
  !@@@@@@@@@@@@@@@@@@@@@@@@@@     obtain times from an exponential
distribution      @@@@@@@@@@@@@@@@@@@@@@@@
  !@@@@@@@@@@@@@@@@@@@@@@@@@@  inverse transformation method
@@@@@@@@@@@@@@@@@@@@@@@@@@@@@@@@@@@@@@
  !-----------------------------------------------------------------
-------------------------------
  !
@@@@@@@@@@@@@@@@@@@@@@@@@@@@@@@@@@@@@@@@@@@@@@@@@@@@@@@@@@@@@@@@@@@@
@@@@@@@@@@@@@@@@@@@@@@@@@@@@
  !@@@@@@@@@ AUTHOR: CODUTTI AGNESE
  !@@@@@@@@@ LAST MODIFIED: 05.11.2015
  !@@@@@@@@@ credits: numerical recipes for fortran chap.7 random
numbers pag 280




MODULE second_bact
IMPLICIT NONE
   integer, parameter:: sp = kind(1.d0)
   private:: sp


  CONTAINS


  SUBROUTINE time_sel(t_medio,time_outcome)
  implicit none

  real(kind=sp), intent(in) :: t_medio                         !mean
time of the exponential distribution
  real(kind=sp), intent(out) :: time_outcome                   !time
from exponential distribution
  real(kind=sp) :: r

    do
       call random_number(r)
       if(r > 0) exit
    end do
    time_outcome = -t_medio*log(r)






  END SUBROUTINE time_sel
```

```
END MODULE second_bact
```

```
  !
@@@@@@@@@@@@@@@@@@@@@@@@@@@@@@@@@@@@@@@@@@@@@@@@@@@@@@@@@@@@@@@@@@@@
@@@@@@@@@@@@@@@@@@@@@@@@@@@@
  !-----------------------------------------------------------------
------------------------------
  !@@@@@@@@@@@@@@@@@@@@@@@@@@     integration of the equations
@@@@@@@@@@@@@@@@@
  !-----------------------------------------------------------------
-------------------------------
  !
@@@@@@@@@@@@@@@@@@@@@@@@@@@@@@@@@@@@@@@@@@@@@@@@@@@@@@@@@@@@@@@@@@@@
@@@@@@@@@@@@@@@@@@@@@@@@@@@@
  !@@@@@@@@@ AUTHOR: CODUTTI AGNESE
  !@@@@@@@@@ LAST MODIFIED: 10.12.2015




MODULE third_bact
use fourth_bact                                  !contains
box_muller
implicit none
   integer, parameter:: sp = kind(1.d0)
   private:: sp
  CONTAINS







  !
@@@@@@@@@@@@@@@@@@@@@@@@@@@@@@@@@@@@@@@@@@@@@@@@@@@@@@@@@@@@@@@@@@@@
@@@@@@@@@@@@@@@@@@@@@@@@@@@@@
  !@@@@@@@@@@@@@@@@@@@@@@@@@@ EQUATIONS RUN/TUMBLE
@@@@@@@@@@@@@@@@@@@@@@@@@@@@@@@@@@@@@@@@@@@@@@@@@
  !
@@@@@@@@@@@@@@@@@@@@@@@@@@@@@@@@@@@@@@@@@@@@@@@@@@@@@@@@@@@@@@@@@@@@
@@@@@@@@@@@@@@@@@@@@@@@@@@@@@

  SUBROUTINE
equations(filechoice,bact_status,D,B,M,gamma_t,gamma_r,v_self,T,Teff
,x,e,deltat,&

direction,filename,reversechoice,F,i_time)
  implicit none
        character(len=1),intent(in)::bact_status
        integer :: s
        integer,intent(in):: i_time
        integer, intent(in) :: D
        REAL(kind=sp),intent(in) :: gamma_r,gamma_t
        REAL(kind=sp), DIMENSION(0:2),intent(inout):: x,e
```

```
        REAL(kind=sp), intent(in) :: deltat,v_self
        REAL(kind=sp), intent(in):: T, Teff
        real(kind=sp),intent(in) :: M
        real(kind=sp),dimension(0:2), intent(in) :: B

        REAL(kind=sp), DIMENSION(0:2):: delta_phi, delta_e,dx,v
        REAL(kind=sp), DIMENSION(0:2):: delta_w1, delta_w2
        integer :: i,k,j,l
        real(kind=sp) :: Temperatura_tumble
        real(kind=sp) :: psi!,vtumble
        logical :: exist
        real(kind=sp) :: mod_e_single_bact
        real(kind=sp),parameter :: Kb=1.3806488*10**(-11.) !
microm**2 kg s**(-2) K**(-1)  Boltzmann const.
        REAL(kind=sp), DIMENSION(0:2)::
prodeB                       !it will contain the cross product e X
B
        real(kind=sp) :: cosrelax
        real(kind=sp),parameter :: pi=3.1415926535897932
        integer,dimension(6),intent(in) :: filechoice
        real(kind=sp),parameter:: cut=1.*10**(-3.) !sigma from relax
angle distribution at steady state with B=50

        integer, intent(in):: direction
        character(len=100),intent(in) :: filename
        integer,intent(in) :: reversechoice
        real(kind=sp),dimension(0:2),intent(in) :: F


!@@@@@@@@@@@@@@@@@@@@@@@@@@@@@@@@@@@files for saving
data@@@@@@@@@@@@@@@@@@@@@@@@@@@@@@@@@@@@@@@@@@@@@@@@@@@@@@@@@@@@@@@@@@@@@@
@@@@@@@@@@@@@@@@@@@@@@@@@@@

     if(filechoice(4)==1)  then
          inquire(file=filename, exist=exist)
           if (exist) then
             open(10, file=filename, status="old",
position="append", action="write")
           else
             open(10, file=filename, status="new", action ="write")
           end if
     end if




     if(filechoice(5)==1) then
           inquire(file="vel.dat", exist=exist)
           if (exist) then
             open(12, file="vel.dat", status="old",
position="append", action="write")
           else
             open(12, file="vel.dat", status="new", action="write")
           end if
```

```
     end if


    if(filechoice(6)==1) then
           inquire(file="relax.dat", exist=exist)
           if (exist) then
             open(30, file="relax.dat", status="old",
position="append", action="write")
           else
             open(30, file="relax.dat", status="new",
action="write")
           end if
   end if



!
@@@@@@@@@@@@@@@@@@@@@@@@@@@@@@@@@@@@@@@@@@@@@@@@@@@@@@@@@@@@@@@@@@@@@
@@@@@@@@@@@@@@@@@@@@@@@@@@@@@@@@@@@@@@@@@@@@@@@@@@@@@@@@@@@@@@@@@@@@@
@@@@@@@@@@@@@@@@@@@


!
@@@@@@@@@@@@@@@@@@@@@@@@@@@@@@@@@@@@@@@INIZIALIZZAZIONE@@@@@@@@@@@@@
@@@@@@@@@@@@@@@@@@@@@@@@@@@@@@@@@@@@@@@@@@@@@@@@@@@@@@@@@@@@@@@@@@@@@
@@@@@@@@@@@@@@@@@@@

        !is it a run or a tumble?
        if(bact_status=='r') s=1
        if(bact_status=='c') s=2



        !initialization
        psi=0.

        delta_phi=0.
        mod_e_single_bact=0.
        delta_e=0.
        delta_w1=0.
        delta_w2=0.




       if(reversechoice==0) then !tumble and not reverse
        if(s==1) then
          Temperatura_tumble=T !room temperature
        else if(s==2) then
          Temperatura_tumble=Teff !effective temperature that
produces the tumbling
        end if
       end if
```

```
      if(reversechoice==1) Temperatura_tumble=T !reverse

!
@@@@@@@@@@@@@@@@@@@@@@@@@@@@@@@@@@@@@@@@@@@@@@@@@@@@@@@@@@@@@@@@@@@@
@@@@@@@@@@@@@@@@@@@@@@@@@@@@@@@@@@@@@@@@@@@@@@@@@@@@@@@@@@@@@@@@@@@@
@@@@@@@@@@@@@@@@@@@

!
@@@@@@@@@@@@@@@@@@@@@@@@@@@@@@@@@@@@@@@@@@@@@@@@@@@@@@@@@@@@@@@@@@@@
@@@@@@@@@@@@@@@@@@@@@@@@@@@@@@@@@@@@@@@@@@@@@@@@@@@@@@@@@@@@@@@@@@@@
@@@@@@@@@@@@@@@@@@@
!@@@@@@@@@@@@@@@@@@@@@@@@@@@@@@@@@@@@@@@@@@@@@@@@@@ INTEGRATION
@@@@@@@@@@@@@@@@@@@@@@@@@@@@@@@@@@@@@@@@@@@@@@@@@@@@@@@@@@@@@@@@@@@@
@@@@@@@@@
!
@@@@@@@@@@@@@@@@@@@@@@@@@@@@@@@@@@@@@@@@@@@@@@@@@@@@@@@@@@@@@@@@@@@@
@@@@@@@@@@@@@@@@@@@@@@@@@@@@@@@@@@@@@@@@@@@@@@@@@@@@@@@@@@@@@@@@@@@@
@@@@@@@@@@@@@@@@@@@

 if(filechoice(4)==1 .and. i_time==1 .and. s==1)
write(unit=10,fmt=*) x,i_time*deltat !initial position is written
only the first time

       !@@@@@@@@@@@@@@@@@@@@@@@@@@@@@@@@@@WIENER PROCESS-
TRASLATIONAL@@@@@@@@@@@@@@@@@@@@@@@@@@@@@@@@@
          do i=0,2
           if(D==2) then
                if(i==2) then
                   delta_w1(i)=0.                       !in 2D the
third component is 0.
                   go to 100
                end if

                call box_muller(psi)
                delta_w1(i)=psi
                100 continue
           else
               call box_muller(psi)
               delta_w1(i)=psi
          end if

        end do
        delta_w1=delta_w1*sqrt(deltat)              !wiener process
has a sort of time
       !
```

```
@@@@@@@@@@@@@@@@@@@@@@@@@@@@@@@@@@@@@@@@@@@@@@@@@@@@@@@@@@@@@@@@@@@@
@@@@@@@@@@@@@@@@@@@@@@@@



       !@@@@@@@@@@@@@@@@@@@@@@@@@@@@@@@@@WIENER PROCESS-
ROTATIONAL@@@@@@@@@@@@@@@@@@@@@@@@@@@@@@@@@@@
        do i=0,2
           if(D==2) then
                if(i==0.or. i==1) then
                   delta_w2(i)=0.
                   go to 200
                end if


                call box_muller(psi)
                delta_w2(i)=psi
                200 continue
           else
               call box_muller(psi)
               delta_w2(i)=psi
          end if
        end do

         delta_w2=delta_w2*sqrt(deltat)
        !
@@@@@@@@@@@@@@@@@@@@@@@@@@@@@@@@@@@@@@@@@@@@@@@@@@@@@@@@@@@@@@@@@@@@
@@@@@@@@@@@@@@@@@@@@@@@@



        !-----------------------------------------------------------
--------------------------------
        !@@@@@@@@@@@@@@@@@@@@@@@@@@@@@@@@@euler maruyama
integration@@@@@@@@@@@@@@@@@@@@@@@@@@@@@@@@
        !
@@@@@@@@@@@@@@@@@@@@@@@@@@@@@@@@@@@@@@@@@@@@@@@@@@@@@@@@@@@@@@@@@@@@
@@@@@@@@@@@@@@@
        !-----------------------------------------------------------
---------------------------------

       !position
       dx=(2-s)*direction*(v_self*deltat*e) +
sqrt(2.*T*Kb*((gamma_t)**(-1)))*delta_w1 + F*deltat*(gamma_t)**(-1)
       x=x+dx
       v=dx/deltat

        !@@@@@@@@@@@@@@@@@@@@@@@@@@@@@@@@@@ angle
@@@@@@@@@@@@@@@@@@@@@@@@@@@@@@@@@@@@@@@@@@@@@@@@@@@@

        prodeB=0.

        prodeB(0)=e(1)*B(2)-e(2)*B(1)
        prodeB(1)=-e(0)*B(2)+e(2)*B(0)
```

```
        prodeB(2)=e(0)*B(1)-e(1)*B(0)


delta_phi=sqrt(2.*Temperatura_tumble*Kb*((gamma_r)**(-1)))*delta_w2
+ M*prodeB*deltat*(gamma_r**(-1.))


        !@@@@@@@@@@@@@@ delta_e=delta_phi X e
@@@@@@@@@@@@@@@@@@@@@@@@@@@@@@@@@@@@@@@@@@@@@@@@@@@@@@@@@


           delta_e(0)=delta_phi(1)*e(2)-delta_phi(2)*e(1)
           delta_e(1)=delta_phi(2)*e(0)-delta_phi(0)*e(2)
           delta_e(2)=delta_phi(0)*e(1)-delta_phi(1)*e(0)



        e=e+delta_e


        !@@@@@@@@@@@@@@@ normalize e for each bacterium
@@@@@@@@@@@@@@@@@@@@@@@@@@@@@@@@@@@@@@@@@@@@@


          mod_e_single_bact=sqrt(e(0)**2+e(1)**2+e(2)**2)

          e(0)=e(0)/mod_e_single_bact
          e(1)=e(1)/mod_e_single_bact
          e(2)=e(2)/mod_e_single_bact





        !
@@@@@@@@@@@@@@@@@@@@@@@@@@@@@@@@@@@@@@@@@@@@@@@@@@@@@@@@@@@@@@@@@@@@
@@@@@@@@@@@@@@@@@@@@@@@@




        ! cosine of angle between e and B, for output
        cosrelax=(B(0)*e(0)+B(1)*e(1)+B(2)*e(2))*direction !
cosrelax=e scalar B
        cosrelax=cosrelax/sqrt(B(0)**2+B(1)**2+B(2)**2)

        if(cosrelax>1.) cosrelax=1.
        if(cosrelax<-1.) cosrelax=-1.



       !5 ->  save every 0.01s, 50-> save every 0.1s if dt=0.002s
```

```
        if(mod(i_time,50)==0) then
           if(filechoice(4)==1)      write(unit=10,fmt=*)
x,i_time*deltat
           if(filechoice(5)==1)     write(unit=12,fmt=*)
v,i_time*deltat
           if(filechoice(6)==1)     write(30,*) i_time*deltat,
cosrelax, acos(cosrelax)

        end if








   if(filechoice(4)==1)     close(10)
   if(filechoice(5)==1)    close(12)
   if(filechoice(6)==1)      close(30)


!
@@@@@@@@@@@@@@@@@@@@@@@@@@@@@@@@@@@@@@@@@@@@@@@@@@@@@@@@@@@@@@@@@@@@
@@@@@@@@@@@@@@@@@@@@@@@@@@@@@@@@@@@@@@@@@@@@@@@@@@@@@@@@@@@@@@@@@@@@
@@@@@@@@@@@@@@@@@@@
!
@@@@@@@@@@@@@@@@@@@@@@@@@@@@@@@@@@@@@@@@@@@@@@@@@@@@@@@@@@@@@@@@@@@@
@@@@@@@@@@@@@@@@@@@@@@@@@@@@@@@@@@@@@@@@@@@@@@@@@@@@@@@@@@@@@@@@@@@@
@@@@@@@@@@@@@@@@@@@

  END SUBROUTINE equations


END MODULE third_bact
```

```
  !
@@@@@@@@@@@@@@@@@@@@@@@@@@@@@@@@@@@@@@@@@@@@@@@@@@@@@@@@@@@@@@@@@@@@
@@@@@@@@@@@@@@@@@@@@@@@@@@@@
  !-----------------------------------------------------------------
------------------------------
  !@@@@@@@@@@@@@@@@@@@     gaussian numbers generator
@@@@@@@@@@@@@@@@@@
  !@@@@@@@@@@@@@@@@@@@    box muller method
@@@@@@@@@@@@@@@@@@@@@@@@@@@@@@@@@@@@@@@@@@@@@@@@@@
  !-----------------------------------------------------------------
-------------------------------
  !
@@@@@@@@@@@@@@@@@@@@@@@@@@@@@@@@@@@@@@@@@@@@@@@@@@@@@@@@@@@@@@@@@@@@
@@@@@@@@@@@@@@@@@@@@@@@@@@@@
  !@@@@@@@@@ AUTHOR: CODUTTI AGNESE
  !@@@@@@@@@ LAST MODIFIED: 05.11.2015
  !@@@@@@@@@ credits: numerical recipes for fortran chap.7 random
numbers pag 280



MODULE fourth_bact
IMPLICIT NONE
   integer, parameter:: sp = kind(1.d0)
   private:: sp

  CONTAINS



  SUBROUTINE box_muller(a)
  implicit none
    real(kind=sp),intent(out):: a
    REAL(kind=sp) :: r2, x, y
    REAL(kind=sp), SAVE :: g
    LOGICAL, SAVE :: gaus_stored=.false.






    if (gaus_stored) then
       a=g
       gaus_stored=.false.
    else
       do
          call random_number(x)   !pseudo random number unif. distr.
between 0 and 1
          call random_number(y)
          x=2.*x-1.               !pseudo random number unif. distr.
between -1 and 1
          y=2.*y-1.
```

```
          r2=x**2+y**2
          if (r2 > 0. .and. r2 < 1.) exit  !r2 is uniformally
distributed between 0 and 1
       end do
       r2=sqrt(-2.*log(r2)/r2)
       a=x*r2
       g=y*r2
       gaus_stored=.true.    !In each cycle I produce 2 random
numbers; second time i call the
    end if                   !function I have the second already
stored and i avoid calculation



  END SUBROUTINE box_muller


END MODULE fourth_bact
```

```
  !
@@@@@@@@@@@@@@@@@@@@@@@@@@@@@@@@@@@@@@@@@@@@@@@@@@@@@@@@@@@@@@@@@@@@
@@@@@@@@@@@@@@@@@@@@@@@@@@@@
  !-----------------------------------------------------------------
------------------------------
  !@@@@@@@@@@@@@@@@@@@@@@@@@@ to decide mean run time when there is
an oxygen gradient @@@@@@@@@@@@
  !@@@@@@@@@@@@@@@@@@@@@@@@@@
  !-----------------------------------------------------------------
-------------------------------
  !
@@@@@@@@@@@@@@@@@@@@@@@@@@@@@@@@@@@@@@@@@@@@@@@@@@@@@@@@@@@@@@@@@@@@
@@@@@@@@@@@@@@@@@@@@@@@@@@@@
  !@@@@@@@@@ AUTHOR: CODUTTI AGNESE
  !@@@@@@@@@ CREATED ON 12.02.2016
  !@@@@@@@@@ LAST MODIFIED: 12.02.2016




MODULE fifth_bact
IMPLICIT NONE
   integer, parameter:: sp = kind(1.d0)
   private:: sp


  CONTAINS



  SUBROUTINE
mean_time_calc(kindofchemotaxis,numpart,e,direction,gradO2_bact,conc
_x,t1_0,grad_ref,t1_medio,cstar,xposition,i_bac)
  implicit none
  integer,intent(in) :: numpart
  real(kind=sp),dimension(0:2),intent(in) :: e
  real(kind=sp),dimension(0:2), intent(in) :: gradO2_bact
  real(kind=sp), intent(in) :: t1_0,grad_ref !grad_ref=25muM/mm
  real(kind=sp),intent(out) :: t1_medio
  real(kind=sp) ::t1_max
  real(kind=sp) :: scalar
  real(kind=sp),intent(in) :: cstar !prefered concentration in
micromolar
  real(kind=sp), intent(in) :: xposition
  integer,intent(in) :: kindofchemotaxis !0==with prefered
concentration, 1=towards maximum attractant, 2=towards minimum
repellent
  real(kind=sp),intent(in):: conc_x
  integer, intent(in):: direction
  logical :: exist
  integer,intent(in):: i_bac
```

```
scalar=(e(0)*gradO2_bact(0)+e(1)*gradO2_bact(1)+e(2)*gradO2_bact(2))
*direction    !scalar product between gradO2 and e*direction=v/|v|;
grad y and z components are systemnatically 0







!@@@@@ towards a defined star value, for band formation
if (kindofchemotaxis==0) then


  !----
  if(conc_x>=cstar) then                                !
concentration above the prefered one

       if(scalar>=0.) then                  !swims towards lowest
conc., so down the gradient
        t1_medio=t1_0*0.9
       else if(scalar<0.) then
        t1_medio=t1_0*2.
       end if

  elseif(conc_x<cstar) then                             !
concentration belowed the prefered one

       if(scalar>0.) then                   !swims towards lowest
conc., so down the gradient
        t1_medio=2.*t1_0
       else if(scalar<=0.) then
        t1_medio=t1_0*0.9
       end if

  end if
  !----


!@@@attractant
else if (kindofchemotaxis==1) then


   if(scalar/grad_ref>1.)then
    t1_medio=2.*t1_0
   else if(0.<scalar/grad_ref .and. scalar/grad_ref<=1.) then
    t1_medio=t1_0*(1+ scalar/grad_ref)            !UP the gradient -
attractant
   else if(scalar/grad_ref<=0.) then
    t1_medio=t1_0
   end if
```

```
!@@@repellent
else if (kindofchemotaxis==2) then

   if(scalar/grad_ref>=0.) then
    t1_medio=t1_0
   else if(-1.<scalar/grad_ref .and. scalar/grad_ref<0.) then
    t1_medio=t1_0*(1- scalar/grad_ref)            !down the gradient
- repellent
   else if(scalar/grad_ref<=-1.) then
    t1_medio=2.*t1_0
   end if



end if
!@@@@@



  END SUBROUTINE mean_time_calc


END MODULE fifth_bact
```

```
  !
@@@@@@@@@@@@@@@@@@@@@@@@@@@@@@@@@@@@@@@@@@@@@@@@@@@@@@@@@@@@@@@@@@@@
@@@@@@@@@@@@@@@@@@@@@@@@@@@@
  !-------------------------------------------------------------------
------------------------------
  !@@@@@@@@@@@@@@@@@@@@    integrate oxygen concentration and
calculate gradient @@@@@@@@@@@@@@@@@@@
  !-------------------------------------------------------------------
-------------------------------
  !
@@@@@@@@@@@@@@@@@@@@@@@@@@@@@@@@@@@@@@@@@@@@@@@@@@@@@@@@@@@@@@@@@@@@
@@@@@@@@@@@@@@@@@@@@@@@@@@@@
  !@@@@@@@@@@ AUTHOR: CODUTTI AGNESE
  !@@@@@@@@@@ LAST MODIFIED: 18.01.2017




MODULE sixth_bact
IMPLICIT NONE
   integer, parameter:: sp = kind(1.d0)
   private:: sp

  CONTAINS


  !
@@@@@@@@@@@@@@@@@@@@@@@@@@@@@@@@@@@@@@@@@@@@@@@@@@@@@@@@@@@@@@@@@@@@
@@@@@@@@@@@@
  SUBROUTINE
calculate_concentration(D,k,ca,O2left,rho,deltax,deltay,deltaz,ndelt
ax,ndeltay,ndeltaz,deltat,conc,&
  diffusion,consumption)
  implicit none
    integer,intent(in):: ndeltax,ndeltay,ndeltaz

integer,dimension(0:ndeltax-1,0:ndeltay-1,0:ndeltaz-1),intent(in)::
rho
    REAL(kind=sp),intent(in) :: deltax,deltay,deltaz,deltat

real(kind=sp),dimension(0:ndeltax-1,0:ndeltay-1,0:ndeltaz-1),intent(
inout):: conc !enetrs old one, goes out new one

real(kind=sp),dimension(1:ndeltax-2,1:ndeltay-2,1:ndeltaz-2),intent(
out):: diffusion,consumption !in bulk only
    integer:: j_x,j_y,j_z
    real(kind=sp),intent(in):: D,k,ca,O2left


   !^^^^^^^^^^^^^^^^^^^ INTEGRATION ^^^^^^^^^^^^^^^^^


    do j_x=1,ndeltax-2
```

```
    do j_y=1,ndeltay-2
    do j_z=1,ndeltaz-2
     conc(j_x,j_y,j_z)=conc(j_x,j_y,j_z)+deltat*((D/deltax**2.)*&
     (conc(j_x+1,j_y,j_z)+conc(j_x-1,j_y,j_z)&
     +conc(j_x,j_y+1,j_z)+conc(j_x,j_y-1,j_z)&
     +conc(j_x,j_y,j_z+1)+conc(j_x,j_y,j_z-1)&
     -6.*conc(j_x,j_y,j_z))&
     -k*conc(j_x,j_y,j_z)*rho(j_x,j_y,j_z)/(conc(j_x,j_y,j_z)+ca)) !
bacteria consumption

     diffusion(j_x,j_y,j_z)=deltat*(D/deltax**2.)*&
     (conc(j_x+1,j_y,j_z)+conc(j_x-1,j_y,j_z)&
     +conc(j_x,j_y+1,j_z)+conc(j_x,j_y-1,j_z)&
     +conc(j_x,j_y,j_z+1)+conc(j_x,j_y,j_z-1)-6.*conc(j_x,j_y,j_z))

     consumption(j_x,j_y,j_z)=-
deltat*k*conc(j_x,j_y,j_z)*rho(j_x,j_y,j_z)/(conc(j_x,j_y,j_z)+ca)
    end do
    end do
    end do

   !^^^^^^^^^^^^^^^^^^^^^^^^^^^^^^^^^^^^^^^^^^^^^^^^^^^



   !^^^^^^^^^^^^^^^^^^^BOUNDARY^^^^^^^^^^^^^^^^^^


   ! ------FACES 6 cases
    do j_x=1,ndeltax-2
    do j_y=1,ndeltay-2

     conc(j_x,j_y,0)=conc(j_x,j_y,0)+deltat*((D/deltax**2.)*&  !time
t-1
     (conc(j_x+1,j_y,0)+conc(j_x-1,j_y,0)&                      !flux
in from x
    + conc(j_x,j_y+1,0)+conc(j_x,j_y-1,0)&                       !
flux in from y
    + conc(j_x,j_y,1)+conc(j_x,j_y,0)&                      !flux in
from z
     -6.*conc(j_x,j_y,0))&                                       !
flux out
     -k*conc(j_x,j_y,0)*rho(j_x,j_y,0)/(conc(j_x,j_y,0)+ca)) !
bacteria consumption

     conc(j_x,j_y,ndeltaz-1)=conc(j_x,j_y,ndeltaz-1)+deltat*((D/
deltax**2.)*&  !time t-1

(conc(j_x+1,j_y,ndeltaz-1)+conc(j_x-1,j_y,ndeltaz-1)&
!flux in from x

+conc(j_x,j_y+1,ndeltaz-1)+conc(j_x,j_y-1,ndeltaz-1)&
!flux in from y
```

```
+conc(j_x,j_y,ndeltaz-1)+conc(j_x,j_y,ndeltaz-2)&
!flux in from z

-6.*conc(j_x,j_y,ndeltaz-1))&
!flux out
     -k*conc(j_x,j_y,ndeltaz-1)*rho(j_x,j_y,ndeltaz-1)/
(conc(j_x,j_y,ndeltaz-1)+ca)) !bacteria consumption
    end do
    end do



    do j_x=1,ndeltax-2
    do j_z=1,ndeltaz-2
     conc(j_x,0,j_z)=conc(j_x,0,j_z)+deltat*((D/deltax**2.)*&  !time
t-1
     (conc(j_x+1,0,j_z)+conc(j_x-1,0,j_z)&                     !flux
in from x
    + conc(j_x,1,j_z)+conc(j_x,0,j_z)&                      !flux in
from y
    + conc(j_x,0,j_z+1)+conc(j_x,0,j_z-1)&                      !
flux in from z
     -6.*conc(j_x,0,j_z))&                                       !
flux out
     -k*conc(j_x,0,j_z)*rho(j_x,0,j_z)/(conc(j_x,0,j_z)+ca)) !
bacteria consumption

          conc(j_x,ndeltay-1,j_z)=conc(j_x,ndeltay-1,j_z)
+deltat*((D/deltax**2.)*&  !time t-1
     (conc(j_x+1,ndeltay-1,j_z)
+conc(j_x-1,ndeltay-1,j_z)&                     !flux in from x
     +conc(j_x,ndeltay-1,j_z)
+conc(j_x,ndeltay-2,j_z)&                     !flux in from y
    +
conc(j_x,ndeltay-1,j_z+1)+conc(j_x,ndeltay-1,j_z-1)&
!flux in from z

-6.*conc(j_x,ndeltay-1,j_z))&
!flux out
     -k*conc(j_x,ndeltay-1,j_z)*rho(j_x,ndeltay-1,j_z)/
(conc(j_x,ndeltay-1,j_z)+ca)) !bacteria consumption
    end do
    end do

    do j_y=1,ndeltay-2
    do j_z=1,ndeltaz-2
     conc(0,j_y,j_z)=conc(0,j_y,j_z)+deltat*((D/deltax**2.)*&  !time
t-1
     (conc(1,j_y,j_z)+O2left&                    !flux in from x
    + conc(0,j_y+1,j_z)+conc(0,j_y-1,j_z)&                      !
flux in from y
    + conc(0,j_y,j_z+1)+conc(0,j_y,j_z-1)&                      !
flux in from z
     -6.*conc(0,j_y,j_z))&                                       !
flux out
```

```
     -k*conc(0,j_y,j_z)*rho(0,j_y,j_z)/(conc(0,j_y,j_z)+ca)) !
bacteria consumption

     conc(ndeltax-1,j_y,j_z)=conc(ndeltax-1,j_y,j_z)+deltat*((D/
deltax**2.)*&  !time t-1
     (conc(ndeltax-1,j_y,j_z)
+conc(ndeltax-2,j_y,j_z)&                    !flux in from x
    + conc(ndeltax-1,j_y+1,j_z)
+conc(ndeltax-1,j_y-1,j_z)&                      !flux in from y

+conc(ndeltax-1,j_y,j_z+1)+conc(ndeltax-1,j_y,j_z-1)&
!flux in from z

-6.*conc(ndeltax-1,j_y,j_z))&
!flux out
     -k*conc(ndeltax-1,j_y,j_z)*rho(ndeltax-1,j_y,j_z)/
(conc(ndeltax-1,j_y,j_z)+ca)) !bacteria consumption
    end do
    end do




   ! ------RIM  12 cases

    do j_x=1,ndeltax-2


     conc(j_x,0,0)=conc(j_x,0,0)+deltat*((D/deltax**2.)*&  !time t-1
     (conc(j_x+1,0,0)+conc(j_x-1,0,0)&                     !flux in
from x
    + conc(j_x,1,0)+conc(j_x,0,0)&                      !flux in
from y
    + conc(j_x,0,1)+conc(j_x,0,0)&                      !flux in
from z
     -6.*conc(j_x,0,0))&                                      !flux
out
     -k*conc(j_x,0,0)*rho(j_x,0,0)/(conc(j_x,0,0)+ca)) !bacteria
consumption

          conc(j_x,0,ndeltaz-1)=conc(j_x,0,ndeltaz-1)+deltat*((D/
deltax**2.)*&  !time t-1

(conc(j_x+1,0,ndeltaz-1)+conc(j_x-1,0,ndeltaz-1)&
!flux in from x
     +conc(j_x,1,ndeltaz-1)+conc(j_x,
0,ndeltaz-1)&                     !flux in from y
     +conc(j_x,0,ndeltaz-1)+conc(j_x,
0,ndeltaz-2)&                     !flux in from z
     -6.*conc(j_x,
0,ndeltaz-1))&                                       !flux out
     -k*conc(j_x,0,ndeltaz-1)*rho(j_x,0,ndeltaz-1)/(conc(j_x,
0,ndeltaz-1)+ca)) !bacteria consumption
     !done
```

```
          conc(j_x,ndeltay-1,0)=conc(j_x,ndeltay-1,0)+deltat*((D/
deltax**2.)*&  !time t-1

(conc(j_x+1,ndeltay-1,0)+conc(j_x-1,ndeltay-1,0)&
!flux in from x
    +
conc(j_x,ndeltay-1,0)+conc(j_x,ndeltay-2,0)&                      !
flux in from y
    +
conc(j_x,ndeltay-1,1)+conc(j_x,ndeltay-1,0)&                      !
flux in from z

-6.*conc(j_x,ndeltay-1,0))&                                       !
flux out
     -k*conc(j_x,ndeltay-1,0)*rho(j_x,ndeltay-1,0)/
(conc(j_x,ndeltay-1,0)+ca)) !bacteria consumption
     !done

conc(j_x,ndeltay-1,ndeltaz-1)=conc(j_x,ndeltay-1,ndeltaz-1)+deltat*(
(D/deltax**2.)*&  !time t-1

(conc(j_x+1,ndeltay-1,ndeltaz-1)+conc(j_x-1,ndeltay-1,ndeltaz-1)&
!flux in from x

+conc(j_x,ndeltay-1,ndeltaz-1)+conc(j_x,ndeltay-2,ndeltaz-1)&
!flux in from y
    +
conc(j_x,ndeltay-1,ndeltaz-1)+conc(j_x,ndeltay-1,ndeltaz-2)&
!flux in from z

-6.*conc(j_x,ndeltay-1,ndeltaz-1))&
!flux out
     -k*conc(j_x,ndeltay-1,ndeltaz-1)*rho(j_x,ndeltay-1,ndeltaz-1)/
(conc(j_x,ndeltay-1,ndeltaz-1)+ca)) !bacteria consumption


    end do

   do j_y=1,ndeltay-2
        conc(0,j_y,0)=conc(0,j_y,0)+deltat*((D/deltax**2.)*&  !time
t-1
     (conc(1,j_y,0)+O2left&                    !flux in from x
    + conc(0,j_y+1,0)+conc(0,j_y-1,0)&                      !flux in
from y
    + conc(0,j_y,1)+conc(0,j_y,0)&                      !flux in
from z
     -6.*conc(0,j_y,0))&                                    !flux
out
     -k*conc(0,j_y,0)*rho(0,j_y,0)/(conc(0,j_y,0)+ca)) !bacteria
consumption

          conc(ndeltax-1,j_y,0)=conc(ndeltax-1,j_y,0)+deltat*((D/
deltax**2.)*&  !time t-1
     (conc(ndeltax-1,j_y,0)+conc(ndeltax-2,j_y,
```

```
0)&                     !flux in from x
    +
conc(ndeltax-1,j_y+1,0)+conc(ndeltax-1,j_y-1,0)&
!flux in from y
    + conc(ndeltax-1,j_y,1)+conc(ndeltax-1,j_y,
0)&                      !flux in from z
     -6.*conc(ndeltax-1,j_y,
0))&                                        !flux out
     -k*conc(ndeltax-1,j_y,0)*rho(ndeltax-1,j_y,0)/
(conc(ndeltax-1,j_y,0)+ca)) !bacteria consumption

          conc(0,j_y,ndeltaz-1)=conc(0,j_y,ndeltaz-1)+deltat*((D/
deltax**2.)*&  !time t-1
     (conc(1,j_y,ndeltaz-1)+O2left&                    !flux in
from x
    +
conc(0,j_y+1,ndeltaz-1)+conc(0,j_y-1,ndeltaz-1)&
!flux in from y
    +
conc(0,j_y,ndeltaz-1)+conc(0,j_y,ndeltaz-2)&                      !
flux in from z

-6.*conc(0,j_y,ndeltaz-1))&                                       !
flux out
     -k*conc(0,j_y,ndeltaz-1)*rho(0,j_y,ndeltaz-1)/
(conc(0,j_y,ndeltaz-1)+ca)) !bacteria consumption


conc(ndeltax-1,j_y,ndeltaz-1)=conc(ndeltax-1,j_y,ndeltaz-1)+deltat*(
(D/deltax**2.)*&  !time t-1

(conc(ndeltax-1,j_y,ndeltaz-1)+conc(ndeltax-2,j_y,ndeltaz-1)&
!flux in from x
   +
conc(ndeltax-1,j_y+1,ndeltaz-1)+conc(ndeltax-1,j_y-1,ndeltaz-1)&
!flux in from y
   +
conc(ndeltax-1,j_y,ndeltaz-1)+conc(ndeltax-1,j_y,ndeltaz-2)&
!flux in from z

-6.*conc(ndeltax-1,j_y,ndeltaz-1))&
!flux out
     -k*conc(ndeltax-1,j_y,ndeltaz-1)*rho(ndeltax-1,j_y,ndeltaz-1)/
(conc(ndeltax-1,j_y,ndeltaz-1)+ca)) !bacteria consumption
   end do

     do j_z=1,ndeltaz-2
          conc(0,0,j_z)=conc(0,0,j_z)+deltat*((D/deltax**2.)*&  !
time t-1
     (conc(1,0,j_z)+O2left&                     !flux in from x
    + conc(0,1,j_z)+conc(0,0,j_z)&                      !flux in
from y
    + conc(0,0,j_z+1)+conc(0,0,j_z-1)&                      !flux in
from z
```

```
     -6.*conc(0,0,j_z))&                                       !flux
out
     -k*conc(0,0,j_z)*rho(0,0,j_z)/(conc(0,0,j_z)+ca)) !bacteria
consumption

          conc(ndeltax-1,0,j_z)=conc(ndeltax-1,0,j_z)+deltat*((D/
deltax**2.)*&  !time t-1
     (conc(ndeltax-1,0,j_z)
+conc(ndeltax-2,0,j_z)&                     !flux in from x
    + conc(ndeltax-1,1,j_z)
+conc(ndeltax-1,0,j_z)&                      !flux in from y
    +
conc(ndeltax-1,0,j_z+1)+conc(ndeltax-1,0,j_z-1)&
!flux in from z

-6.*conc(ndeltax-1,0,j_z))&                                       !
flux out
     -k*conc(ndeltax-1,0,j_z)*rho(ndeltax-1,0,j_z)/
(conc(ndeltax-1,0,j_z)+ca)) !bacteria consumption
     !done
          conc(0,ndeltay-1,j_z)=conc(0,ndeltay-1,j_z)+deltat*((D/
deltax**2.)*&  !time t-1
     (conc(1,ndeltay-1,j_z)+O2left&                    !flux in
from x
    + conc(0,ndeltay-1,j_z)
+conc(0,ndeltay-2,j_z)&                      !flux in from y
    +
conc(0,ndeltay-1,j_z+1)+conc(0,ndeltay-1,j_z-1)&
!flux in from z

-6.*conc(0,ndeltay-1,j_z))&                                       !
flux out
     -k*conc(0,ndeltay-1,j_z)*rho(0,ndeltay-1,j_z)/
(conc(0,ndeltay-1,j_z)+ca)) !bacteria consumption


conc(ndeltax-1,ndeltay-1,j_z)=conc(ndeltax-1,ndeltay-1,j_z)
+deltat*((D/deltax**2.)*&  !time t-1
     (conc(ndeltax-1,ndeltay-1,j_z)
+conc(ndeltax-2,ndeltay-1,j_z)&                    !flux in from x
    + conc(ndeltax-1,ndeltay-1,j_z)
+conc(ndeltax-1,ndeltay-2,j_z)&                     !flux in from y
    +
conc(ndeltax-1,ndeltay-1,j_z+1)+conc(ndeltax-1,ndeltay-1,j_z-1)&
!flux in from z

-6.*conc(ndeltax-1,ndeltay-1,j_z))&
!flux out
     -k*conc(ndeltax-1,ndeltay-1,j_z)*rho(ndeltax-1,ndeltay-1,j_z)/
(conc(ndeltax-1,ndeltay-1,j_z)+ca)) !bacteria consumption
     end do
```

```
   ! ------CORNERS 8 cases
      !0,0,0
        conc(0,0,0)=conc(0,0,0)+deltat*((D/deltax**2.)*&  !time t-1
     (conc(1,0,0)+O2left&                      !flux in from x
    + conc(0,1,0)+conc(0,0,0)&                       !flux in from y
    + conc(0,0,1)+conc(0,0,0)&                       !flux in from z
     -6.*conc(0,0,0))&                                         !flux
out
     -k*conc(0,0,0)*rho(0,0,0)/(conc(0,0,0)+ca)) !bacteria
consumption

     !L,0,0
          conc(ndeltax-1,0,0)=conc(ndeltax-1,0,0)+deltat*((D/
deltax**2.)*&  !time t-1
     (conc(ndeltax-1,0,0)+conc(ndeltax-2,0,0)&                    !
flux in from x
    +
conc(ndeltax-1,1,0)+conc(ndeltax-1,0,0)&                      !flux
in from y
    +
conc(ndeltax-1,0,1)+conc(ndeltax-1,0,0)&                      !flux
in from z

-6.*conc(ndeltax-1,0,0))&                                        !
flux out
     -k*conc(ndeltax-1,0,0)*rho(ndeltax-1,0,0)/
(conc(ndeltax-1,0,0)+ca)) !bacteria consumption

     !0,L,0
          conc(0,ndeltay-1,0)=conc(0,ndeltay-1,0)+deltat*((D/
deltax**2.)*&  !time t-1
     (conc(1,ndeltay-1,0)+O2left&                    !flux in from
x
    +
conc(0,ndeltay-1,0)+conc(0,ndeltay-2,0)&                      !flux
in from y
   +
conc(0,ndeltay-1,1)+conc(0,ndeltay-1,0)&                      !flux
in from z

-6.*conc(0,ndeltay-1,0))&                                        !
flux out
     -k*conc(0,ndeltay-1,0)*rho(0,ndeltay-1,0)/
(conc(0,ndeltay-1,0)+ca)) !bacteria consumption

     !0,0,L
          conc(0,0,ndeltaz-1)=conc(0,0,ndeltaz-1)+deltat*((D/
deltax**2.)*&  !time t-1
     (conc(1,0,ndeltaz-1)+O2left&                    !flux in from
x
    +
conc(0,1,ndeltaz-1)+conc(0,0,ndeltaz-1)&                      !flux
in from y
    +
```

```
conc(0,0,ndeltaz-1)+conc(0,0,ndeltaz-2)&                      !flux
in from z

-6.*conc(0,0,ndeltaz-1))&                                        !
flux out
     -k*conc(0,0,ndeltaz-1)*rho(0,0,ndeltaz-1)/
(conc(0,0,ndeltaz-1)+ca)) !bacteria consumption


     !L,L,0

conc(ndeltax-1,ndeltay-1,0)=conc(ndeltax-1,ndeltay-1,0)+deltat*((D/
deltax**2.)*&  !time t-1

(conc(ndeltax-1,ndeltay-1,0)+conc(ndeltax-2,ndeltay-1,0)&
!flux in from x
    +
conc(ndeltax-1,ndeltay-1,0)+conc(ndeltax-1,ndeltay-2,0)&
!flux in from y
    +
conc(ndeltax-1,ndeltay-1,1)+conc(ndeltax-1,ndeltay-1,0)&
!flux in from z

-6.*conc(ndeltax-1,ndeltay-1,0))&
!flux out
     -k*conc(ndeltax-1,ndeltay-1,0)*rho(ndeltax-1,ndeltay-1,0)/
(conc(ndeltax-1,ndeltay-1,0)+ca)) !bacteria consumption


     !L,0,L

conc(ndeltax-1,0,ndeltaz-1)=conc(ndeltax-1,0,ndeltaz-1)+deltat*((D/
deltax**2.)*&  !time t-1

(conc(ndeltax-1,0,ndeltaz-1)+conc(ndeltax-2,0,ndeltaz-1)&
!flux in frome(0)*gradO2_bact(0) x
    +
conc(ndeltax-1,1,ndeltaz-1)+conc(ndeltax-1,0,ndeltaz-1)&
!flux in from y
    +
conc(ndeltax-1,0,ndeltaz-1)+conc(ndeltax-1,0,ndeltaz-2)&
!flux in from z

-6.*conc(ndeltax-1,0,ndeltaz-1))&
!flux out
     -k*conc(ndeltax-1,0,ndeltaz-1)*rho(ndeltax-1,0,ndeltaz-1)/
(conc(ndeltax-1,0,ndeltaz-1)+ca)) !bacteria consumption

     !L,L,L

conc(ndeltax-1,ndeltay-1,ndeltaz-1)=conc(ndeltax-1,ndeltay-1,ndeltaz
-1)+deltat*((D/deltax**2.)*&  !time t-1

(conc(ndeltax-1,ndeltay-1,ndeltaz-1)+conc(ndeltax-2,ndeltay-1,ndelta
```

```
z-1)&                     !flux in from x
    +
conc(ndeltax-1,ndeltay-1,ndeltaz-1)+conc(ndeltax-1,ndeltay-2,ndeltaz
-1)&                      !flux in from y
    +
conc(ndeltax-1,ndeltay-1,ndeltaz-1)+conc(ndeltax-1,ndeltay-1,ndeltaz
-2)&                      !flux in from z

-6.*conc(ndeltax-1,ndeltay-1,ndeltaz-1))&
!flux out
     -
k*conc(ndeltax-1,ndeltay-1,ndeltaz-1)*rho(ndeltax-1,ndeltay-1,ndelta
z-1)/(conc(ndeltax-1,ndeltay-1,ndeltaz-1)+ca)) !bacteria consumption

     !0,L,L

conc(0,ndeltay-1,ndeltaz-1)=conc(0,ndeltay-1,ndeltaz-1)+deltat*((D/
deltax**2.)*&  !time t-1
     (conc(1,ndeltay-1,ndeltaz-1)+O2left&                    !flux
in from x
    +
conc(0,ndeltay-1,ndeltaz-1)+conc(0,ndeltay-2,ndeltaz-1)&
!flux in from y
    +
conc(0,ndeltay-1,ndeltaz-1)+conc(0,ndeltay-1,ndeltaz-2)&
!flux in from z

-6.*conc(0,ndeltay-1,ndeltaz-1))&
!flux out
     -k*conc(0,ndeltay-1,ndeltaz-1)*rho(0,ndeltay-1,ndeltaz-1)/
(conc(0,ndeltay-1,ndeltaz-1)+ca)) !bacteria consumption




  !^^^^^^^^^^^^^^^^^^^^^^^^^^^^^^^^^^^^^^^^^^^^^^^



   !controll
   do j_x=0,ndeltax-1
   do j_y=0,ndeltay-1
   do j_z=0,ndeltaz-1
    if(conc(j_x,j_y,j_z)<0) then
       print*, 'killed by negative concentration'
       stop
    end if
  end do
  end do
  end do
  END SUBROUTINE calculate_concentration
  !
```

```
@@@@@@@@@@@@@@@@@@@@@@@@@@@@@@@@@@@@@@@@@@@@@@@@@@@@@@@@@@@@@@@@@@@@
@@@@@@@@@@@@
```

```
  !
@@@@@@@@@@@@@@@@@@@@@@@@@@@@@@@@@@@@@@@@@@@@@@@@@@@@@@@@@@@@@@@@@@@@
@@@@@@@@@@@@
  SUBROUTINE
calculate_gradient(deltax,deltay,deltaz,ndeltax,ndeltay,ndeltaz,conc
,gradO2x,gradO2y,gradO2z)
  implicit none
     integer,intent(in):: ndeltax,ndeltay,ndeltaz
     REAL(kind=sp),intent(in) :: deltax,deltay,deltaz

real(kind=sp),dimension(0:ndeltax-1,0:ndeltay-1,0:ndeltaz-1),intent(
in):: conc

real(kind=sp),dimension(0:ndeltax-1,0:ndeltay-1,0:ndeltaz-1),intent(
out):: gradO2x,gradO2y,gradO2z
     integer:: j_x,j_y,j_z


     do j_x=0,ndeltax-2
      do j_y=0,ndeltay-2
       do j_z=0,ndeltaz-2
         gradO2x(j_x,j_y,j_z)=(conc(j_x+1,j_y,j_z)-
conc(j_x,j_y,j_z))/deltax
          gradO2y(j_x,j_y,j_z)=(conc(j_x,j_y+1,j_z)-
conc(j_x,j_y,j_z))/deltay
           gradO2z(j_x,j_y,j_z)=(conc(j_x,j_y,j_z+1)-
conc(j_x,j_y,j_z))/deltaz
     end do
     end do
     end do
     !done
    !^^^^^^^^^^^^^^^^^^^BOUNDARY^^^^^^^^^^^^^^^^^^


    do j_x=0,ndeltax-2
      do j_y=0,ndeltay-2
         gradO2x(j_x,j_y,ndeltaz-1)=(conc(j_x+1,j_y,ndeltaz-1)-
conc(j_x,j_y,ndeltaz-1))/deltax
```

```
          gradO2y(j_x,j_y,ndeltaz-1)=(conc(j_x,j_y+1,ndeltaz-1)-
conc(j_x,j_y,ndeltaz-1))/deltay
           gradO2z(j_x,j_y,ndeltaz-1)=0.!(conc(j_x,j_y,ndeltaz)-
conc(j_x,j_y,ndeltaz))/deltaz
     end do
     end do




         do j_x=0,ndeltax-2
       do j_z=0,ndeltaz-2
         gradO2x(j_x,ndeltay-1,j_z)=(conc(j_x+1,ndeltay-1,j_z)-
conc(j_x,ndeltay-1,j_z))/deltax
          gradO2y(j_x,ndeltay-1,j_z)=0.!(conc(j_x,ndeltay,j_z)-
conc(j_x,ndeltay,j_z))/deltay
           gradO2z(j_x,ndeltay-1,j_z)=(conc(j_x,ndeltay-1,j_z+1)-
conc(j_x,ndeltay-1,j_z))/deltaz
     end do
     end do


      do j_y=0,ndeltay-2
       do j_z=0,ndeltaz-2
         gradO2x(ndeltax-1,j_y,j_z)=0.!(conc(ndeltax,j_y,j_z)-
conc(ndeltax,j_y,j_z))/deltax
          gradO2y(ndeltax-1,j_y,j_z)=(conc(ndeltax-1,j_y+1,j_z)-
conc(ndeltax-1,j_y,j_z))/deltay
           gradO2z(ndeltax-1,j_y,j_z)=(conc(ndeltax-1,j_y,j_z+1)-
conc(ndeltax-1,j_y,j_z))/deltaz
     end do
     end do

    do j_x=0,ndeltax-2

gradO2x(j_x,ndeltay-1,ndeltaz-1)=(conc(j_x+1,ndeltay-1,ndeltaz-1)-
conc(j_x,ndeltay-1,ndeltaz-1))/deltax
          gradO2y(j_x,ndeltay-1,ndeltaz-1)=0.
           gradO2z(j_x,ndeltay-1,ndeltaz-1)=0.!
(conc(j_x,j_y,ndeltaz)-conc(j_x,j_y,ndeltaz))/deltaz
     end do



       do j_z=0,ndeltaz-2
         gradO2x(ndeltax-1,ndeltay-1,j_z)=0.
          gradO2y(ndeltax-1,ndeltay-1,j_z)=0.!
(conc(j_x,ndeltay,j_z)-conc(j_x,ndeltay,j_z))/deltay

gradO2z(ndeltax-1,ndeltay-1,j_z)=(conc(ndeltax-1,ndeltay-1,j_z+1)-
conc(ndeltax-1,ndeltay-1,j_z))/deltaz
     end do

           do j_y=0,ndeltay-2
         gradO2x(ndeltax-1,j_y,ndeltaz-1)=0.!(conc(ndeltax,j_y,j_z)-
```

```
conc(ndeltax,j_y,j_z))/deltax

gradO2y(ndeltax-1,j_y,ndeltaz-1)=(conc(ndeltax-1,j_y+1,ndeltaz-1)-
conc(ndeltax-1,j_y,ndeltaz-1))/deltay
           gradO2z(ndeltax-1,j_y,ndeltaz-1)=0.!
(conc(ndeltax,j_y,j_z+1)-conc(ndeltax,j_y,j_z))/deltaz
     end do


             gradO2x(ndeltax-1,ndeltay-1,ndeltaz-1)=0!
             gradO2y(ndeltax-1,ndeltay-1,ndeltaz-1)=0.
           gradO2z(ndeltax-1,ndeltay-1,ndeltaz-1)=0.!
(conc(j_x,j_y,ndeltaz)-conc(j_x,j_y,ndeltaz))/deltaz

  END SUBROUTINE calculate_gradient
  !
@@@@@@@@@@@@@@@@@@@@@@@@@@@@@@@@@@@@@@@@@@@@@@@@@@@@@@@@@@@@@@@@@@@@
@@@@@@@@@@@@


END MODULE sixth_bact
```
