# Supplementary figures and images for: Chemotaxis in external fields: Simulations for active magnetic biological matter
Source: PLoS Comput Biol. 2019 Dec 19;15(12):e1007548. doi: 10.1371/journal.pcbi.1007548 (PMC6941824; doi:10.1371/journal.pcbi.1007548)

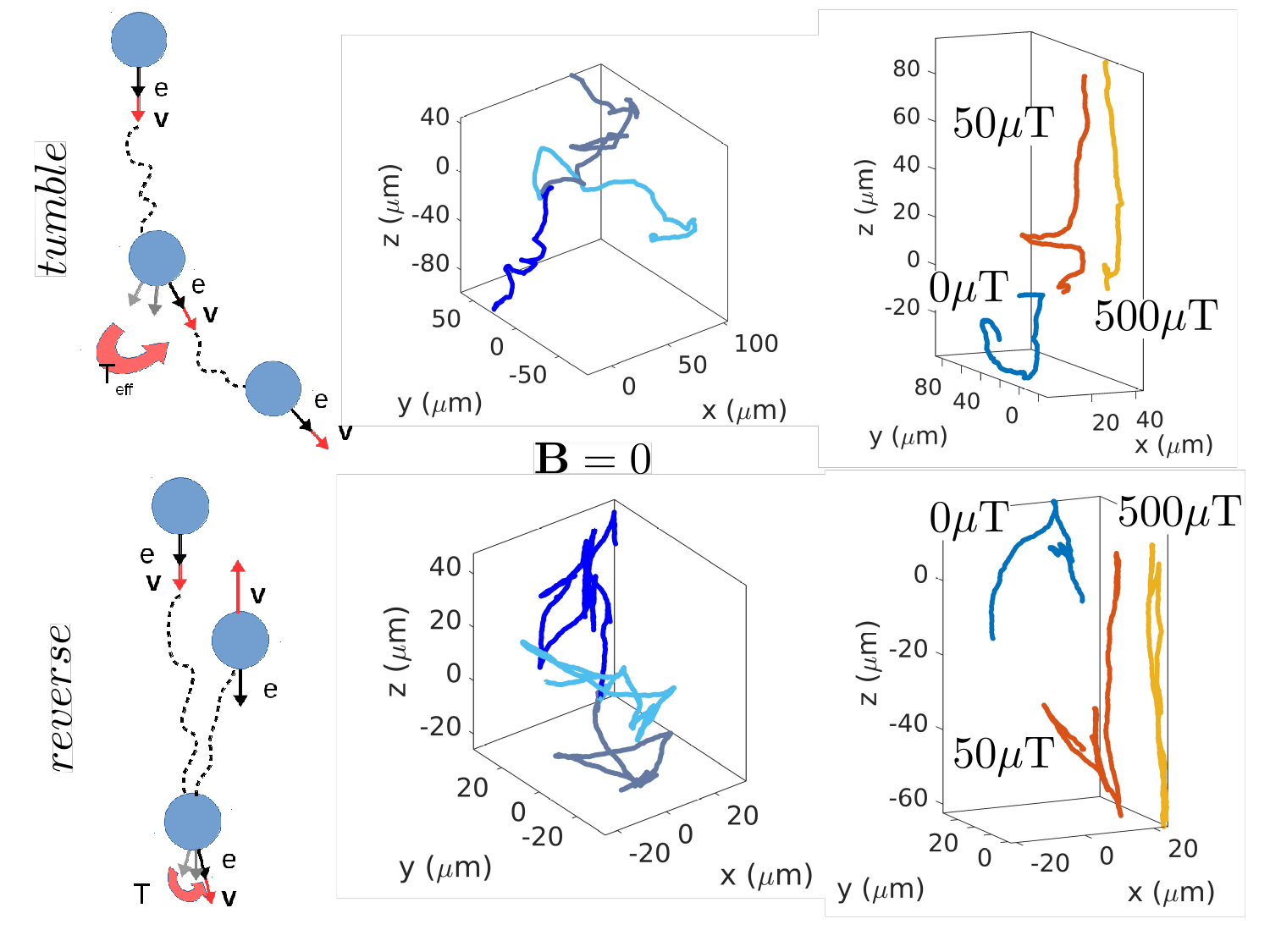

Supplement: S1 Fig — The left column shows a cartoon of the mechanism for the change of direction. The change of direction of bacteria can be performed in different ways. Here in this work we considered tumble (upper row) or reverse (lower row). For example, E. coli performs tumbles with a mean angle of 68° [28]. In our model, tumbling is implemented as rotational diffusion with a high noise strength, which is chosen such as to match this mean angle. This matching is described in S2 Fig. Other bacteria perform run and reverse motion. During a reversal, the body of the bacteria does not re-orient but the bacteria just inverts the direction of velocity. The mean angle is close to 180°, but no exactly, because of thermal noise. In our model, reverse is implemented as a pause mimicking the slowing of motion during a reversal, so there is a time window without active propulsion, during which rotational diffusion due to thermal noise can reorient the cells direction of motion. The middle column shows three example trajectories for run and tumble and for run and reverse, in absence of external forces, torques or gradients. Both run and reverse and run and tumble nicely explore the three dimensional space thanks to thermal noise and thanks to the active changes of direction. A magnetic field provides a torque aligning the direction of motion with the direction of the field and thus, when the field is turned on, the observed behavior changes. Some examples trajectories in the presence of the magnetic field are shown in the right column (blue is without field, red for B = 50 μT -corresponding to the magnetic field of the Earth-, and yellow for B = 500 μT. The field is directed along +z^). For run and tumble, the trajectories are stretched in the direction of the magnetic field and motion is unidirectional parallel to the field, with excursions away from that direction. These are due to the tumbles that kick the trajectories out of alignment, followed by re-alignment during the runs, provided thos [file pcbi.1007548.s006.tiff]

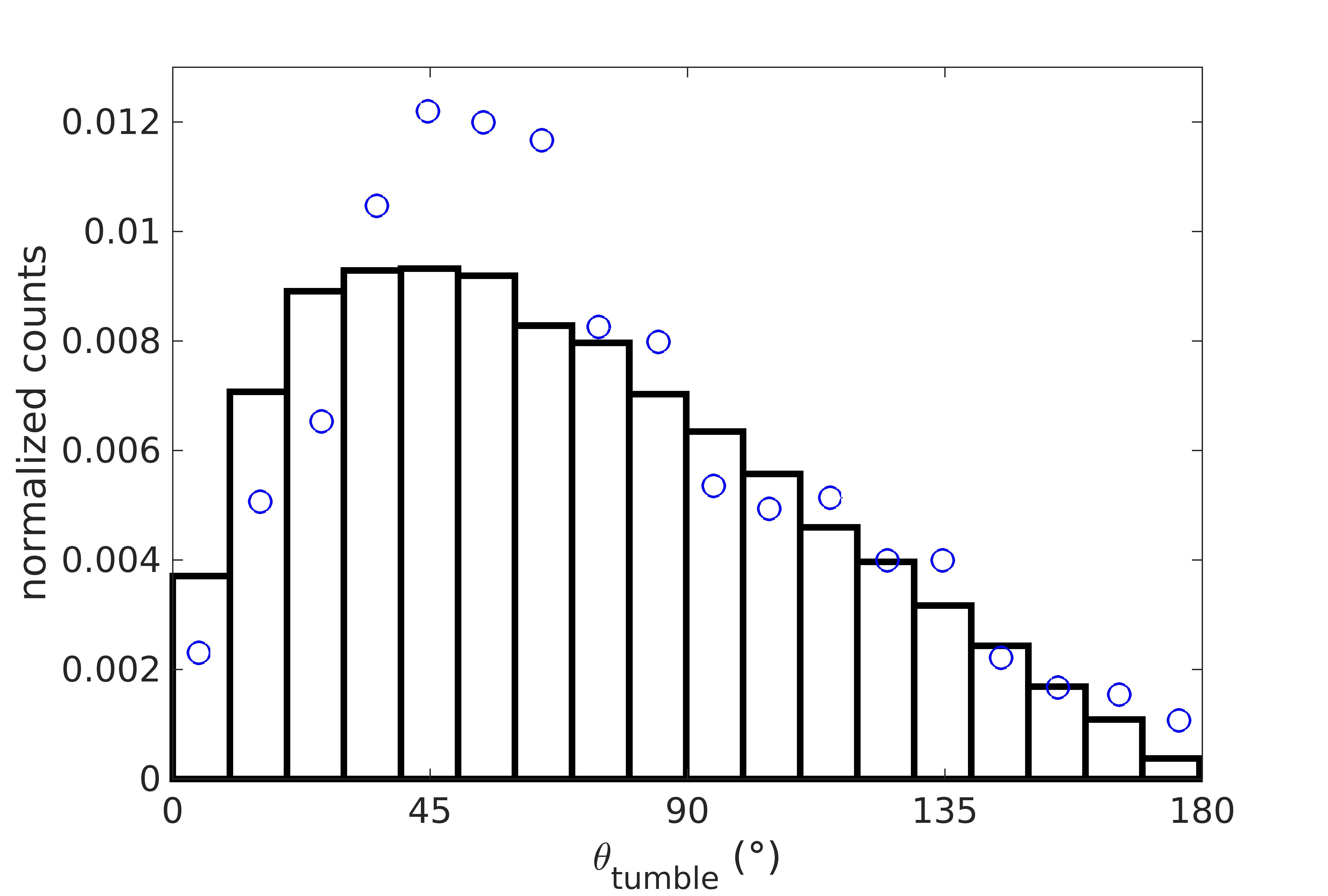

Supplement: S2 Fig — Tumbling has been implemented as an effective rotational diffusion with enhanced noise. The strength of that noise can formally be described by an effective ‘tumbling temperature’, Ttumble. This parameter is chosen such that the resulting mean tumbling angle matches the measured tumbling angle of 〈θtumble〉 = 68° observed for E. coli [28]. To do so we generated 50000 tumbling angles from a simulation of our model for different noise strengths and calculated the mean and standard deviation for each noise strength to find a parameter value that matches the experimental angle. The normalized distribution of the tumbling angle obtained with the best fit parameter Ttumble = 4.2 × 10^4^ K is shown here as black bars, and it is seen to be very close to the experimental distribution of Berg and Brown [28] (blue points), with the same mean and standard deviation (〈θtumble〉 = (68 ± 40)° compared to 〈θtumble〉 = (68 ± 36)° [28]) and a very similar skewness to the right. (TIFF) [file pcbi.1007548.s007.tiff]

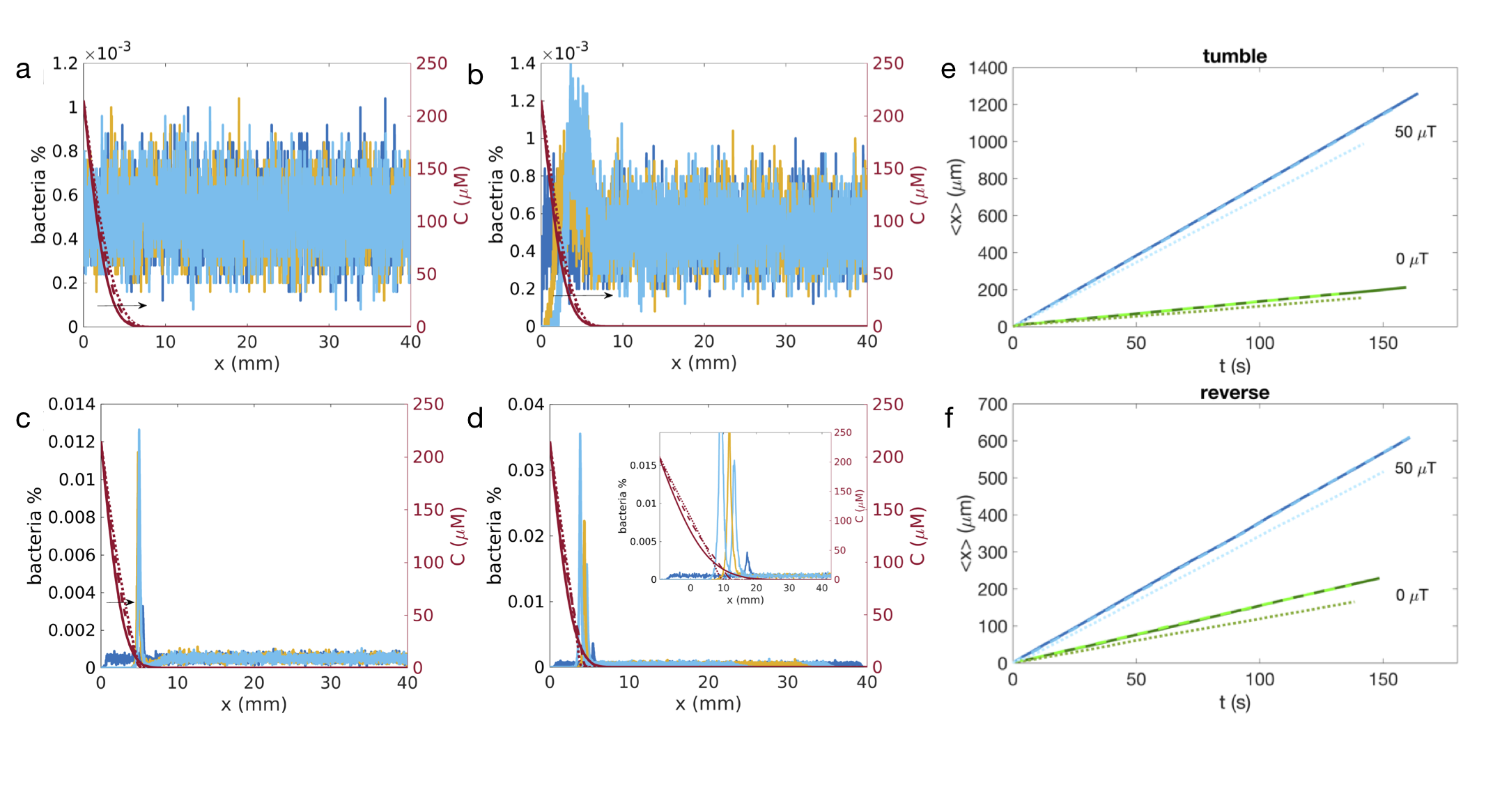

Supplement: S3 Fig — Effect in capillaries. Band at 1 min (blue), 10 min (yellow) and 20 min (light blue) and the corresponding oxygen concentration (filled line, dashed line and dotted line) for a) ∇C0 = 100 μMmm−1, b)∇C0 = 1 μMmm−1, c) ∇C0 = 0.01 μMmm−1, d) Step-function. The black arrow shows the time direction. The inset shows the zoom of the band in d). Here the oxygen concentration is integrated only along the long axis of the capillary and it is considered constant in the cross section of the capillary. The band forms only for values of ∇C0 close to 0, so for simplicity the response function τrun is chosen to be a step function, in accordance with previous works [12, 13]. Effect of ∇C0 on the chemotactuc velocity for a constant gradient. Mean position of 1000 bacteria performing attractive chemotaxis with a constant gradient. The slope of the line gives the chemotactic velocity. The results are obtained in green for 0 magnetic field, and in blue for a parallel magnetic field of 50 μT. Filled lines correspond to ∇C0 = 0 (equivalent to considering a step-function for τrun), dashed lines correspond to ∇C0 = ∇C/10, and dotted lines to ∇C0 = ∇C. For both tumble e) and reverse f) and in all magnetic conditions, it can be seen that chosing ∇C0 = 0 or ∇C0 = ∇C/10 gives the same result, while chosing ∇C0 = ∇C slows down chemotaxis of 15% for tumble and 0 μT, 9% for tumble and 50 μT, 20% for reverse and 0 μT and 8% for reverse and 50 μT. In conclusion, changing the cutoff ∇C0 (thus changing the linear behavior range in trun) affects in minimum part the chemotactic velocity. The effect of ∇C0 for a dynamic gradient in a capillary is explored in the supplementary S3 Fig. Again, changing the gradient cut slows down or speeds up the chemotaxis; the effect is more pronounced for this system. (TIFF) [file pcbi.1007548.s008.tiff]

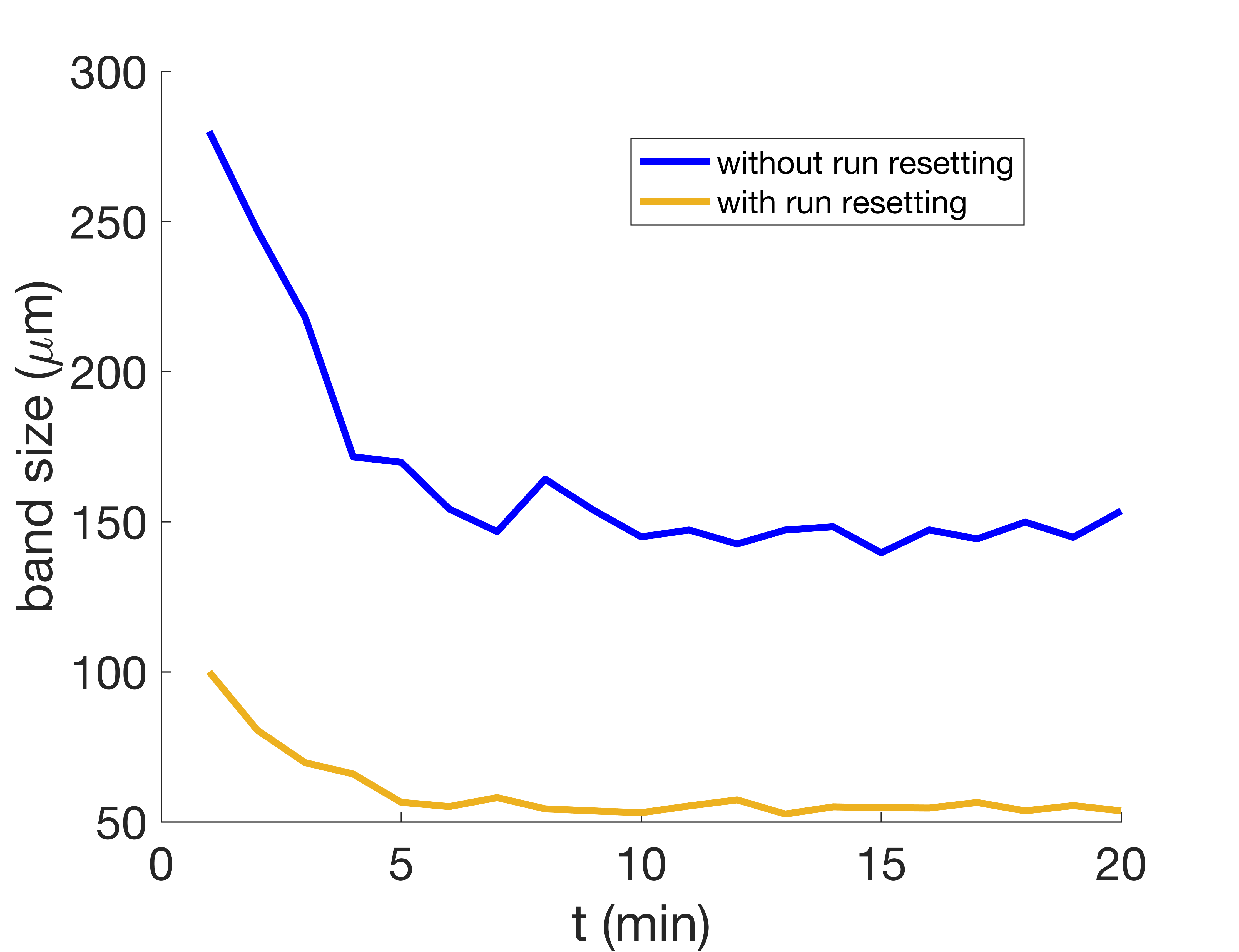

Supplement: S4 Fig — Band size as function of time for the models with and without resetting of the run (yellow and blue, respectively) after crossing the preferred concentration. Without resetting the bacterium is allowed to finish its run after crossing the preferred concentration, while with resetting a new run is started (likely with a smaller run time) when the bacterium crosses the preferred concentration and runs in the unfavorable direction (thus shorter). As it can be seen, resetting results in a smaller band size, which is also necessary to match the experimental data. For these simulations, tup = 6 s, tdown = 1 s and k = 0.01 fmol min−1 cell−1. (TIFF) [file pcbi.1007548.s009.tiff]

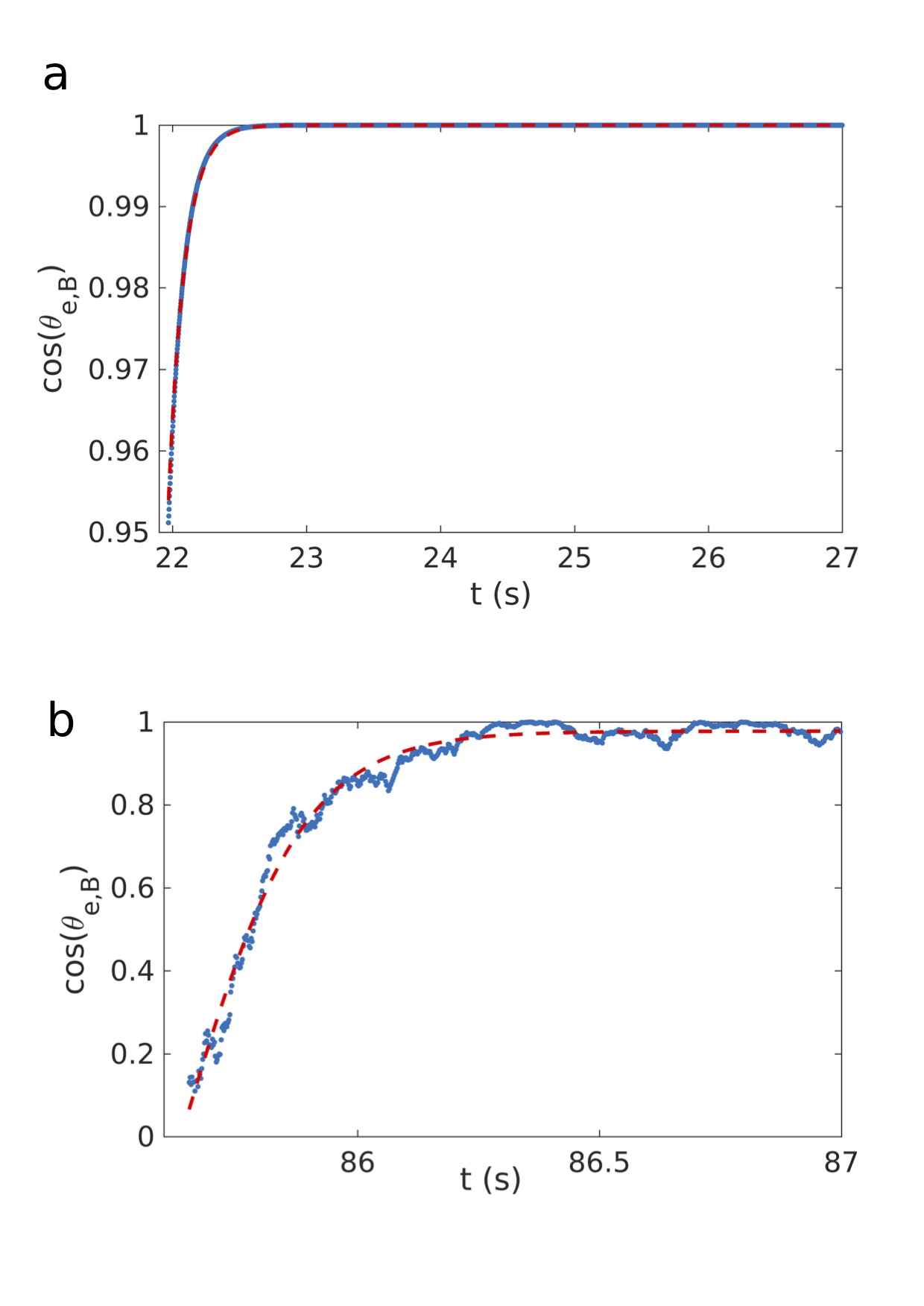

Supplement: S5 Fig — The relaxation of the magnetic alignment after a tumble is described by cos(θe,B)=exp(2t/τ˜)-cexp(2t/τ˜)+c, see S3 Text. As an example, we fit (case a), in red) the cosine of the alignment angle θe,B after a tumble for 500 μt without noise (case a), in blue), the case for which the theory was derived. With the parameters of our simulation, the expected value for β=1/τ˜ is 4.4 s−1. This value is recovered by fitting the simulation data, giving β = 4.1s−1. The fit has been performed with the function f(x)=e2b(x-d)-(1-a)(1+a)-1e2b(x-d)+(1-a)(1+a)-1, where b evaluates β=τ˜-1, a evaluates ez0 and d is needed to re-scale the times to 0. The data have been fitted with f(x) as described in the S3 Text (in red). Then we consider the cosine of the angle θe,B after a tumble event at B = 500 μT in the presence of thermal noise (case b), blue curve). The data have been fitted with g(x) = f(x) + g0 (in red). The constant g0 = 〈cosθe,B〉 reflects the nonzero mean value of the cosine in thermal equilibrium. The fit in this case leads to β ≃ 4.0 s−1, still in good agreement with the theory derived in absence of noise. For a magnetic field of B = 50 μT, the strength of the magnetic field of the Earth, the relaxation constant is β = 0.44 s−1, corresponding to a decay time of τ˜≃2.3s. However, in that case, the fluctuations around that decay are considerably more pronounced when the thermal noise is present. (TIFF) [file pcbi.1007548.s010.tiff]

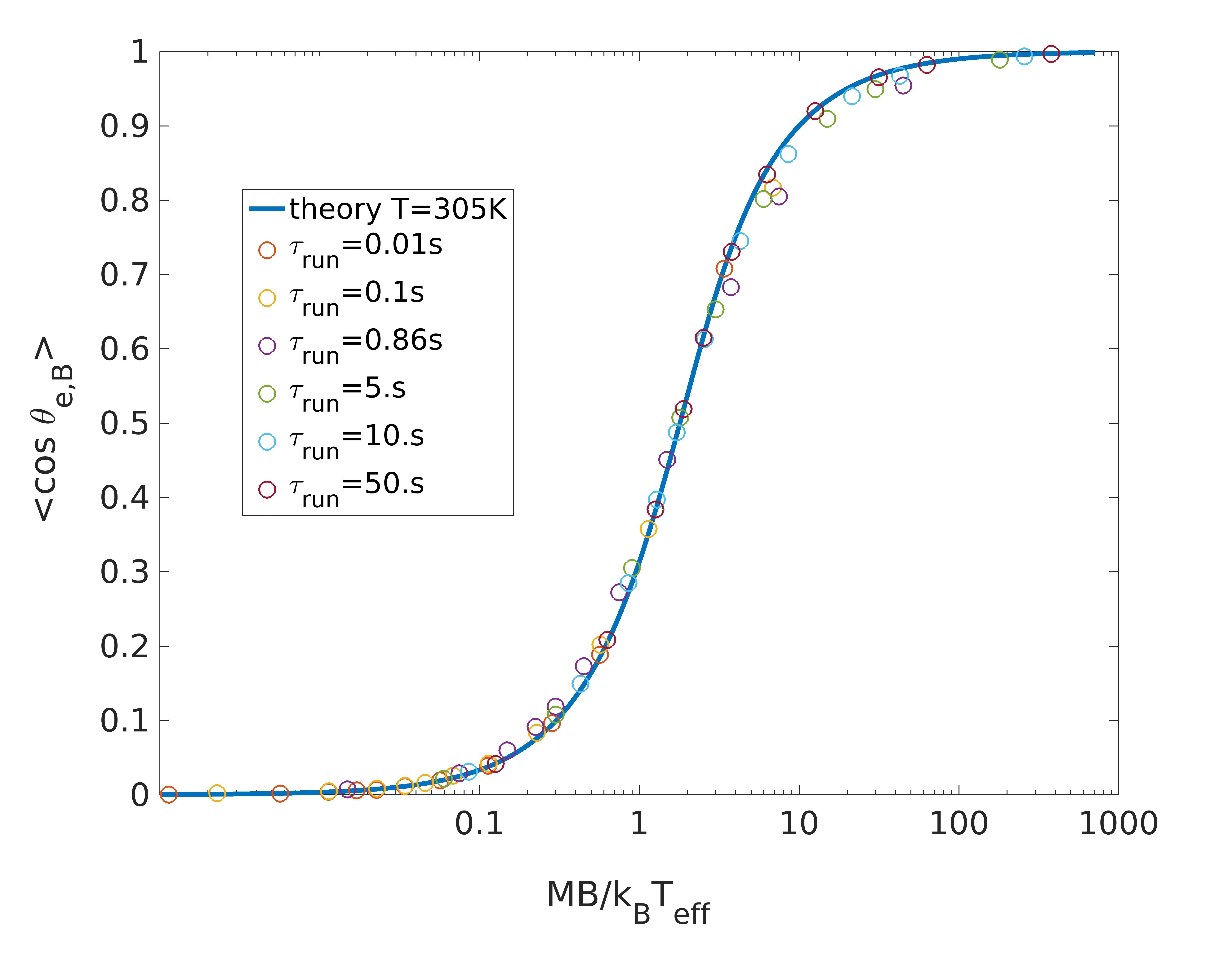

Supplement: S6 Fig — The Langevin curves for the cosine of the alignment angle can be plotted as function of MB/kBTeff, with the effective temperature determined by fitting the Langevin function to the individual curves. In this case, all data for different run times collapse on one Langevin curve, when the cosine of the alignment angle is plotted as function of the effective Langevin parameter MB/kBTeff with a run-time dependent effective temperature. Only small deviations from the theoretical curve are observed, but not as strong as the deviation seen for the histogram of the alignment angle. We emphasize that the effective temperature which we obtain by fitting the Langevin plots is different from the tumbling temperature, it depends on the mean tumble time and on the rotational friction coefficient and generally satisfies T < Teff < Ttumble. (TIFF) [file pcbi.1007548.s011.tiff]

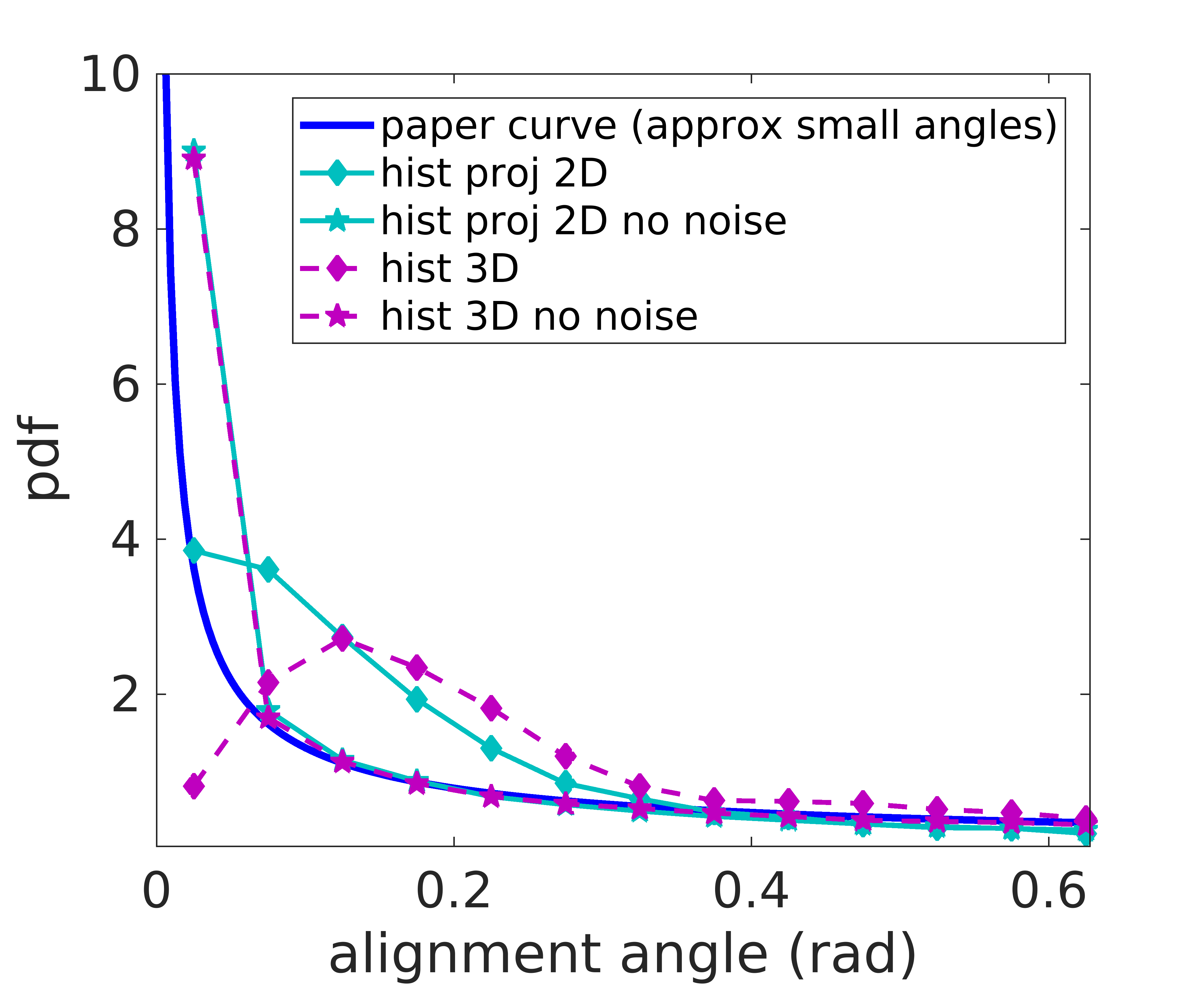

Supplement: S7 Fig — Alignment angle for run and tumble at B = 500 μT, for 3D motion (purple curve) with and without thermal noise during runs (diamonds/stars) compared to a 2D projection (blue curve) with and without thermal noise (diamonds stars). While 3D and the 2D projections differ from each other when thermal noise during runs is present, when thermal noise is taken away the two cases coincide, and they show the velocity condensation phenomenon described by Rupprecht et al. [44]. In dark blue, the theoretical curve at small angles Pθ θ−1+KT/mB [44]. The effect they describe results true only without thermal noise. (TIFF) [file pcbi.1007548.s012.tiff]

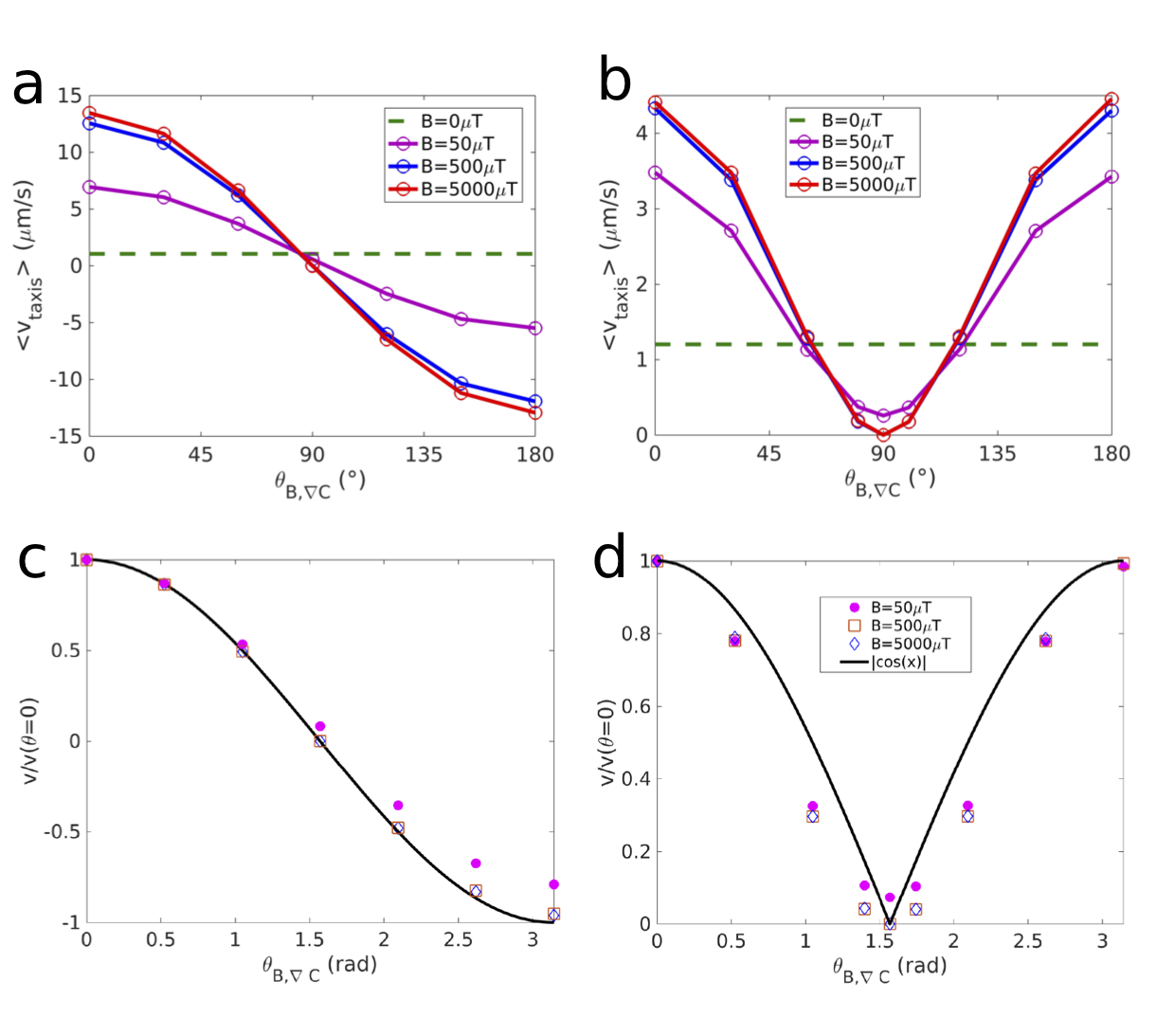

Supplement: S8 Fig — Chemotactic velocity as a function of the field inclination relative to the gradient for tumble (a) and reverse (b) at different magnetic field velocities. For a very strong magnetic field and tumble, we see that in the parallel case, the self velocity of 14.2 μm is reached. For run and reverse, the velocities are always smaller than for the corresponding tumble case at 0°. The same plots are presented in (c) (tumble) and (d) (reverse), but here they are collapsed since now the velocity is normalized to 1. In black, the cosine of the magnetic angle for the tumble and of the absolute value of the cosine for reverse. Higher magnetic field follow the theoretical cosine better. (TIFF) [file pcbi.1007548.s013.tiff]

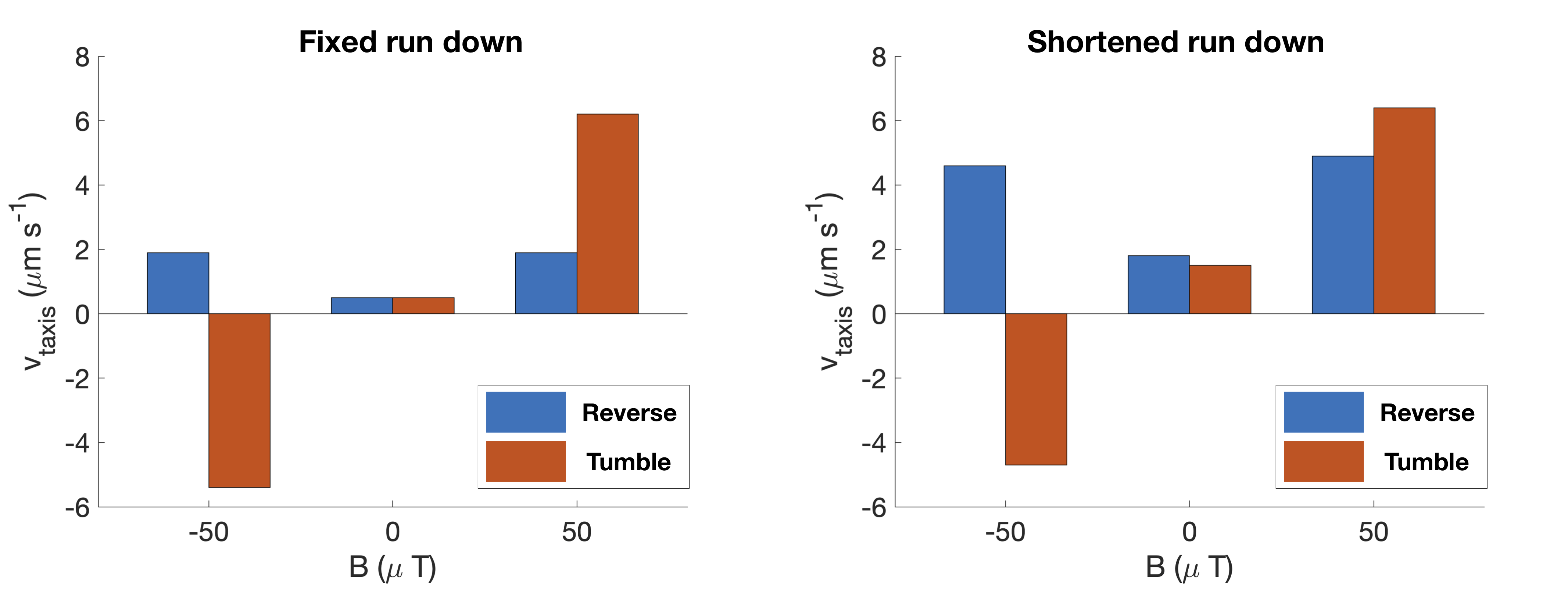

Supplement: S9 Fig — We tested whether shortening runs down the gradient changes our results. When the bacterium is running down the gradient, in the model used in the main text the runs have a fixed duration (left Fig), in this case of 0.5 s, while the maximal run time up the gradient is of 1.5 s. In the right Fig instead, the runs down the gradient are shortened following a linear decrease up to a value of 0.5 s, reached at −∇C0 (right). The advantage of tumbling over reversals in the case of a parallel field is also seen with shortened runs down the gradient, but is less pronounced than without the shortening. (TIFF) [file pcbi.1007548.s014.tiff]

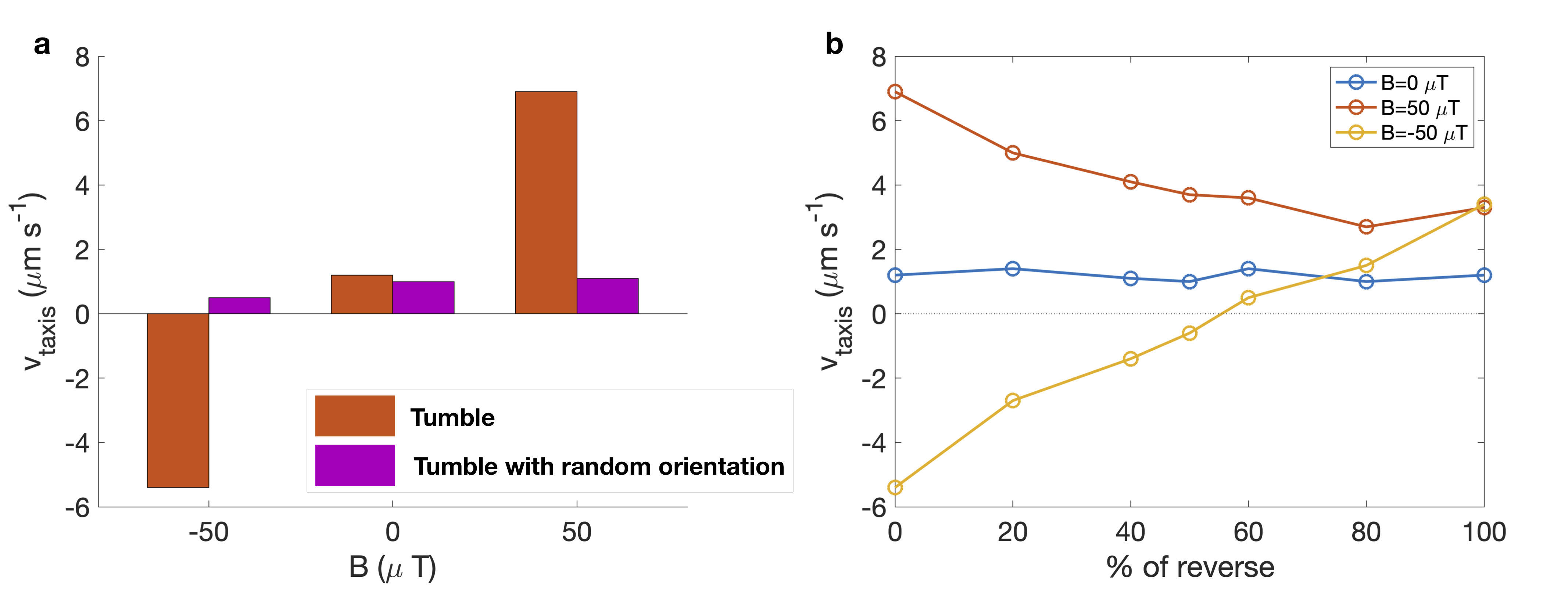

Supplement: S10 Fig — We tested two different mechanisms that combine tumbles with reversals. (a) In the first case, after each tumble the direction of motion is chosen randomly as either parallel or antiparallel to the body orientation mimicking the formation of a flagellar bundle on either side of the body (data in purple). In that case, motion up the gradient under an antiparallel magnetic field is possible, but slower than in the parallel case and slower than in the absence of the field. For comparison, tumble without the randomization of the direction of motion is shown in orange. (b) In the second case, the bacterium performs tumbles or reversals with certain probabilities at the end of each run. We simulated a combination of reversals and tumbles that interpolates smoothly between the two types of behaviors. We varied the fraction of reversals and found that swimming up the gradient against a magnetic field requires more than 50% reversals. (TIFF) [file pcbi.1007548.s015.tiff]

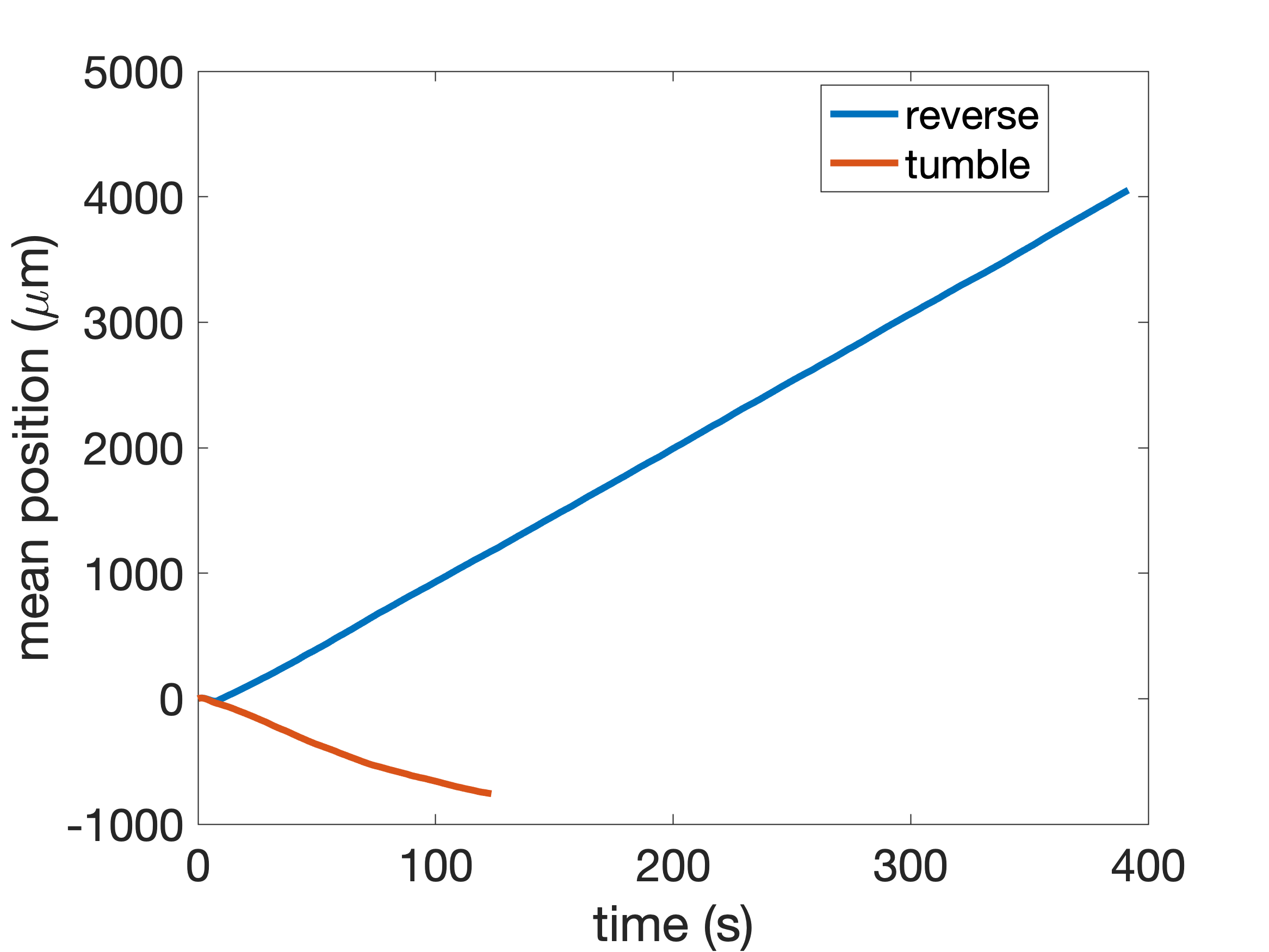

Supplement: S11 Fig — We tested a scenario where runs up the gradient are very long and cells spend almost all time running as expected for a nonlinear chemotactic regime. Here, the run times up the gradient are 20 s, while the run times downward are 1 s. The averaged position of the bacteria as function of time is plotted for reverse and tumble. Positive values correspond to bacteria climbing up the gradient. Here, an antiparallel magnetic field is used and 100 cycles of run and change of directions are plotted. Reversing bacteria practically run always up the gradient with very long runs, while tumbling bacteria are forced to follow the antiparallel magnetic field, and are thus forced to swim down the gradient with very short runs. (TIFF) [file pcbi.1007548.s016.tiff]

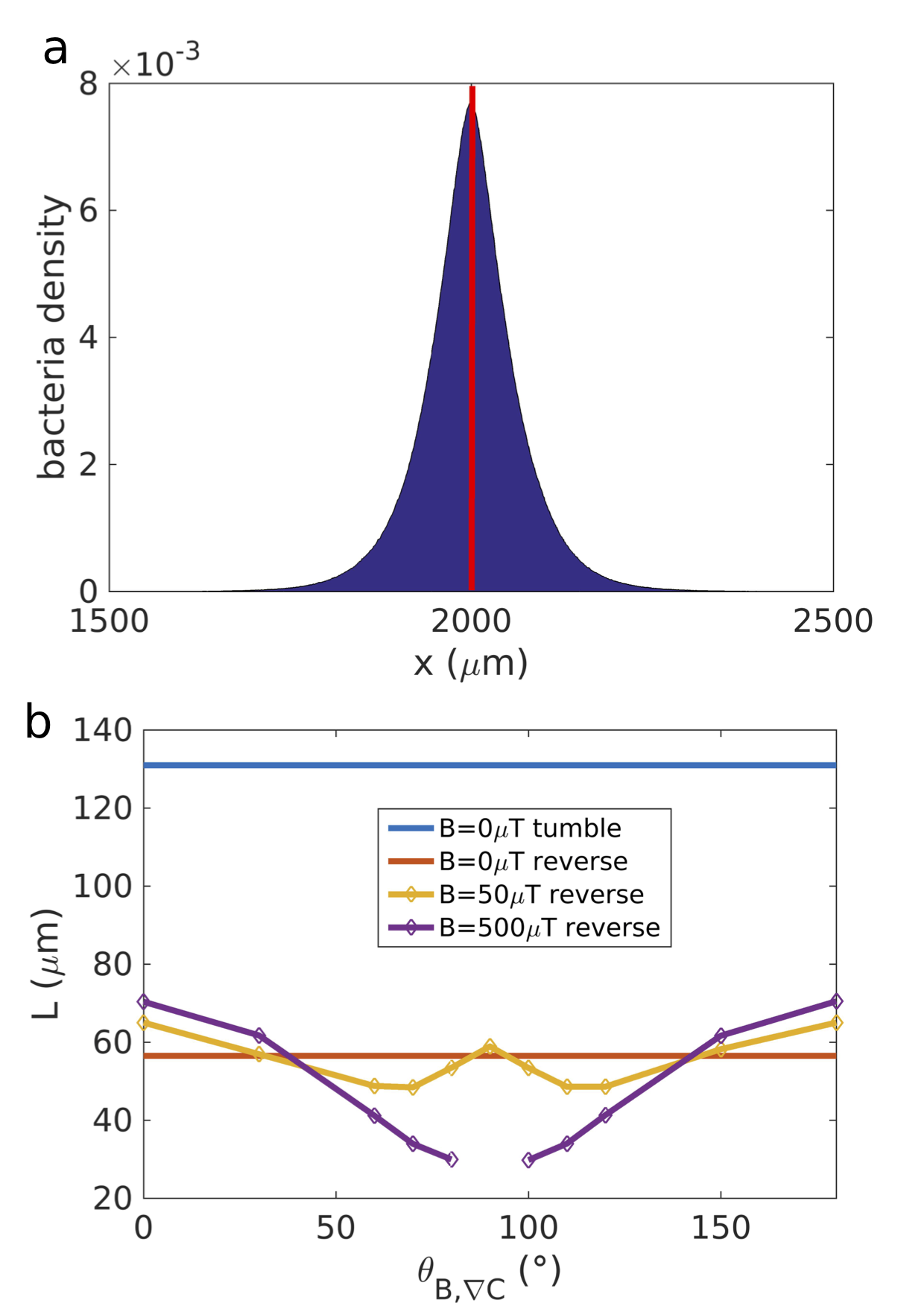

Supplement: S12 Fig — The size of the band is estimated in the main paper as the standard deviation of the position of the bacteria along the gradient. An alternative way to estimate it is the following: the density profile of the band after the equilibrium time (in this case for run and reverse of 477 s) is obtained (Fig (a)), integrating over all time-steps after the equilibration and normalizing it by the number of time-steps and number of bacteria. The profile can be well fitted with a Laplace distribution f(x)=12Lexp(-|x-m|L), where the free fitting parameters are m, the position of the preferred concentration and L, the decay length of the curve. The curve is symmetrical with respect to the preferred concentration (which is situated at 2000 μm and indicated by the red line for this case without magnetic field). L is thus a good estimator of the size of the band, since 68% of the density is situated in [m − L, m + L]. The bands seen in the simulations have a symmetric shape and their density profiles decay on both sides with the same decay length (this has been tested by fitting both sides separately with an exponential). Comparing the two approaches, we notice that the results show the same trends, but using the Laplace distribution fit leads to systematically smaller values. This is due to the fact that the standard deviation is influenced by the long tails of bacteria that are not in the band, which increase the estimate of the band width compared to L. In Fig (b), we show the band width obtained by the fit with the Laplace distribution for different magnetic field intensities, as a function of the magnetic field orientation. (TIFF) [file pcbi.1007548.s017.tiff]

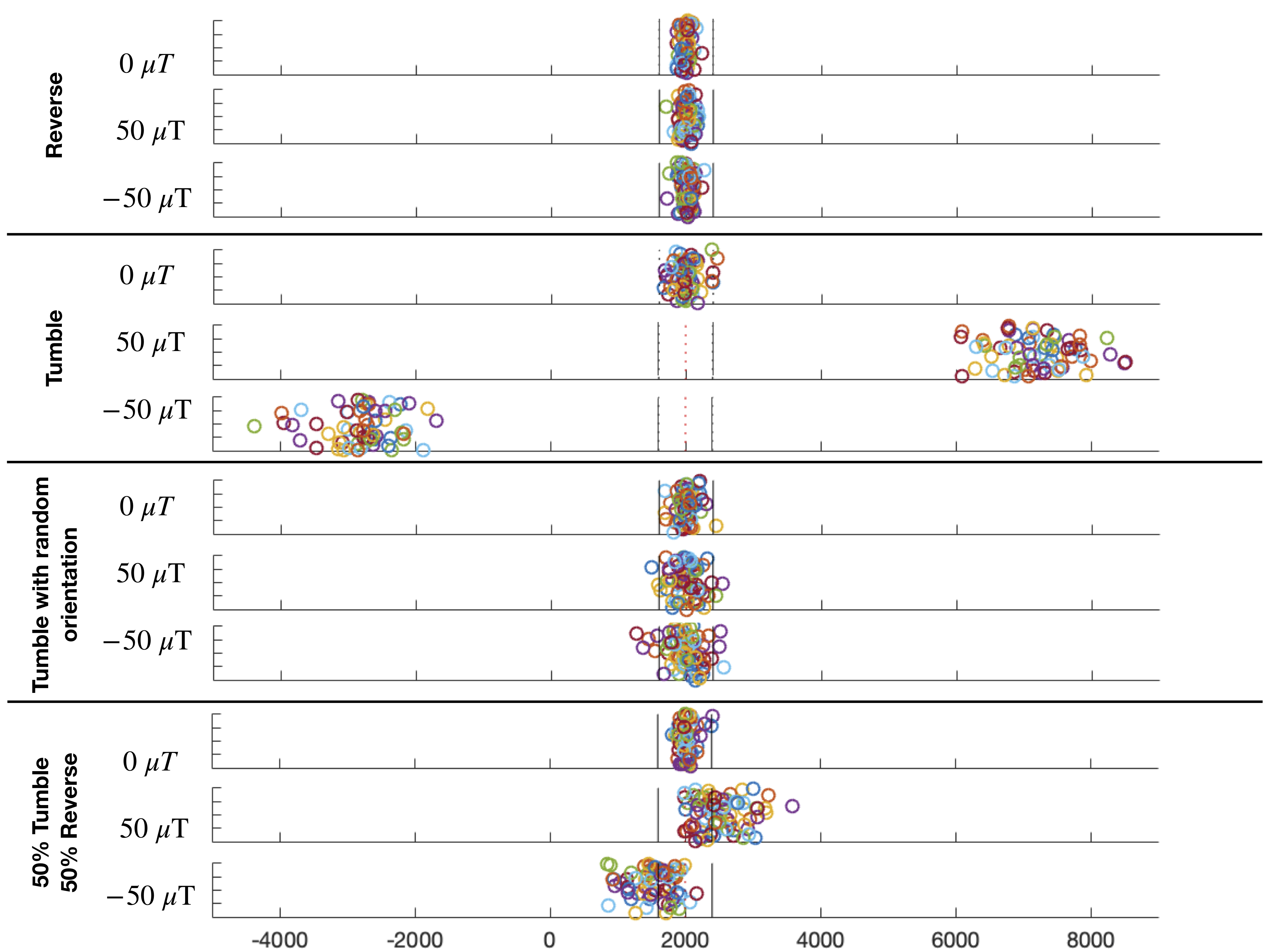

Supplement: S13 Fig — Band formation in a constant gradient for reverse, tumble, tumble with random change in orientation and mix of 50% reverse and 50% tumble at different magnetic field intensities. In the plot, the position of 100 bacteria is shown after 500 cycles of run and change of direction. The bacteria are initial distributed randomly within the black box at [1600, 2400] μm. The preferred concentration is situated at 2000 μm (red dotted vertical line). The x axis is in μm, y axis has size of 800 μm. While reverse and tumble with random orientation can form the band in any condition, pure tumble and a mixture of tumble and reverse perform chemotaxis poorly and do not form a band that is well localized around the preferred concentration. (TIFF) [file pcbi.1007548.s018.tiff]

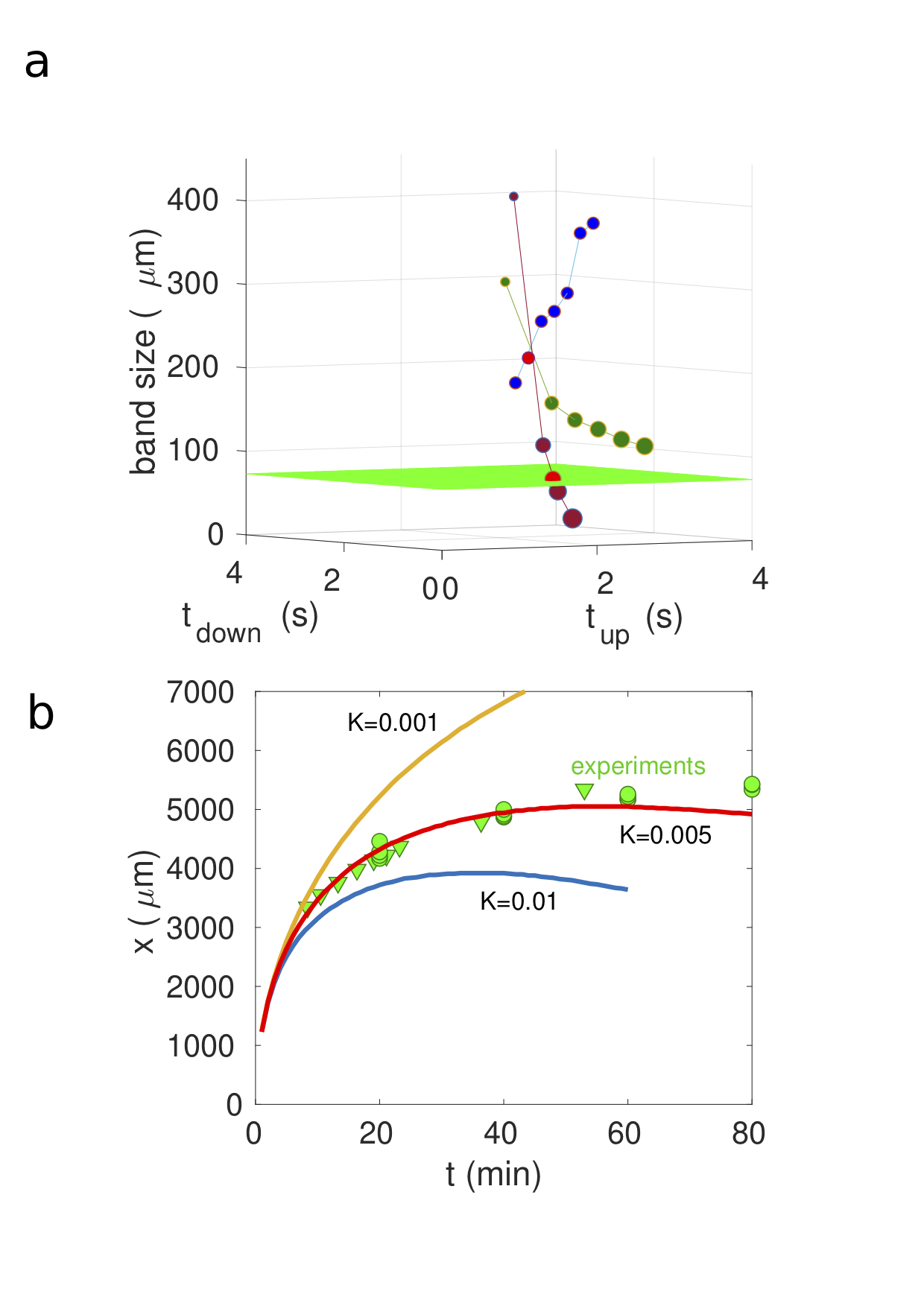

Supplement: S14 Fig — The model of the capillary assay presents three free parameters that could not be determined by the experiments: tup the run time towards a favorable direction, tdown towards an unfavorable direction, and the oxygen consumption k. First we varied the run times to get the desired band size (Fig a). We plot here the band size as function of the two run times for an antiparallel field of 50 μT; the green plane corresponds to the desired experimental value. The blue points were obtained keeping tdown constant; the green points, keeping tup constant; and the red points, keeping the ration between the two times constant. The winning point is the bright red case, obtained for tup = 2 s and tdown = 0.9 s with k = 0.01 fmol min−1 cell−1 (see [12, 13]). The point size is proportional to the ratio of the times. Then, to match the band position dynamics (Fig b), the consumption constant k is varied. Smaller consumption constants make the dynamics slower. To match the experimental data points in green, we chose k = 0.005 fmol min−1 cell−1. (TIFF) [file pcbi.1007548.s019.tiff]

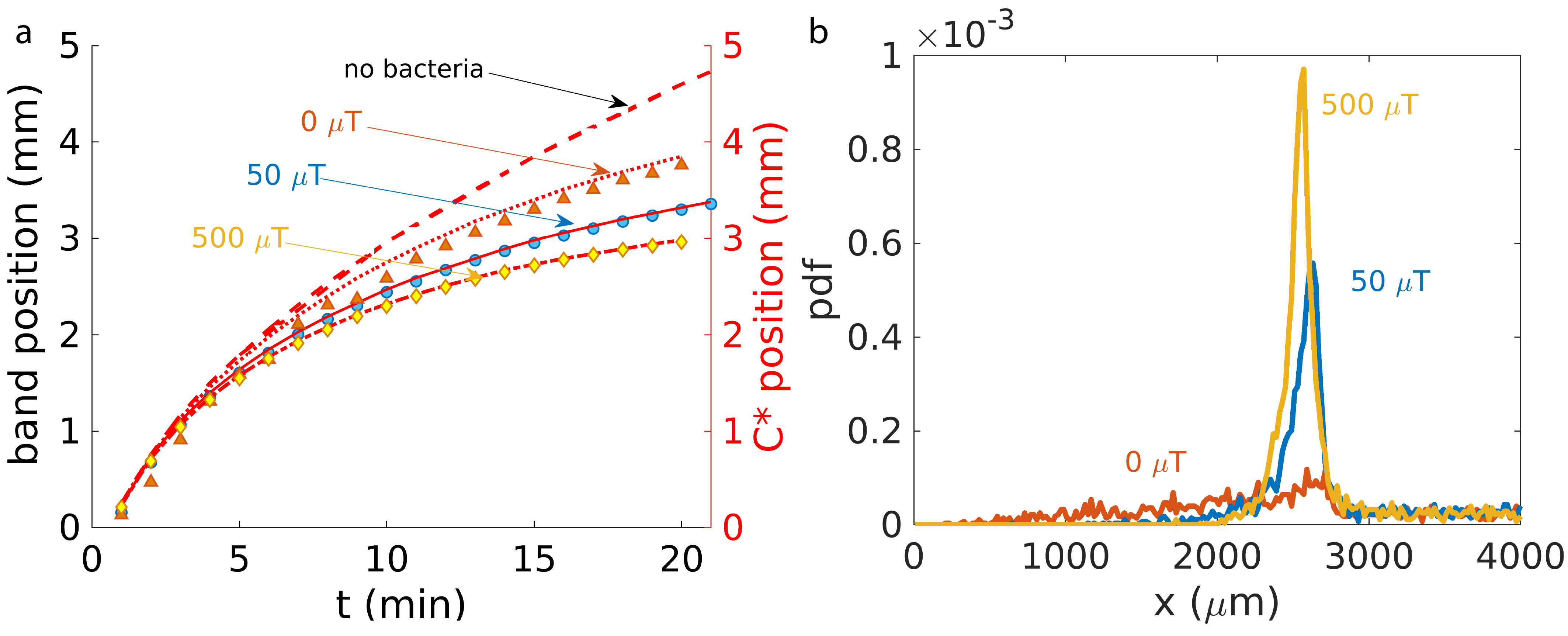

Supplement: S15 Fig — Since the model provides an effective description of the system, the effect of the magnetic field can be explored also in this capillary assay. The dynamics of the band position depends on the magnetic field intensity (a): stronger magnetic fields stabilize the band closer to the air-water interface thanks to the interplay between aerotaxis, oxygen consumption and magnetic fields. The band position is represented by the data points and the preferred concentration (C*) position by the red lines. Magnetic fields do not influence the band position at small times (below 5 min), when the leading dynamics is the oxygen flow as it can be seen from Fig (a), where pure oxygen flow -dashed red curve- matches the curves in the presence of bacteria. On the contrary, the magnetic field speeds the formation of the band up at small times before reaching the equilibrium: the band formed with the Earth magnetic field (blue curve of Fig (b) and with 500 μT (yellow curve) is already symmetrical at 5 min and more dense compared to the band formed without magnetic field (in red). Therefore, in agreement with the results for a constant gradient, antiparallel magnetic fields help the band formation. (TIFF) [file pcbi.1007548.s020.tiff]
