# Supplementary material for: Chemotaxis in external fields: Simulations for active magnetic biological matter
Source: PLoS Comput Biol. 2019 Dec 19;15(12):e1007548. doi: 10.1371/journal.pcbi.1007548 (PMC6941824; doi:10.1371/journal.pcbi.1007548)
Supplement: S1 Table — Simulation parameters for free swimming bacteria. Some comments are in order with respect to these parameters. (i) The friction coefficients are calculated assuming a spherical shape of the bacterium/microswimmer. The radius of the sphere was chosen to be 1μm, a values compatible with the dimension of E. coli bacteria [28], as well as of some magnetotactic bacteria, in particular strain MSR-1 [11]. (ii) The magnetic moment was chosen to match the one of MSR-1 [57]. Here, a magnetic crystal with size 25 nm possesses a magnetization of ≃ 3.1 × 10^−17^ Am2. Considering a typical crystal of MSR-1 [58, 59] with radius 50nm, and considering a mean of 20-30 crystals per bacterium [11], we obtain the final value of 0.6 × 10^−3^ A μm2, in good agreement with the magnetic moment calculated in [43]. (iii) Regarding the swimming speed, the mean run time with and without chemical gradients, and the mean tumble time, the parameters were chosen as measured for E. coli [28]. The parameters for magnetotactic bacteria have not been measured at the same level of detail, but swimming speeds tend to be larger and to vary greatly between studied strains [12]. (iv) The gradient was chosen constant in space and time, with a value similar to the serine gradient experienced by the bacteria in the experiments of Berg and Brown [28]. Oxygen gradients that built up dynamically in experiments with magnetotactic bacteria were seen to have similar values [12]. The preferred concentration was chosen in the range of oxygen concentration preferred by microaerophilic bacteria [13, 60]. (PDF) [file pcbi.1007548.s021.pdf]

name	object	value	reference
a	cell size (sphere radius)	1 μm	[2]
T	room temperature	305 K	[2]
T_{tumble}	effective tumbling temperature	4.2×10^4 K	see Fig. S1
M	magnetic moment modulus	0.6×10^{-3} A μm^2	[4, 6]
v	self velocity modulus	14.2 μm s^{-1}	[2]
η	water viscosity	0.027 Pa s	[3]
γ_{t}	translational friction coefficient	5.1×10^{-8} Kg s^{-1}	calculated from a
γ_{r}	rotational friction coefficient	6.8×10^{-8} Kg s$^{-1}\mu$m^2	calculated from a
τ_{run},τ_{reverse}	mean run/reverse time in the absence of chemicals	0.86 s	[2]
τ_{tumble},τ_{pause}	mean tumble/reversal-pause time in the absence of chemicals	0.14 s	[2]
τ_0	mean run time with chemicals and no gradient	1.48 s	[2]
t_{up}	max. mean run time up a gradient	$2\tau_0$	
t_{down}	min. mean run time down a gradient	τ_0	
∇C	modulus of the gradient	25 μM mm^{-1}	[2]
∇C_0	modulus of the reference gradient	25 μM mm^{-1}	the same as ∇C
C^*	preferred concentration at position $x = 0$	10 μM	[1, 5]
dt	time-step of integration	0.002 s	

[1] M. Bennet, A. McCarthy, D. Fix, M. R. Edwards, F. Repp, P. Vach, J. W. C. Dunlop, M. Sitti, G. S. Buller, S. Klumpp, and D. Faivre. Influence of magnetic fields on magneto-aerotaxis. *PLOS ONE*, 9(7):1–10, 07 2014.

[2] H. C. Berg and D. A. Brown. Chemotaxis in *Escherichia coli* analysed by three-dimensional tracking. *Nature*, 239:500–504, 1972.

[3] Howard C. Berg. Random walks in biology. *Princeton Paperbacks*, 1993.

[4] S. Klumpp, B. Kiani, P. Vach, and D. Faivre. Navigation with magnetic nanoparticles: magnetotactic bacteria and magnetic micro-robots. *Physica Scripta*, 2015(T165):014044, 2015.

[5] C. T. Lefèvre, M. Bennet, L. Landau, P. Vach, D. Pignol, D. A. Bazylinski, R. B. Frankel, S. Klumpp, and D. Faivre. Diversity of magneto-aerotactic behaviors and oxygen sensing mechanisms in cultured magnetotactic bacteria. *Biophysical journal*, 107(2):527 – 538, 2014.

[6] R. Nadkarni et al. A comparison of methods to measure the magnetic moment of magnetotactic bacteria through analysis of their trajectories in external magnetic fields. *PLOS One*, 8 (12), 2013.
