# Supplementary material for: Chemotaxis in external fields: Simulations for active magnetic biological matter
Source: PLoS Comput Biol. 2019 Dec 19;15(12):e1007548. doi: 10.1371/journal.pcbi.1007548 (PMC6941824; doi:10.1371/journal.pcbi.1007548)
Supplement: S2 Table — Simulation parameters for capillary assays. If not stated here, the parameters are unchanged with respect to S1 Table. For the capillary assay, the oxygen concentration and gradient are dynamic and not constant. The run-time response function to the gradient is a step function, thus the reference gradient ∇C0 is 0. (PDF) [file pcbi.1007548.s022.pdf]

**name**	**object**	**value**	**reference**
N_{bact}	number of bacteria	13900	from experiments
v	self velocity modulus	50 μm s^{-1}	tracking of the videos
C^*	preferred concentration at position $x = 0$	3 μM	[1, 2]
k	oxygen consumption rate	0.005 fmol min^{-1} cell^{-1}	tuned to match experiments
$C(x = 0)$	oxygen concentration at $x = 0$	216 μM	[1, 2]
C_{a}	concentration cutoff	0.75 μM	[1, 2]
D_{O_2}	oxygen diffusion constant	2100 μm$^2 s^{-1}$	[1, 2]
t_{up}	max. mean run time up a gradient	2 s	tuned to match experiments
t_{down}	min. mean run time down a gradient	0.9 s	tuned to match experiments

[1] M. Bennet, A. McCarthy, D. Fix, M. R. Edwards, F. Repp, P. Vach, J. W. C. Dunlop, M. Sitti, G. S. Buller, S. Klumpp, and D. Faivre. Influence of magnetic fields on magneto-aerotaxis. *PLOS ONE*, 9(7):1–10, 07 2014.

[2] C. T. Lefèvre, M. Bennet, L. Landau, P. Vach, D. Pignol, D. A. Bazylinski, R. B. Frankel, S. Klumpp, and D. Faivre. Diversity of magneto-aerotactic behaviors and oxygen sensing mechanisms in cultured magnetotactic bacteria. *Biophysical journal*, 107(2):527 – 538, 2014.
